# Women and health providers’ perspectives on male support for cervical cancer screening in Gwanda district, Zimbabwe

**DOI:** 10.1371/journal.pone.0282931

**Published:** 2023-10-12

**Authors:** Fennie Mantula, Yoesrie Toefy

**Affiliations:** 1 Department of Global Health, Division of Health Systems and Public Health, Faculty of Medicine and Health Sciences, Stellenbosch University, Tygerberg, Cape Town, South Africa; 2 Department of Nursing and Midwifery, Faculty of Medicine, National University of Science and Technology, Bulawayo, Zimbabwe; University of Zimbabwe Faculty of Medicine: University of Zimbabwe College of Health Sciences, ZIMBABWE

## Abstract

Several studies have shown that male involvement increases the uptake of sexual and reproductive health programmes for improved family health outcomes. The role of men in reducing the burden of cervical cancer has however not been researched in Zimbabwe. It is for this reason that this study explores male support for cervical cancer screening programmes from the perspective of women and health providers in the Gwanda district of Zimbabwe. A qualitative approach that engaged thirty-six women aged 25–50 years in focus group discussions, and twenty-five health providers with different roles in the cervical cancer screening programme in in-depth interviews was used to determine their perspectives on male support for cervical cancer screening. Data were analysed thematically using ATLAS.ti Computer Assisted Qualitative data analysis software. Key findings reflected that men lacked knowledge on cervical cancer and its risk factors and prevention. Subsequently, they engaged in sexual behaviours that increased their partners’ risk of acquiring Human Papillomavirus infection, the virtually necessary cause of cervical cancer. Furthermore, men did not provide the necessary emotional and financial support for their women to access screening and treatment. Participants were optimistic that innovative awareness creation strategies and intense, sustained cervical cancer education efforts targeting men could increase male partner support. Involvement of community leaders was seen as crucial in the facilitation of male involvement for programme acceptance and improved uptake of cervical cancer screening. Male involvement is seen as an integral component of the cervical cancer prevention and control programme that has to be implemented in Gwanda district to minimise male partner-related barriers to cervical cancer screening. Further research focusing on men is required to identify specific knowledge gaps that would enable development of appropriate strategies that best involve men in cervical cancer prevention and control interventions.

## Introduction

Male involvement is increasingly being recognised as central to the successful implementation of maternal and child health programmes worldwide [1]. This assertion is premised on the decision-making role men exercise within families and relationships, and the influence they have on women’s health-care decisions [2]. The lack of men’s support for sexual and reproductive health (SRH) programmes that include cervical cancer screening is seen as a barrier to utilisation of health services by most women [3].

While a progressive reduction in cervical cancer incidence and mortality has been observed in high-income countries due to effective screening and treatment programmes, the reverse holds true in most low and middle income countries (LMICs) [4]. A systematic review on factors that affect uptake of cervical cancer screening in LMICs revealed that lack of knowledge and awareness, psychological barriers that include fear of pain, structural barriers such as unaffordable and inaccessible screening services, and socio-cultural and religious beliefs including husbands’ and family members’ objection, prevent women from screening [5].

Men seemingly have a pivotal role to play in reducing the burden of cervical cancer [6]. Providing financing for women’s transportation to screening sites, emotional support and encouragement for their partners, and adherence to post treatment recommendations if precursor lesions are detected during screening (abstinence from sex for a specified period) offer an incentive for women to take up screening [2]. Inversely, male partners’ disapproval of screening for varied reasons limits the prospects of women seeking the service.

Previous research has shown that men are keen to participate in SRH programmes that enable them to provide spousal support for health promotion activities [7]. Moreover, their willingness to support their partners is correlated with increased uptake of SRH programmes such as family planning, voluntary Human Immunodeficiency Virus (HIV) counselling and testing, improved spousal communication and maternal health [7]. Similarly, positive attitudes towards cervical cancer screening have been observed with men showing willingness to provide spousal support for their women in utilising the service [6, 8]. The limiting factor however, is their lack of knowledge on cervical cancer, how it can be prevented and the methods used for screening [6, 8–10].

Men therefore indirectly add onto the burden of cervical cancer since the minimal and often incorrect knowledge they possess does not empower them to protect their partners from acquiring Human Papillomavirus (HPV), nor encourage them to get screened [10]. Equally important, men increase the likelihood of their sexual partners developing cervical cancer since a woman’s risk is dependent less on her own sexual behaviour than that of her male partner [11]. The risk is most likely affected by an increase in the chances of exposure to HPV from a high risk sexual partner [12].

Current scientific evidence compellingly demonstrates that voluntary medical male circumcision (VMMC) has a high protective effect against cervical cancer since circumcised men are less likely to harbour HPV under their foreskins [13]. The circumcised status of a sexual partner is thus associated with a reduced risk of cervical cancer [11, 13]. Knowledge of this information could facilitate change in sexual behaviour and motivate the practice of circumcision among men to reduce transmission of oncogenic types of HPV, the necessary causative organism for development of cervical cancer.

Male involvement in cervical cancer programmes is fundamental in reducing the incidence and deaths from this disease [6]. Most men however consider safe motherhood issues to be the responsibility of women [3] and consequently show indifference on issues that relate to cervical cancer screening. Women may even be blamed for a screen result that shows presence of precursor lesions [10]. The World Health Organization (WHO) hence recommends education of males on cervical cancer prevention and control to enhance support of their female partners’ decisions on screening [4].

There is dearth of information on the extent to which men contribute to women’s utilisation of the visual inspection with acetic acid and cervicography (VIAC) programme, the national screening method used in Zimbabwe. This study therefore explored women and health providers’ perspectives on male support for cervical cancer screening in Gwanda district of Zimbabwe. Findings could serve to develop strategies for active male involvement in cervical cancer prevention and control programmes thus contributing to minimising barriers to screening.

## Methodology

This study is embedded within a broader research project that explored barriers to cervical cancer screening in Gwanda district, Zimbabwe. An explanatory design that uses the methodological complementarity of sequential quantitative and qualitative approaches was employed. The study was conducted in a two phased approach using first the quantitative then the qualitative methods.

The first phase conducted in June and July 2019 was a cross-sectional survey which used a semi-structured researcher-administered questionnaire to collect quantitative data from 609 healthy women selected through multistage sampling (Table 1). Under the guidance of a biostatistician, a sample size that would contribute to providing scientific validity to answer the research question was calculated at 628 participants for the quantitative phase of the study using the formula: $n=\left[t^{2}\times \frac{p\times q}{d^{2}}\right]\times DEFF$ where n = sample size, t = 2.045 (linked to 95% Confidence Interval for cluster sampling), p = expected prevalence (fraction of 1), q = 1- p (expected non-prevalence), d = relative desired precision and DEFF = Design effect. The calculation assumed a 50% prevalence of barriers to cervical cancer screening, desired precision of 5%, 95% Confidence Interval and a design effect of 1.5, which is a correction factor that accounts for the heterogeneity between clusters with regard to the measured indicator in cluster sampling. Of the 628 women recruited, 609 (97%) participated in the survey. The remaining 3% cited lack of time as the reason for non-participation.

**Table 1:** Sampling process for the quantitative phase from which FGD participants were drawn.

	Sampling stage 1: Stratification of the district according to residential location as used in the electoral process				
		Urban	Rural	Mine	Total
Strata		1	1	1	3
Number of electoral wards in each stratum		10	24		34
	Sampling stage 2: Simple Random selection of electoral wards proportionate to size of strata				
Number of electoral wards sampled		3	6	1	10
	Sampling stage 3: Stratified Random selection of villages and suburbs in each selected ward proportionate to size of the strata				
Number of villages sampled		3	6	1	10
	Sampling stage 4: Purposive selection of rural households (no updated sampling frame), simple random sampling of urban and mine households (Probability proportionate to size approach was used in selecting households within the selected villages. This was dependent on the number of households in each village or suburb)				
Number of households/women sampled		209	368	32	609

The second phase from which this study derives was conducted in January 2021. A qualitative approach using focus groups discussions (FGDs) and in-depth interviews (IDIs) to better understand the extent to which men support their partners on cervical cancer screening processes was used.

### Setting

This study was conducted in Gwanda district located in Matabeleland South, one of the ten provinces of Zimbabwe. The district consists of 34 electoral wards that include urban, rural and mining areas. There are 30 health facilities within the district, that is; one provincial hospital, one urban clinic, and 28 rural based primary health facilities. Cervical cancer screening services are offered at the provincial hospital and urban clinic since 2013 and 2020 respectively. Periodical screening services are also provided to rural health facilities through mobile clinics. Matabeleland South Province within which Gwanda district is located ranks among the country’s three provinces with the lowest screening rates, reported at 13% in the last demographic and health survey conducted in 2015 [14]. This gave the motivation to conduct the study in this district which was also the first to offer screening services in the province using the VIAC method.

### Sampling procedures

Participants for FGDs were purposively selected as a subset from the same group of women that participated in the first phase of the study using the maximum variation sampling technique, also known as heterogeneous sampling [15, 16]. This sampling strategy aimed at drawing out a sample with diverse characteristics that would yield rich information to holistically address the research objective. Health providers from different levels of health care with different roles in the provision of screening services namely awareness creation, educative, administrative and clinical were also purposively selected for IDIs under the guidance of the district health authorities.

### Sample size

Five FGDs were conducted; two each from the urban and rural wards, and one from the mine setting in accordance with the size of the strata. This was based on Hennink et al.’s [17] guidance which suggests that data saturation is reached at the point at which at least one FGD from each stratum is included for studies that use stratification in sampling.

Twenty-five health workers were also selected for in-depth interviews. These included nurses from the provincial hospital’s departments that attend women in their day-to-day operations namely: Maternity Unit, Outpatients Department, Opportunistic Infections Clinic (OIC), Female Ward, Paediatric Ward and Family Health Services Unit. Nurses and Doctors attached to the VIAC clinic and the Hospital and District Health Administrators were also included as key informants. In addition to health providers from the provincial hospital, one nurse from each of the primary health facilities located in the 10 selected study wards, and Community Health Workers (CHWs) servicing the 10 selected villages were also included the study. These were by default cadres in charge of the various units at the time data were collected, and presumed to have up to date information on the cervical cancer screening programme from the periodical refresher courses they attended.

### Data collection procedures

Data collection was conducted over two weeks by the first author and two research assistants trained in data collection procedures from the first phase of the study. Prior to the study, the FGD guide was pretested on a group of women that participated in the quantitative survey in an electoral ward that was not part of this second phase of the study. The instrument was refined to ensure clarity and appropriateness of the questions [18]. FGDs each comprising between five and eleven participants were conducted at community meeting places to explore women’s knowledge, attitudes and practices on cervical cancer screening, and barriers to cervical cancer screening. To maintain privacy, only the participants and research team were present during the discussions. Probes were used to elicit personal, interpersonal (including male partner contribution), community, and health system related factors, and facilitators to the uptake of screening. Discussions which lasted between 60 and 75 minutes were conducted in Ndebele, the local language. As a woman who had experienced the advantages of cervical cancer screening with the support of her spouse, the first author was careful to recognise her personal experiences and be reflexive about them in order to guarantee unbiased research processes and outcomes.

In-depth interviews were conducted by the first author in English for the professional health providers, and in Ndebele for CHWs. The health facility-based participants were interviewed individually at their workplaces during their normal working hours, while CHWs were interviewed at community meeting places soon after the FGDs were conducted. An interview guide was used to explore health providers’ perceptions on women’s knowledge of cervical cancer and screening, accessibility of screening services, demand creation strategies employed, barriers to screening, and recommendations for increasing the uptake of screening with probes applied to solicit information on male partner involvement. The interview guide which took 30–45 minutes to administer was first pretested on five health providers in non-participating health facilities, then refined for the study. Both FGDs and IDIs were audio-recorded to guard against information loss. Male partner support was thus explored from the perspectives of both women and health care providers.

While there is no quantitative survey component to this study, participants were asked a short set of demographic questions. This allowed for the assessment of the diversity of research participants in fulfilment of the maximum variation sampling technique that was used. Furthermore, a thorough description of participants allows readers and other researchers to determine to whom research findings generalise [19].

Note should be taken that data collection for this second phase of the study occurred during the COVID-19 pandemic. Consequently, all essential measures to minimise the transmission risk between participants and the study team were adhered to according to WHO and national guidelines [20]. These included screening women for participation through temperature checks and excluding existing flu like symptoms, proper wearing of face masks throughout the data collection processes, hand hygiene, and physical distancing of at least one meter between individuals.

### Data analysis

The FGD and IDI recordings were transcribed verbatim. Focus group discussions and CHW transcripts were then translated into English by the first author who is fluent in both Ndebele and English. These were read several times to familiarise with the data, and cleaned to eliminate inconsistencies [21]. To bolster the credibility of the study, participants were asked to review the transcribed data to ensure its accuracy prior to its analysis. The first author independently coded the data; manually at first, then with the aid of Web ATLAS.ti software.

Focus group discussion and IDI transcripts were individually uploaded to the analysis software and thematic analysis applied using the inductive approach which allows the data to determine the themes. Open line-by-line data coding was applied to create labels that were attached to each segment of data that described the idea expressed. The coding system was organised using colours that provided a visual display of those that appeared frequently, and grouped related codes together. The codes were then examined to identify patterns among them which enabled the generation of themes.

The co-author whose role was academic supervisor to the first author and an experienced qualitative researcher provided oversight and checked the coding process. Furthermore, peer checking of the analysis process was done by another qualitative research expert not involved in the study, who reviewed the transcripts and assessed the credibility of the codes and extracted themes and sub-themes. Results are presented as direct quotes from the FGDs and IDIs based on the themes that emerged.

### Ethical considerations

All due ethical safeguards were observed before and during the entire research process. Ethical clearance for the study was obtained from the Health Research Ethics Committee of Stellenbosch University (Reference number S20/09/259) and the Medical Research Council of Zimbabwe (Reference number MRCZ/B/2426). Authority to conduct the study was granted by the Zimbabwe Ministry of Health and Child Care. Written informed consent was also obtained from the study participants. Anonymity of participants and confidentiality of information provided were assured. Participants were informed of the freedom to withdraw from participation at any stage of the study for whatever reason without suffering prejudice from the research team.

## Results

### Socio-demographic characteristics of participants

A total of 36 women participated in the FGDs out of 50 that had been recruited. Table 2 presents a summary of the participants’ socio-demographic characteristics. The age groups and screening statuses were evenly distributed across the sample. The larger proportion of participants had between one and four children (31:86.11%), were married (30:83.33%) and had attained a secondary level of education (21:58.33%).

**Table 2:** Socio-demographic characteristics of FGD participants.

Characteristics	Frequency	Percentage
**Age**		
25–34	13	36.11
35–44	12	33.33
45–50	11	30.56
**Parity**		
1–4	31	86.11
5^+^	5	13.89
**Marital status**		
Single	2	5.56
Married	30	83.33
Widowed	3	8.33
Divorced	1	2.78
**Educational attainment**		
Primary and below	13	36.11
Secondary	21	58.33
Tertiary	2	5.56
**Screening status**		
Screened	19	52.78
Not screened	17	47.22

Twenty-five IDIs were conducted with health providers from community level, primary health care level and the Provincial Hospital. None of the health providers approached declined to participate. A breakdown of the participants’ positions is given in Table 3.

**Table 3:** In-depth interview participants by work position.

Position	No. of participants
Doctors	2
Nurse Administrator (Matron)	1
Community Health Nurse	1
VIAC trained nurses	3
Non-VIAC trained nurses	11
Community Health Workers	7
**Total**	**25**

### Emerging themes

**Fig 1:**
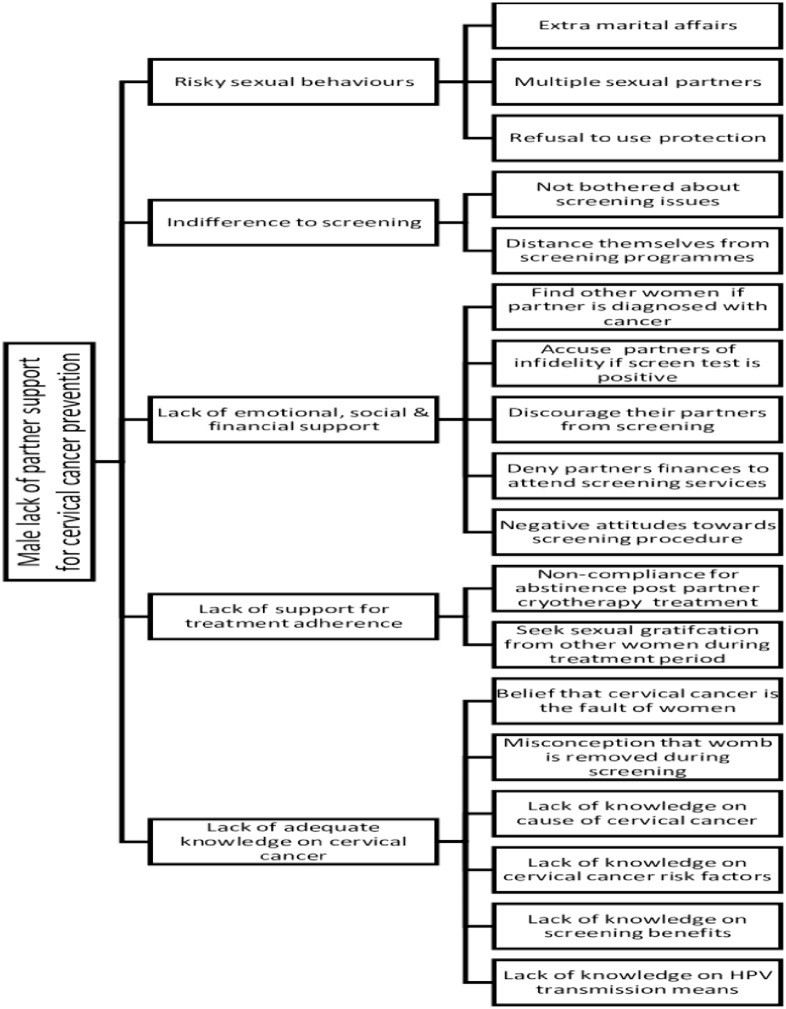
Description of the coding tree.

From the results, it emerged that male partners make a salient contribution towards increasing the incidence of cervical cancer. Men also present barriers to women’s utilisation of cervical cancer screening services. Major themes that emerged from the FGDs and IDIs were: 1) men’s risky sexual behaviours 2) indifference towards screening, 3) lack of emotional, social and financial support, and 4) lack of support for post screening treatment adherence, all of which are centered on lack of knowledge. The coding tree that reflects the major themes and sub-themes is described diagrammatically in Fig 1.

#### Risky sexual behaviours

Women generally viewed men as exhibiting high risk sexual behaviours that perpetuate the incidence of cervical cancer. Men reportedly have multiple sexual partners, a risk factor for development of the disease.

*“The problem is in men*. *To them it’s normal to have extra marital affairs*, *which means they can infect all their sexual partners if they pick the disease from one of them*. *That’s why so many women end up with cervical cancer”*.

*(FGD 2*, Participant 3, 45 years, 4 children, married, secondary education, screened)

*“Sleeping with many women leads to cervical cancer*. *Like some men have up to seven*. *So*, *they can pass the cancer cells from one woman to the next”*.

(FGD 5, Participant 7, 50 years, 6 children, single, secondary education, not screened)

*“I will not be knowing that my husband will leave home and go to have sex with my neighbour*. *From my neighbour he will move to another woman*. *At the end of the day*, *he will have slept with many of us exchanging us”*.

(FGD 3, Participant 4, 42 years, 3 children, married, secondary education, screened)

The patriarchal nature of decision making in sexual matters was seen as potentiating the problem:

*“Women have no power to protect themselves during sex*. *It’s men who have the power to say how they want it done and they don’t want to use condoms in marriage so they can bring it from their other sexual partners”*.

*(FGD 5*, Participant 5, 25 years, 2 children, married, primary school, not screened)

#### Men’s indifference towards screening

Although men may not prohibit their partners from attending cervical cancer screening services, their perception is that SRH issues are the concern of women and have nothing to do with them. This was reflected in the responses that women gave when asked on the role male partners played in reducing the incidence of cervical cancer.

*“Most men are not bothered one way or another*, *but I don’t think they refuse for their wives to be screened”*.

(CHW, rural community 3)

*“Men usually do not have problems with women attending health services*. *They don’t bother themselves with these things quite a lot*. *It’s only a few who will forbid their women to be screened”*.

(FGD 1, Participant 5, 50 years, 3 children, widowed, secondary education, screened)

#### Lack of emotional, social and financial support

Women and health providers indicated that despite women’s awareness of the need for cervical cancer screening, some would still opt out due to fear of the consequences that might follow from their male partners. This was more so if the test result was positive. Participants reiterated the need for heightened education of male partners to promote normalisation of cervical cancer screening.

*“Other women are afraid to test because they don’t know how the husband will react to a positive result*. *They fear that the husband will leave her*. *They [men] may take a second wife if the first gets cervical cancer*. *So*, *I suggest that men should also be involved in these lessons so that they also understand and encourage their wives*. *Find ways of involving men in these lessons when you teach women about this disease and the importance of screening”*.

*(FGD 3*, Participant 2, 29 years, 3 children, married, secondary education, screened)

“And it’s also difficult to tell my husband because he will say where did you get it from, and then I start problems for myself”

*(*FGD 1, Participant 1, 31 years, 3 children, married, secondary education, not screened)

*“I agree that men are a problem*. *They are very difficult to convince……*. *If you talk about cervical cancer screening*, *he will ask you why you want to go looking for diseases and won’t allow you to go for screening*. *They refuse and tell you that you can go if you want but if you are found to have the disease; it will be yours because you will have looked for it*. *Men can be very difficult*. *I also support the idea of male involvement because this can make everyone have an appreciation of the programme*. *This way*, *the number of women coming up for screening would increase”*.

*(FGD 4*, Participant 7, 43 years, 3 children, married, primary education, not screened)

Health providers concurred with women on men’s lack of support and the need for their involvement for improving acceptance of the programme.

*“There are many [women] who will say their husbands don’t allow them to be screened”*.

(Primary Care Nurse, Rural Health Center (RHC) 2)

“*The issue of male support is a challenge……we should not take for granted that women will come without the knowledge of their husbands because they may refuse to allow their wives to attend screening if they have not been informed*. *So*, *there is a need to also target men with information in their circles for better acceptance of the screening programme*. *If you target one side*, *you lose”*.

(Community Health Nurse)

*“Most of the women that I have seen come to the hospital alone……They actually don’t have enough support*, *because even when they come*, *I have never seen anyone come with their partner*. *They have to go through with this all alone”*.

(State Registered Nurse, Paediatric Ward)

Lack of financial control on the part of women was seen to pose additional barriers to screening because men are unwilling to provide funding for women to access services they have reservations on.

*“You see; if you go for screening*, *since you are already HIV positive*, *then you are told you also have cancer*, *it may create friction because your husband will say you brought the disease and it’s your problem*. *He won’t even give you the money to go to Gwanda”*.

(FGD 4, Participant 3, 26 years, 2 children, married, primary education, not screened)

*“Involving men when teaching us will solve the problem because they are the ones who give us money*. *So*, *if they are involved*, *they will freely give us the money to go to Gwanda for screening”*.

(FGD 5, Participant 3, 31 years, 3 children, married, primary education, screened)

Men’s attitudes towards the way the procedure is done compel them not to allow their partners to go for screening. This also hinges on the cultural norms that view it as taboo for a woman’s private parts to be accessed by anyone or ‘anything’ other than her husband except when giving birth.

*“Some men don’t like it to be done on their women*. *They don’t like it*. *You know the thing which they use for cervical cancer screening*. *Some men they don’t like it*. *The woman will tell you that my husband doesn’t want*. *They are jealous of the tools that are being used there”*.

(State Registered Nurse, OIC)

#### Lack of male partner support for treatment adherence post screening

Findings of the study suggest that men are unlikely to abstain from sexual relations during their partners’ treatment period and will satisfy their sexual needs elsewhere. In order to avoid their partners from seeking sex outside their relationships, women would rather not screen.

*“The other challenge is that if women are VIAC positive and are treated with cryotherapy*, *we encourage them to stay for six weeks without sex*. *Our men do not want to stay that long without sex and they are the ones who make decisions related to sex*, *so women will not come for that reason to avoid problems at home”*.

(VIAC nurse 1)

“*Other ladies are afraid to come for screening because they are afraid of their husbands*. *Let’s say the woman comes and screens and is found positive*, *then we need to involve the husband in the treatment whereby after treatment she is supposed to abstain from sex for six weeks*. *So that’s where the challenge is*. *So*, *the lady will prefer not to screen because she will say ‘no’ I will have challenges in my home as he will get it from somewhere”*.

(VIAC nurse 3)

#### Men’s lack of awareness and knowledge on cervical cancer

Although men’s knowledge on cervical cancer and screening was not directly solicited, responses provided by participants reflected overall lack of knowledge of the risk factors for the disease and the benefits of screening. The following statements from women infer that men’s actions or inactions in relation to spousal/partner support are based on inaccurate information.

*“…………men should also be involved so that they understand that having cervical cancer is not the fault of the woman*. *That way they would be more supportive to their partners”*.

*(FGD 4*, Participant 6, 37 years, 3 children, married, secondary education, not screened)

*“They [men] think that if you go for screening*, *your womb will be removed*. *They do not understand why screening is done”*.

*(FGD 5*, Participant 11, 43 years, 6 children, widowed, primary education, not screened)

*“I support the idea of involving men*. *If he understands what causes cervical cancer and what puts a woman in danger of getting the disease*, *he will understand better because our men do not know yet they are the ones with many sexual partners who bring home these diseases*. *If they understand why women should be screened*, *they will support us”*.

(FGD 5, Participant 2, 40 years, 10 children, married, primary education, not screened)

*“Men should also be involved in the education of women about cervical cancer screening*. *Maybe if they understand why it is done*, *they will encourage their partners to be screened*. *It is not very helpful to teach women alone*. *We should be taught together with our partners”*.

*(*FGD 5, Participant 11, 43 years, 6 children, widowed, primary education, not screened)

Health providers also attested that men may not have the knowledge on HPV and how it is transmitted.

“………. *so they [men]should be educated on the mode of transmission of the virus that is involved in the causation of cervical cancer because most do not know*.*”*

(Community Health Nurse)

#### Proposed facilitators to male involvement

Most women and health care providers were optimistic that with appropriate interventions, men could significantly contribute to women’s increased uptake of cervical cancer screening. Suggestions were presented on how male partners can be involved in addressing barriers to the access of screening, key of which is education. Although the general feeling was that men would not of their own free will seek information on cervical cancer screening, participants offered ideas on how male education could be effectively implemented.

*“The issue of education comes in whereby………the partners need to be educated too*. *And since we all know that it’s difficult for men to come to a clinic setup*, *maybe …*. *the people responsible for the screening programme can go out there to communities and invite people including their partners then they can be educated*. *I think that way we can actually sensitise a lot of people and that way we can also talk to a lot of partners rather than asking them to come to the hospital so that we educate them*. *I think we have to go to the community”*.

(State Registered Nurse, Paediatric Ward)

*“……male nurses should discuss with men wherever they meet about this issue of cervical cancer so that men might take it [cervical cancer screening] into consideration for their partners*. *Then it will be easier for them to support their spouses*. *That way we can improve acceptance”*.

(Midwife, RHC 1)

*“The other problem is men*, *especially the young husbands*. *I wish there could be lessons which combine both men and women so that after men have also been educated…… they will remind each other”*.

(Health Promoter (VCW), urban community 1)

Some women were however adamant that men would not be interested to attend the educational sessions.

*“Even if community meetings are called [to educate men on cervical cancer]*, *they will not attend*. *They will push women to the meetings like they normally do”*.

*(FGD 4*, Participant 5, 38 years, 1 child, married, secondary education, screened)

To address this problem, participants suggested implementing male-centered educational programmes that would integrate information on cervical cancer. This would enable men to have an appreciation of the drivers of HPV transmission and re-infection, and the importance of screening and early treatment to prevent precursor lesions progressing to cervical cancer. Being well informed could enhance men’s support for women’s decisions to take-up screening.

*“My suggestion is that there should be a programme which focuses on male cancers where men will also get the education more appropriate to them*. *This will arouse their interest since the programme will be directly affecting them*. *As they get these lessons*, *they will stop blaming women for bringing such diseases home and women will get more support for screening”*.

*(FGD 4*, Participant 5, 38 years, 1 child, married, secondary education, screened)

Evidence to active male participation through programmes that benefit them is seen from the well accepted VMMC programme that is offered at the provincial hospital and at primary health facilities through mobile clinics.

*“…………*. *[VMMC has a high uptake*, *but] we didn’t do like what those who were doing circumcision did*. *We didn’t do those mass pre-campaigns [for cervical cancer screening]*.*”*

(VIAC nurse 3)

While the focus of the study was on male involvement, it also emerged that community leaders have an influence on how communities accept health programmes and hence, are an effective vehicle in getting information through to their communities.

*“I would also like to suggest that if men are to be called to join women when we are being taught about cervical cancer*, *this be done through community leaders*. *Without doing that*, *men will never come*. *They are stubborn and don’t like to attend any meetings unless if it has to do with them directly”*.

(FGD 5, Participant 4, 34 years, 3 children, married, secondary education and screened)

*“The other issue is to intensify community mobilisation and involve men*. *We should not take these things for granted even without organising meetings*. *Find them where they are*. *Always involve community leaders*. *When information comes through their leaders*, *people will understand it better”*.

(Community Health Nurse)

An example was however highlighted on the facilitation role that male partners play in support of the screening programme. This could be an indication that with more knowledge, men can provide social and financial support for their partners to access screening services.

*“There are a few [men] though who when you put a message on the village WhatsApp group*, *they will even comment*, *meaning that if men were to be fully involved in this programme*, *we could see more women coming up for screening”*.

(CHW, rural community 3)

## Discussion

To the authors’ knowledge, this is the first exploratory analysis of male involvement in cervical cancer screening programmes in Zimbabwe. In-depth interviews and FGDs were used to assess the perspectives of health providers and women that participated in a two phased study on barriers to cervical cancer screening in the Gwanda district of Zimbabwe. The study identified men’s risky sexual behaviours, indifference towards cervical cancer screening, lack of male partner’s support for screening and treatment, and inadequate awareness and knowledge on cervical cancer as key areas that negatively impact cervical cancer screening. Participants identified means through which male partners serve as barriers to cervical cancer prevention and recommended potential strategies for male involvement in order to achieve a positive impact for the programme.

Castellsague et al. identified that men who have had many sexual partners place their current partners at risk of cervical cancer as they may be vectors of high risk HPV types [11]. From the mentioned study, it was observed that men maintain multiple sexual partners, a practice that increases the transmission of HPV. Consistent with this, the Zimbabwe Demographic and Health Survey of 2015 also revealed that more men than women reportedly had two or more sexual partners. The mean number of partners slightly increased as education and wealth increased, and only 37% of men reported condom usage at their last sex encounter [14]. It would seem that men are oblivious of the risk they add to increasing the incidence of cervical cancer among their partners. This is a cause for concern with implications for policy and practice. There is need to implement changes in SRH policies that would foster a collaborative approach to maintenance of family health that involves men. Social and Behaviour Change Communication could apply innovative educational interventions that utilise peer and community health educators. Such approaches could be more effective in changing the multiple partnering norm among men in addition to their education on cervical cancer.

This current study also identified that women and health providers perceived men’s knowledge pertaining to cervical cancer and screening to be low, with some men holding incorrect beliefs about screening. Considering that cervical cancer educational programmes have primarily targeted women with little attention paid to men [10], this finding is not unexpected. It would be unrealistic to expect men to know better since clearly, women also lack full understanding of the disease and its preventive screening measures as revealed in previous studies from Zimbabwe [22] and Gwanda district in particular [23]. This is further confirmed by the fact that women in this study were aware of the risk for developing cervical cancer posed by having a high risk sexual partner yet failed to demonstrate knowledge of HPV as the primary risk factor for the disease.

Findings are consistent with previous studies conducted in Kenya [9], Ghana [8], South Africa [10] and among Latino immigrant [24] and Sub-Saharan African immigrant men in the United States [25] that similarly found that men had inadequate or inaccurate knowledge on cervical cancer. However, men have expressed willingness to learn more to enhance support of their partners’ health seeking behaviours for screening [6, 8, 26] as was also inferred in this study. This finding could be capitalised on, and strategies developed for male targeted educational interventions on cervical cancer screening. These should be implemented at places where men are, using culturally tailored methods. Stakeholder engagement is therefore necessary for the successful development and implementation of these interventions.

Almost all women and health providers recommended education of men on all aspects of cervical cancer with the hope that an increase in knowledge among male partners could enable improved understanding of risk factors and screening and treatment processes. Similar suggestions were given in a Kenyan study by Adewumi et al. [2]. Findings of the current study that are consistent with a South African study by Maree et al [10] provide evidence for the need to apply innovativeness in the delivery of education on cervical cancer to males since it is unlikely that men will present themselves to health facilities to receive the education.

Another important finding that emerged from this study was the lack of, or inadequate emotional, social and financial support from male partners that limited women’s access to cervical cancer screening and treatment. This practice is countercultural for a generally very patriarchal society like Zimbabwe where men are expected to provide for their entire households [27]. Furthermore, men detached themselves from the whole process even when they did not prohibit their partners from accessing screening services. In a study conducted in one Metropolitan Province in Zimbabwe, Tapera et al [28] argues that socio-demographic inequities exist which determine the uptake of cervical cancer screening services. Moreover, factors engrained in the societal norms that relate to social support and networks could be a deterrent to male involvement in screening-related activities. Consistent with these findings, Gutusa and Roets [29] also identified that some Zimbabwean men forbid their wives to access the service due to lack of awareness on the benefits of screening.

These findings however differ from those of Adewumi et al. [2] and Binka et al. [6] conducted in Kenya and Ghana respectively. The mentioned studies found that men provided support to their partners in form of funds for transportation and accompanying them to screening facilities, encouragement for screening and adhering to sexual abstinence during treatment. Reasons for this discrepancy could be the lack of appreciation for cervical cancer prevention by Zimbabwean men. This could be due to low awareness and knowledge on the subject taking into account that the screening programme is currently not available at RHCs and mine clinics and is provided only to women. Awareness creation campaigns on cervical cancer and screening should be incorporated into all health programmes including those that are male focused.

Knowledge gaps have the potential of influencing men to unwittingly withhold the support necessary for women to seek screening and treatment services. This underscores the need for developing strategies that effectively involve male partners in cervical cancer prevention and control promotion programmes if they are to be readily acceptable within families and communities. The VMMC programme that attracts high volumes of men in the study district could be used as a platform to disseminate information on cervical cancer. These two programmes complement each other very well given that VMMC has a protective effect against cervical cancer [13].

The cruciality of involving community leaders to spearhead male involvement in cervical cancer prevention and control programmes as implied by both women and health providers is encouraging. It is best practice to recognise the community mobilisation role that civil society plays in the identification of challenges and development of strategies to overcome them as advocated by WHO [30]. This has the effect of facilitating successful uptake of programmes by all stakeholders at community level.

## Limitations

Findings of this study should be interpreted with consideration that data were collected during the second wave of the COVID-19 pandemic. Although maximum variation sampling had been applied in recruiting participants for FGDs, there was an imbalance in the sample in terms of marital status, parity and educational attainment. Some women who had agreed to participate did not turn up for the discussion, probably due to fear of exposing themselves to the risk of contracting the disease. The same problem affected the sizes of the groups that had been planned at ten participants but had an average of seven participants with the least having five. This could have biased the results in favour of married women who were over represented in all groups. We also acknowledge that the data is analysed from the perspective of health providers and female partners on the behaviour, beliefs and attitudes of their partners and that no data were collected directly from the male partners. Whereas the findings of the study may not be generalisable due to the qualitative approach that was used, they are probably true for most of Zimbabwe and other similar settings in the region.

## Conclusions

Findings of this study suggest that men have limited knowledge on cervical cancer in Gwanda district, Zimbabwe. Consequently, they practice risky sexual behaviours that increase the likelihood of their female sexual partners developing the disease. Our results also suggest that male partners serve as a barrier to cervical cancer screening seeking behaviours and treatment adherence through lack of partner encouragement and failure to provide financial, social and emotional support. Seemingly, men have not been adequately targeted in cervical cancer awareness programmes probably because it is not a men’s disease.

These findings underscore the overarching need of including males in cervical cancer education programmes as a critical component in the disease’s prevention and treatment. Interventions to achieve this goal need to be multifaceted, combining different approaches contextualised to the different categories of men in the district. These targeted interventions should focus on addressing men’s attitudes towards screening. Comprehensive education on the cause and risks factors of the disease, screening procedures and treatment methods could enhance male support during the screening and treatment processes. Leveraging on the existing public health system, men should be reached where they are including at their workplaces and homes, thus making the service readily available and accessible to them.

Increased knowledge could eliminate some barriers linked to male partners with the ultimate goal of increasing uptake of cervical cancer screening and reducing the burden of the disease. Needless to say, education of women should also be intensified as they also evidently still need better understanding of cervical cancer and the benefits of screening. Future male-focused studies should look at the knowledge and beliefs related to cervical cancer screening among men in Zimbabwe.

## Supporting information

S1 File Focus group discussion guide. (PDF)

S2 File Focus group discussion manual coding. (PDF)

S3 File FGDs quotations ATLAS.ti output. (XLSX)

S4 File FGDs codes ATLAS.ti output. (XLSX)

S5 File In-depth interview guide. (PDF)

S6 File In-depth interviews manual coding. (PDF)

S7 File IDIs quotations ATLAS.ti output. (XLSX)

S8 File IDIs Codes ATLAS.ti output. (XLSX)
