# Supplementary material for: Women and health providers’ perspectives on male support for cervical cancer screening in Gwanda district, Zimbabwe
Source: PLoS One. 2023 Oct 12;18(10):e0282931. doi: 10.1371/journal.pone.0282931 (PMC10569579; doi:10.1371/journal.pone.0282931)
Supplement: S1 File — (PDF) [file pone.0282931.s001.pdf]

FOCUS GROUP DISCUSSION GUIDE

Barriers to cervical cancer screening in Gwanda district, Zimbabwe: A mixed method analysis

Introduction

My name is ……….. a PhD student from …….. I would like us to discuss about cervical cancer and screening practices among women in your district. The purpose of the discussion is to learn more about your knowledge and beliefs on cervical cancer and practice of cervical cancer screening, and to identify difficulties that you as women face in accessing screening services. Your opinion is very important because it could help find ways to address those difficulties to improve the cervical cancer screening programme in the future.

Your participation is completely voluntary, and you can choose not to participate in part or at all in the project. You are free to keep quiet if you do not want to talk about any issue and you can withdraw at any stage without being penalised or disadvantaged in any way. Any information that you provide is strictly confidential. Your privacy will be protected, and full steps will be taken to ensure anonymity. Whatever you say will not be linked to your name so feel free to express your honest opinion. There is no right or wrong answer so please express whatever is in your mind and make suggestions. The discussion will take about one hour.

I would like to record the discussion session so that I do not lose any important information that will come up during the discussion. Please speak loudly so that we can hear the information you give us. I will transcribe all the information from the recorder and the recordings will be destroyed when it is no longer required. The recording will not be used for any other purpose other than this study and will not be accessible to anyone else other than the research team. You can verify your comments and responses after the recording.

Are there any questions you would like to ask? Thank you for your willingness to participate in this study and now we will get started.

FGD ID ____

FGD Location ____

FGD Date ____

Time started ____

Time completed ____

Definitions

During this focus group, we will be dealing with many technical and sometimes sensitive terms, can we just go through them, so we all know what we are talking about when we use certain words.

Probe for local name for cervical cancer

Probe for local names for the male and female reproductive organs

Difference between cervix, womb and vagina

Sexual acts ("sleeping together", oral, anal, hand, thigh, other)

Questions

1.What do you think is the most common cancer among women in Zimbabwe?

- Rank top 3
- Motivate responses

2. The number of women suffering from cervical cancer in Zimbabwe is on the increase. What can you tell me about cervical cancer?

- Which organ is affected?
- Knowledge of HPV as the primary cause
- Socio-cultural beliefs such as witchcraft, associated myths and misconceptions
- Who is most likely to develop cervical cancer?
- Probe for the most 'at risk' age group

3. Please could you name as many things as you can think of that could increase any woman's chances of getting cervical cancer?

- Probe for cervical cancer risk factors
- Prompt with ………anything else?
- Probe for specific factors that predispose women in the local community to cervical cancer - Probe for any socio-cultural practices

4. There are many warning signs of cervical cancer. Please could you name as many as you can think of?

- How confident are you that you would notice a cervical cancer symptom?
- Probe for health awareness programmes

5. What would you do if you had a symptom that you think could be cervical cancer?

Probes:

- Would you ignore it, would you try self-medication, would you tell someone close to you, would you visit a clinic or hospital, would you visit a traditional healer or is there anything else you would do?
- How soon would you take that action – visit clinic or hospital, traditional healer?

6. As far as you are aware, is there a vaccination to protect against cervical cancer? If yes, at what age is it offered?

7. Could you please suggest ways that could be used to detect cervical cancer in its early stages when it can still be treated?

o Known screening methods

8. As far as you are aware, what cervical cancer screening services are available in the district?

- VIAC Clinic, Outreach services, ZNFP private clinic
- At what age should a woman start screening?
- How often should a woman be screened
- How common is it for women in your community to have cervical screening?
- Could you please share your cervical cancer screening experiences?

9. Sometimes women put off going for cervical cancer screening, what could be the reasons for this?

Probe for:

- Socio-cultural and religious barriers to screening practices
  - How difficult is it to talk about sexually related issues among women in your community?
  - How best can issues on sexuality be discussed among women?
  - Role of men in the decision for screening: protection of partners from acquiring HPV, encouragement for screening, financial and psychological support
- Health system related barriers
  - Access to information
  - Availability of services
  - Access of services

- Community factors
  - Social stigma
  - Cultural and societal norms
  - Gender norms
  - Religion
- Interpersonal factors
  - Partner support
  - Family support
  - Peer pressure
  - Social networks
- Good practices of screening

10. What would you like to see done differently in relation to the cervical cancer screening programme in Gwanda district?

- Probe for strategies and recommendations for improving the screening programme

Thank you for taking time to answer my questions. Now that the discussion is over, are there any questions you would like to ask, or do you have any comments from our discussion?
