# Supplementary material for: Women and health providers’ perspectives on male support for cervical cancer screening in Gwanda district, Zimbabwe
Source: PLoS One. 2023 Oct 12;18(10):e0282931. doi: 10.1371/journal.pone.0282931 (PMC10569579; doi:10.1371/journal.pone.0282931)
Supplement: S2 File — (PDF) [file pone.0282931.s002.pdf]

FDG TRANSCRIPTIONS – BARRIERS TO CERVICAL CANCER SCREENING IN GWANDA DISTRICT

Addressing objectives 3& 5

Objective 3: To identify factors that women aged 25-50 years in Gwanda District perceive as barriers to cervical cancer screening

TRANSRIPT	CODE
**FGD 1**<br>**Interviewer:** Thank you ladies for taking part in this focus group discussion and for highlighting the acceptable and used terms for intimacy and reproductive health organs in this community. Can we start the discussion now that we are all on the same page? There are no wrong answers, so just be free to say out what you know or think. In your own views, which are the most common cancers affecting women in Zimbabwe? You can give me 3 types ranking them in order of their commonness.	
**Participant 4: 50 years 2 children, widowed, tertiary education, screened**<br>The commonest is cervical cancer followed by breast cancer.	Cervical cancer<br>breast cancer
**Participant 5: 50 years 3 children, widowed, secondary education, screened**<br>I agree with that. Cervical cancer is the deadly one we know. Once you catch it and delay going to hospital it spreads all over, what follows is death, then you leave your children to suffer.	Cervical cancer
**Interviewer:** Why is cervical cancer such a problem in Zimbabwe?	
**Participant 5: 50 years 3 children, widowed, secondary education, screened**<br>The reason is that when we were growing up, we were taught that we have to treat our vaginas with herbs to keep them sexually appropriate. These are the things which later cause cancer because they turn into poison.	Inserting herbs in vagina
**Participant 3: 30 years 2 children, married, secondary education, screened**<br>The other reason is the perfumed soaps which we use. When we use them in our private parts, they disturb the soft tissues there and then cancer of the cervix occurs. Breast cancer is the second most common but this one is better because it can be treated more successfully.	Use of perfumed soap in vagina
**Interviewer:** Anything else?	
**Participant 1: 31 years 3 children, married, secondary education, not screened**<br>Inserting fingers into the vagina when you have long nails while bathing can cause cervical cancer. The nails can scratch you then through that laceration the virus which causes cancer can get in.	Inserting fingers in vagina
**Participant 2: 29 years, 3 children, married, secondary education, not screened**<br>Cervical cancer is the most problematic cancer because some women cannot afford to buy pads. They use pieces of cloth and it is difficult	Cervical cancer<br>Poor menstrual hygiene

to change them often. So, when they stay a long-time, germs will move up to the cervix and cause cancer.	
**Interviewer:** What I am getting is that cervical cancer is the most common in Zimbabwe. When we look at the figures, the number of women with this disease keeps on increasing, what can you tell me about this cancer?	
**Participant 5: 50 years, 3 children, widowed, secondary education, screened**<br>This cancer is now very common and now we see it attacking any age. Even grandmothers, we hear that, that old lady has cancer of the cervix. Our request is that education on cervical cancer should be given to all women who seek whatever services in clinics and hospitals. Or print out information pamphlets which you should distribute to all people so that people read for themselves. Also display posters at all places where people can see. These should be in all languages not just in English because some people don't understand English. You should also group women as you have done today to teach them. There are two groups of women. Some still believe in traditional things and there are some churches which do not believe there is cancer and they use preventive measures such as drinking holy water and using enemas. They have no knowledge. This education should be spread in churches so that young women and the elders also learn about it and get to understand because this disease is dangerous, and it kills.	Affects any age<br>Lack of effective awareness and education programmes at health facilities<br>Lack of IEC materials<br>Display posters at strategic places<br>Use local languages for IEC materials<br>Intensify education on cervical cancer<br>Cultural beliefs<br>Religious beliefs<br>Lack of knowledge on cervical cancer<br>Use church as education platform to accommodate women of all age groups
**Participant 3: 30 years 2 children, married, secondary education, screened**<br>I would like to add there that women should be encouraged to be tested for cancer of the cervix. Education should be given to women because most of them know about this but are afraid that the instrument which is inserted down there is painful.	Myths and misconceptions<br>Intensify education<br>Women are aware of the screening programme
**Participant 1: 31 years 3 children, married, secondary education, not screened**<br>I have not been screened myself because those who have screened tell us it is very painful. So, I am afraid of that.	Myths and misconceptions
**Interviewer:** What else do you know about cervical cancer?	
**Participant 4: 50 years, 2 children, widowed, tertiary education: screened**<br>I think if your immunity is compromised like in HIV, chances of developing cervical cancer are very high. Sometimes the well-being of a person makes them easily affected.	HIV is a risk factor
**Interviewer:** Ok. Who is more at risk of developing cc?	
**Participant 4: 50 years 2 children, widowed, tertiary education, screened**<br>As long as you are sexually active, you have chances of getting cervical cancer because sexually activity increases the chances of having it.	All sexually active women are at risk
**Participant 5: 50 years, 3 children, widowed, secondary education, screened**	

Promiscuous women are more at risk and older people who still like having sex. It’s easy for them to get the disease, especially if you have partners who are younger than you.<br>**Interviewer:** Would you please elaborate on that.<br>**Participant 5: 50 years, 3 children, widowed, secondary education, screened**	Multiple sexual partners<br>Older women who still have sex are at risk
If you have sex with many people especially the younger ones, chances of you getting a sexually transmitted disease are higher and if you always suffer from sexually transmitted diseases, chances of getting cervical cancer also increase. And to comment on what others have said it’s about fearing pain. It’s that instrument; it’s painful when they insert it, maybe for me it’s because I have not had sex for 10 years since my husband died, but it’s not right to scare others who have not been screened because this is a lifesaving test, if you are screened and this disease is seen early, it can be treated, but if you delay you die.<br>**Interviewer:** Someone earlier mentioned that some churches which say they can treat Cervical cancer. What are their beliefs about cervical cancer?<br>**Participant 5: 50 years 3 children, widowed, secondary education, screened**	Multiple sexual partners<br>Sexually transmitted diseases<br>Procedure is painful<br>Screening detects disease early while curable
Many of these religious groups and people grounded in traditional beliefs say when you get cancer you would have been bewitched. They just say you were bewitched but the truth is you would have bewitched yourself by not testing. Some will tell you in such and such a year someone stole your pant and bewitched you. Then you believe them and start accepting their treatment instead of going to hospital. I don’t refuse that witchcraft is there but when it comes to things like cancer which can be treated in hospital, that’s where we should go.<br>**Participant 6: 38 years, 3 children, single, secondary education, not screened**	Cervical cancer is due to witchcraft<br>Witchcraft is there but still need to go to hospital
Others tell us that if you are spiritual enough, you do need to do all these things because God will protect you.<br>**Participant 4: 50 years,2 children, widowed, tertiary education, screened**	Religious beliefs
Culturally we understand cancer to be a disease which occurs because someone has bewitched you. It’s not a disease that can just start. This applies to all types of cancers be it in the lungs, hand or legs or where ever. So, what is needed is to first remove such beliefs in women before you can expect all women to accept screening. They have to understand that screening helps in the prevention of cervical cancer.<br>**Participant 2: 29 years 3 children, married, secondary education, not screened**	Cervical cancer is due to witchcraft<br>Remove such beliefs so that women can accept screening<br>Screening prevents CC
I think that the use of herbs in the vagina after delivery to bring back the muscles to their normal tone. This causes cervical cancer and the practice is very common among mothers.<br>**Participant 5: 50 years, 3 children, widowed, secondary education, screened**	Inserting herbs in vagina

These herbs are also used to tighten the vagina so that you become tight and that is nice to your sexual partner and they never leave you for other women. And also, to thoroughly wash inside the vagina to remove all the discharges is not good because God made us like that. That is what causes cancer.<br>**Interviewer:** We have heard about inserting herbs in the vagina and inserting fingers when bathing, could we move to the warning signs of cervical cancer unless if there are other risk factors for cervical cancer which have not been mentioned?<br>**Participant 1: 31 years 3 children, married, secondary education, not screened**<br>Having many sexual partners can also increase the risk of cervical cancer especially here in Gwanda. This is because of many gold panners who flash money around, you find some women changing men to get money from as many of them as possible.<br>**Interviewer:** Ok, now I would like you to tell me the signs which can make a woman suspect they have cervical cancer.<br>**Participant 5: 50 years, 3 children, widowed, secondary education, screened**<br>On the signs of cervical cancer, you now start bleeding non-stop, you go on a menstrual period continuously.<br>**Participant 4: 50 years, 2 children, widowed, tertiary education, screened**<br>You can also experience abdominal pains.<br>**Participant 3: 30 years 2 children, married, secondary education: screened**<br>I also hear that; isn't it all women have a whitish discharge which is normal, if that discharge becomes too much and starts smelling badly, then that could be a sign of cervical cancer. The discharge smells like something which is rotting.<br>**Interviewer:** How or where do you get information which help you know those things about cervical cancer?<br>**Participant 5: 50 years, 3 children, widowed, secondary education, screened**<br>When you go to the clinic, we are taught about this cancer of the cervix. But sometimes you may go to the clinic and be unfortunate that on that day there is no lesson on cervical cancer then you just get the information from others. But then you won't get all the details compared to when you hear the information yourself first hand. We also request that in churches; could we have people coming to address women on women's days which take place during the week because most women go to church. A nurse should come from the hospital to teach on cervical cancer. In some churches, women meet Wednesdays, others Thursdays and others Saturday. They should use such opportunities and go round the churches. Some women may be having the symptoms, but they don't know what it means. If a woman is among other women, they are free to ask when they hear others also asking about health issues. Women need to be taught that it	Inserting herbs in vagina<br>Inserting fingers in vagina<br><br>Women in Gwanda have multiple sexual partners<br>Gold panning influences promiscuity<br><br>Bleeding between periods<br><br>Abdominal pains<br><br>Unusual vaginal discharge<br>Foul smelling vaginal discharge<br><br>**Sources of information:**<br>Clinic<br>Other women, but information scanty<br>Most women attend church<br>Use churches as education platform<br>Lack of knowledge on cervical cancer<br>Myths and misconceptions<br>Lack of symptoms in early stages

doesn't matter even if you do not have a sexual partner like some of us who are widowed, you could have been left with the disease and it is only starting to show signs now. They say it takes years to show.<br>**Interviewer:** Are there any other warning signs you can think of?<br>**Participants:** silence<br>**Interviewer:** Ok. You have mentioned the signs which could be indicative of cervical cancer. Say you notice those signs; what would you think of doing or what action would you take?	**Action when you notice s & s of CC:**
**Participant 1: 31 years 3 children, married, secondary education, not screened**<br>As for me, if I notice these unusual things, I will go to the clinic immediately, but since they say cancer is twofold, I will also go to traditional healers. We hear they treat it better than in hospital. It's better not to take chances.<br>**Other Participants**: laugh	Go to clinic immediately<br>Also go to traditional healers
**Participant 3: 30 years 2 children, married, secondary education, screened**<br>In my view, since I have received a lot of education on cervical cancer, I would quickly go to the clinic to be examined and treated because I value my health and will take whatever treatment I am given.	Go to clinic immediately
**Participant 6: 38 years, 3 children, single, secondary education, not screened**<br>I would go to the clinic like I do with any illness. But since I know that my husband may also be sleeping around with other women, those women may bewitch me with cancer so that my husband leaves me for them, so I would also visit those traditional healers I know they fix these things for help.	Go to clinic immediately<br>Also go to traditional healers
**Participant 4: 50 years, 2 children, widowed, tertiary education, screened**<br>Depending on the gravity of the pain, chances are that I would go and see a doctor, but if the pain is not intense, I will take same painkillers first before I seek help from anyone because that's what I usually do. Before you think of going to queue, you start by self-treatment.<br>**Interviewer:** How soon would others take action if they notice that things are not well?	Self-treatment first
**Participant 5: 50 years, 3 children, widowed, secondary education, screened**<br>Like a clever woman who has been educated, and always reading about this disease in the newspapers, I would go to the clinic as soon as I notice the unusual things. Even if the nurse says please undress, I will do so because I want to be treated. Some women are reluctant to be screened because they hate the part of exposing their private parts but as for me, I think my health is too important to worry about that. Some of us have seen some women die of this cancer and I wouldn't want the same to happen to me if it can be helped. Even to join the queue I don't think I would queue. I would go in straight into the nurses' room.<br>**Other participants:** all laugh	Go to clinic immediately<br>Modesty issues<br>Know someone who has died of cervical cancer<br>Have seen women die of cervical cancer

**Participant 5: 50 years, 3 children, widowed, secondary education, screened**<br>Yes. This is a serious disease ladies. I am talking from experience because I have seen a relative die from this cancer of the cervix. They said she went to hospital too late.<br>**Participant 3: 30 years 2 children, married, secondary education, screened**<br>The other important thing is to listen to what the doctor tells you. Sometimes we know of some women who refuse to have their womb removed and they end up dying. There is still that belief that if your womb is removed you are no longer a full woman. Meanwhile they will leave the hospital and go to traditional healers. You have to listen to the doctor's advice.<br>**Participant 1: 31 years 3 children, married, secondary education, not screened**<br>My observation is that as women we always have that fear. Sometimes if I see that discharge and think of going to the clinic, I start thinking of what people will say, they may say I have a sexually transmitted disease and conclude that I am unfaithful to my husband. As women we still need a lot of education for us to fully understand about these things. We have heard about screening but there are still a lot of things we don't know. And it's also difficult to tell my husband because he will say where did you get it from, and then I start problems for myself. So, we really need to be taught so that we take care of our lives.<br>**Interviewer**: As far as you know, is there a vaccine which is available to prevent cervical cancer?<br>**Participant 5: 50 years, 3 children, widowed, secondary education, screened**<br>We have heard in the past 2 weeks; they were announcing with the hailer that girl children aged 5 years should be vaccinated against cervical cancer. It is my first time to hear that, I don't know about others. I even asked a nurse at the clinic why at such a young age and she said it's because children now start having sex at an early age.<br>**Participant 6: 38 years, 3 children, single, secondary education, not screened**<br>No, the vaccine is given to the grade 5 girls and not at 5 years. *She turns to the previous participant;* it's given to Grades 5: 9-14 years. It's called the HPV vaccine.<br>**Participant 5: 50 years, 3 children, widowed, secondary education, screened**<br>Ok. Thank you, so I had misunderstood.<br>**Interviewer:** Could you please suggest means which are available to detect cervical cancer in its early stages, when it is still treatable. You mentioned that at times women only go to the clinic too late when the cancer can no longer be treated. So, is there a way to detect it at earlier stages when it can still be treated?	Cancer is a serious disease<br>Have a relative who died from cancer<br><br>Cultural beliefs<br><br>Fear of stigma and discrimination<br>Intensify education on screening<br>Aware of screening but need full information<br>Lack of partner support<br><br>Inadequate knowledge about vaccine<br><br>Full knowledge about vaccine

**Participant 2: 29 years 3 children, married, secondary education, not screened**<br>VIAC screening can detect the disease early and it can be treated if you are screened on time if you delay, ah, then its death for you.<br>**Participant 4: 50 years, 2 children, widowed, tertiary education, screened**<br>I think the Ministry of Health should put in place systems, like with other child immunisations. We know, at what age these routine immunisations are given. There should be a similar system like that one and in the child's health card so that others know that the girl is due for this thing. It should be given at the clinic and then the clinic will check their registers and follow up if they haven't turned up. At schools yes, its fine but those who don't go to school are also most likely not to go to the clinic for that vaccine.<br>**Interviewer:** Which cervical cancer screening tests are you aware of?<br>**Participant 4: 50 years, 2 children, widowed, tertiary education, screened**<br>There is the Pap smear which was done when you go for the 6 weeks visits postnatal visit long back, but it is no longer done at the clinics. Private doctors still do it though.<br>**Participant 5: 50 years 3 children, widowed, secondary education, screened**<br>When I was screened, they inserted a metal instrument and I could see the picture of my private parts on a TV like screen.<br>**Interviewer:** That is what we refer to as VIAC, and it is the national screening method used in the public health facilities now.<br>**Participant 5: 50 years, 3 children, widowed, secondary education, screened**<br>What does that mean?<br>**Interviewer:** It stands for Visual Inspection with Acetic Acid and Cervicography. Acetic Acid is applied to the cervix and the abnormal cells in the cervix turn white and that shows when the pictures are taken.<br>Which places provide cervical cancer screening here in Gwanda district, and how common is it for women in your community to be screened?<br>**Participant 4: 50 years, 2 children, widowed, tertiary education, screened**<br>One can be screened either at our clinic here at Phakama or at Gwanda Provincial Hospital. The uptake is low and is influenced by the unfriendly environment at the clinic. You should go and see for yourself. You find very sick people lying on the ground waiting for long periods of time before they can be attended to. Then you ask yourself: should I be subjected to that treatment when I am not even sick. That's why most women don't go for screening. The nurse won't give priority to well women who have only come for screening when there are very sick people lying all over waiting to be attended to. And you want women to go there and spend hours waiting to be screened?	VIAC screening detects disease early and it can be treated<br><br>Inadequate knowledge on HPV vaccine<br><br>**Screening methods:**<br><br>Pap smear<br><br>VIAC<br><br>**Screening places:**<br><br>Phakama clinic<br>Gwanda Provincial Hospital<br>Screening uptake low<br>Non-user friendly clinic environment<br>Long waiting periods

**Participant 5: 50 years, 3 children, widowed, secondary education, screened**<br>Screening is done here and at the hospital. The thing which should be done at our clinic here is to have a separate queue for those coming for screening. Because like the previous participant has said, I cannot spend 6 hours in the queue just for screening. And you will be in the sun with nowhere to sit. That's why you find people sitting on the ground. Even when you want to get inside to drink water, they don't allow that because they will tell you you only come in by numbers. Maybe it's worse now because of Corona because they don't want everyone to come to the clinic, but unfortunately in the clinic even if there are many nurses, they are slow in attending to patients. We have a big problem. We have even brought that concern to the Ward Councillors. At one time we witnessed a child die while waiting. The mother had come as early as 07.00 hours but was still unattended hours later. So Aahh, it's not easy. We don't know whether the nurses are not enough, because some women queue and queue till they get discouraged and go back home before they can be screened.<br>**Interviewer**: Apart from Phakama and Gwanda Provincial Hospital, are there any other places which provide screening services?<br>**Participant 2: 29 years 3 children, married, secondary education, not screened**<br>Yes, there is a doctor who screens at his surgery but then money talks because there you have to pay for the service. We prefer to go to those places where the service is free. While the private doctors provide the service, most of us cannot afford the required fees.<br>**Participant 4: 50 years, 2 children, widowed, tertiary education, screened**<br>Once in a while, I think it's the Seventh Day Adventist Church where they come with nurses and doctors to do outreach clinic and they provide all services including screening. They take a week providing all health services.<br>**Interviewer:** According to your knowledge what is the recommended age when a woman should start screening, and how frequently should they be screened?<br>**Participant 5: 50 years, 3 children, widowed, secondary education, screened**<br>They say a person should be screened after 1 year. I think it should be more frequent say 6 months because if I am one who sleeps with many men, I can pick the infection before the year is up, and to wait that long, the cancer may be spreading.<br>**Interviewer:** I asked earlier about how common it is for women to be screened. Now we are recommending screening after every 6 months. How keen are the women in your community on screening?<br>**Participant 4: 50 years, 2 children, widowed, tertiary education, screened**<br>Women are not that keen, and not many have been screened. You know, it's a small community we stay in and the nature of the	Phakama Clinic<br>Gwanda Provincial Hospital<br>There should be separate queue for screening<br>Long waiting periods<br>Negative staff attitudes<br>Covid has decreased the number of people who can go to clinic<br>Child dying in queue<br>Women give up and go without screening<br>? Staff shortages<br><br>Private Doctor<br>Most cannot afford the fees<br><br>Periodically Health Expos by Religious group<br><br>**Screening frequency:**<br><br>Incorrect information on screening frequency

questions which are asked as part of the screening procedure are so personal such as: heh, when did you start to be sexually active, heh how many sexually partners have you had and so on. Why not just screen me and I go. So, women don't like to have their sexual history being picked on. Mothers prefer not to go, or if you go then you don't tell them the correct history and those are usually those who already have problems. For those with no symptoms to stand up and just go to be asked such questions is one of the reasons women don't go for screening. And the fact that we know those nurses at a personal level since they are our neighbours makes it hard. Imagine if I had slept with 7 boyfriends and I have to tell the nurse who is my neighbour, it's not really easy. It's a bit challenging.<br>**Other Participants:** Laugh and murmur, true, true.<br>**Interviewer:** So, the way I understand it is that women don't like to be screened because they are asked very personal information.<br>**Participants:** Yes	Screening rate low<br>Screening questions too personal<br>Give false sexual history<br>Difficult to disclose sexual history to nurses who we know at a personal level
**Participant 6: 38 years, 3 children, single, secondary education, not screened**<br>You see, the problem is you get there and the person who has to screen you is your neighbour. I will tell her that I have had 10 sexual partners. Before the end of the day ahh, the whole of Senondo township will be knowing, the nurses have no confidentiality generally, but sexual matters are even more sensitive. So, women will prefer not to go to the clinic than have their sexual history displayed as some nurses cannot keep your secret information to themselves. They broadcast it.	Screening questions too personal<br>Nurses lack confidentiality
**Interviewer:** Could you please share what your experiences was when you got screened?	**Screening experiences:**
**Participant 5: 50 years, 3 children, widowed, secondary education, screened**<br>I personally avoided screening at the clinic because my neighbours work there. I went to Gwanda Provincial Hospital instead and found 2 young male doctors. I requested not to be screened by them because as far as I am concerned, they are my children. So, I was screened by a female nurse. I told them that I was afraid of the metal instrument and she was gentle with me and I had no problem. Even when I went for rescreening this year after a year I requested to be screened by a female and they respected that. They said it's not a problem I could only see the doctor if I have a problem. I was referred to the doctor and he addressed me in Shona language which I do not fully understand. I told him I did not understand his language, so he then used the language I understand.	Professional<br>Gender of person screening<br>Use of unfamiliar language (negative staff attitudes)
**Participant 4: 50 years 2 children, widowed, tertiary education, screened**<br>I was screened in a private lab; I did not know the person and she did not know me. I however experienced some discomfort during the procedure, but I was treated in a professional way.	Treated professionally<br>Did not know the nurse<br>Experienced some discomfort

**Participant 3: 30 years 2 children, married, secondary education, screened**<br>I was screened at Phakama Clinic and was also treated well. I was screened by the nurse from OPHID who does not know me, and I did not have any problems.<br>**Interviewer:** Could there be some inconveniences which other women face when attending screening services?	Treated professionally<br>Did not know the nurse
**Participant 5: 50 years, 3 children, widowed, secondary education, screened**<br>Others have complained that the nurses are rough. You are told "open up your legs faster, I have a lot of work to do". I have heard about 3 women saying the nurses are rude and short tempered. We request nurses to be kind. The thing is you would have been told by other women that the procedure is painful, so you are bound to be hesitant when you climb onto that bed then you get shouted at. It discourages women. That's why the procedure becomes painful because if the nurse is angry, they will not be gentle when inserting that instrument. They want to move the queue fast.	Negative staff attitudes
**Participant 2: 29 years 3 children, married, secondary education, not screened**<br>I don't know if it's still the case now since Phakama is now also screening. But previously many women would be turned back when they went to Gwanda Hospital. If you don't get there very early, they will tell you their number for the day is enough and you have to come back some other day. Obviously, you won't have the energy or motivation to go back.<br>**Interviewer:** Ok. So, tell me, some women are aware that it is important to be screened, but still they do not go for screening, what could be the reasons for this? You have talked about the other factors, but I would like us to discuss more on this.	Limited number per day<br>Women turned away
**Participant 6: 38 years, 3 children, single, secondary education, not screened**<br>Some of us are afraid to get diseases. We are told that the instrument which is used is just washed and used on the next woman. So, I personally am scared of contracting diseases from other women from that instrument. That's why I have not gone for screening.	**Barriers to screening:**<br><br>Lack of confidence in hospital services
**Participant 1: 31 years 3 children, married, secondary education, not screened**<br>I am also afraid of the procedure. I once went and I was the third in the queue. When the first lady came out, she exclaimed that it's very painful and they had tried 2 instruments on her and she had suffered a lot of pain. So, I quietly left the queue and have since never gone back there.<br>**Other participants:** Laugh and some comment that yes those who have been screened discourage others.<br>**Participant 1: 31 years 3 children, married, secondary education, not screened**	Fear of pain during procedure

I even considered going to the Sister's Clinic because some say the treatment is much better there than here. The nurses there treat their clients well.<br>**Interviewer:** What is this Sister's Clinic.<br>**Participant 5: 50 years, 3 children, widowed, secondary education, screened**<br>It is a clinic which provides health services by the Red Cross to commercial sex workers, but I am not sure if they screen there. They attend to these 'sisters' but they don't turn other people away if you go there.<br>**Interviewer:** Anywhere else?<br>**Participant 2: 29 years 3 children, married, secondary education, not screened**<br>Here in our community at Senondo Township we speak freely about sexual and reproductive health matters. But there are also many commercial sex workers in our community. Since cancer of the cervix is associated with sexual activity, if you are seen going there for screening, other people will say "ah, so this one also changes men, they will label you." So, some of us are afraid of being wrongly labelled if you are seen in that queue for screening.<br>**Participant 1: 31 years 3 children, married, secondary education, not screened**<br>Some women are reluctant that you may go there and find it's a male who has to screen you. Some would have previously proposed love to you, and you rejected them or even if you don't know them, to expose your private parts is very difficult. We have no choice during delivery, but when you do not have any pain anywhere, it's really not comfortable to just open your legs to someone.<br>**Interviewer:** Someone mentioned that you wait long periods before you are attended to. Would you like to elaborate more on that?<br>**Participant 5: 50 years, 3 children, widowed, secondary education, screened**<br>No, once you get into the correct queue, the service for screening is very fast. The challenge is the first queue before you get into that one for screening. You have to queue for long periods at the gate before you are finally able to get in. But once your card is stamped and they screen the people according to the service you have come for; it does not take long at the screening room.<br>**Interviewer:** What role do men play in women's decision making for screening?<br>**Participant 5: 50 years, 3 children, widowed, secondary education, screened**<br>Men usually do not have problems with women attending health services. They don't bother themselves with these things quite a lot. It's only a few who will forbid their women to be screened.<br>**Interviewer**: Could there be other barriers to screening?<br>**Participants:** Silence	Negative staff attitudes<br><br>Not sure if Sisters clinic provides screening<br><br>Free communication on sexual matters<br>Many sexually workers in the community<br>Fear of stigma and discrimination<br><br>Gender of person screening<br>Modesty issues<br><br>No long waiting after registration<br><br>Men usually not bothered about women's health issues

**Interviewer:** We have covered a lot of barriers. We talked about knowledge and you recommended that awareness and education of women should be increased. We also talked about the negative stories by women who have been screened which discourage others. You also mentioned that it is not comfortable to be examined by males. You also mentioned lack of confidentiality on the part of some nurses. Given all these highlighted challenges, what else would you like to see being done in a different way that would enable more women to be screened? We are now talking about recommendations which you feel would improve the uptake of screening if implemented.	**Recommendations to increase screening rates:**
**Participant 4: 50 years, 2 children, widowed, tertiary education, screened**<br>I would suggest that we also have mobile screening teams from other areas. That way its people who don't know you, who will come screen you and go. So, you are free to tell them everything knowing it ends there because they don't know you.	Introduce Mobile clinics
**Participant 1: 31 years, 3 children, married, secondary education, not screened**<br>It would also help to have older mature nurses do the screening, not the young ones who gossip about what you tell them. All nurses should be taught to respect clients' privacy. That way women would be more comfortable to come.	Second mature nurses for screening<br>Nurses to respects client's privacy
**Participant 3: 30 years 2 children, married, secondary education, screened**<br>The questions which are asked during screening sort of violate someone's privacy. Honestly most answers you get there are not the truth. I would recommend that those questions are reviewed and made more user friendly. They sort of give the implication that cervical cancer can only occur in women who are promiscuous.	Review screening questions to be not too intrusive
**Participant 2: 29 years, 3 children, married, secondary education, not screened.**<br>Let's have a screening test which does not involve poking women's private parts. That is very uncomfortable. If maybe the test could be done in a different way which is more acceptable, more women would be willing to be screened.	Different mode of screening which does not involve exposure of private parts
**Participant 4: 50 years, 2 children, widowed, tertiary education, screened**<br>To address the knowledge gap, I will emphasise on what has already been said. We know that in Zimbabwe 95% of people go to church, so if the Ministry of Health uses the platform of churches, they would have reached 95% of people with information. I am aware that there are financial constraints, so nurses from different churches could be given slots once in a while to deliver some health education on the subject. Congregations with no trained nurses should be identified then nurses sent there periodically.<br>**Interviewer:** Is there anything else you would want to comment on?<br>**Participants:** Shake their heads.	Use churches as education platform

**Interviewer:** Thank you very much for the information and for the time you took to attend this focus group discussion. I trust the information will be useful in improving screening services in Gwanda.	
**FGD 2**<br>**Interviewer:** Welcome to this focus group discussion and thank you for your willingness to participate in this study where we continue to determine the barriers women face in accessing cervical cancer screening services in this district. Now I am also familiar with the terms which you use for reproductive and sexual organs and intimacy so we at the same level of understanding. Please tell me what you think are the top three most common cancers among women in Zimbabwe.<br>**Participant 3: 45 years, 4 children, married, secondary education, screened**<br>The most common cancer which affects women is cancer of the cervix which affects the mouth to the womb, followed by breast cancer which affects the breasts.	Cervical cancer<br>Breast cancer
**Participant 1: 37 years, 3 children, married, secondary education, not screened**<br>I also know that cancer of the cervix is the most common followed by breast cancer then cancer of the stomach.<br>**Interviewer:** Why do you think cervical cancer is so common in Zimbabwe?	Cervical cancer<br>Breast cancer,<br>Cancer of the stomach
**Participant 3: 45 years, 4 children, married, secondary education, screened**<br>The problem is in men. To them its normal to have extra marital affairs which means they can infect all their sexual partners if they pick the disease from one of them. That's why so many women end up with cervical cancer.	Partner with multiple sexual partners
**Participant 4: 25 years, 1 child, single, primary education, not screened**<br>The other reason why there is a high number of women with cervical cancer is because there are many people who have sexually transmitted diseases and here in this town there are many commercial sex workers. If you always get infected with a sexually transmitted disease, you can also easily suffer from cervical because both are transmitted sexually. People usually seek treatment for sexually transmitted diseases very late because men are the ones who usually infect us and may himself get treated but not tell his wife. If the woman notices the signs and symptoms, she will keep quiet for fear of being accused that she brought the infection to the home. The more the infection stays without being treated, the higher the chances of getting cervical cancer.<br>**Participant 1: 37 years, 3 children, married, secondary education, not screened**	Sexually transmitted diseases<br>Many CSWs<br>Both STIs and cervical cancer are sexually transmitted<br>Late treatment for STIs<br>Lack of partner support

Women also like inserting some powders made from herbs into their private parts to tighten the vagina which men find pleasurable during sex. This causes some irritation and burns the cervix which can result in cervical cancer in the long run. As you are wearing that mixture, you can feel some sort of pricking sensation there, so it means the herbs will be corroding the cervix.<br>**Participant 2: 43 years, 5 children, married, secondary education, screened**<br>Women believe they haven't properly cleaned their private parts if they have not inserted their fingers and thoroughly removed all the vaginal discharge in there. In the process of cleansing the vagina they may inflict lacerations to the cervix which will eventually result in cancer. That's what most girls are taught, to thoroughly clean themselves down there from the times they start bathing themselves. Some people also wash their private parts with perfumed soap. We have been taught that some ingredients in the soap may be too harsh to the cervix and repeated use over time could cause cancer.<br>**Interviewer:** Ok. It has been noted that the number of women who suffer from cervical cancer in Zimbabwe is increasing. What can you tell me about cervical cancer?<br>**Participant 5: 29 years, 3 children, married, primary education, screened**<br>Cancer of the cervix is now very common because girls start engaging in sex when they are still very young. The vaginal tissues will still be immature for that type of thing and is easily damaged. This puts them at a higher risk for cervical cancer because the tissues will be torn repeatedly, and cancer then develops from those wounds.<br><br>**Participant 2: 43 years, 5 children, married, secondary education, screened**<br>Elderly women are to blame for the high rate of cervical cancer because they are the ones who give younger women herbs to insert into the vagina. The belief is that men appreciate tight women more and women have been socialised into pleasing their men in bed, otherwise he will leave you for better performing women.<br>**Participant 1: 37 years, 3 children, married, secondary education, not screened**<br>I know that cancer of the cervix starts at the mouth the womb and if not treated on time, may spread to the womb itself and eventually it cannot be treated. That's why we are always advised to screen so that they catch the disease while it is still curable.<br>**Interviewer:** What other factors increase the likelihood of a woman developing cervical cancer?<br>**Participant 4: 25 years, 1 child, single, primary education, not screened**<br>They say smoking and taking alcohol increases the risk of cervical cancer, but I don't know if it's true?	Inserting herbs in vagina<br><br>Inserting fingers in vagina<br>Girls taught to clean using fingers<br>Use of perfumed soap in vagina<br><br>Early sexual debut<br><br>Socio- cultural practices<br>Elderly ladies socialise younger women into inserting herbs in vagina<br><br>Cervical cancer starts at the mouth of the womb<br>Screening detects the disease while it's still curable<br><br>Smoking<br>Drinking alcohol

**Interviewer:** That's true. Anything else? …..I see you have nothing more to add so we move to the next question. What are the warning signs of cervical cancer? Give me as many as you can think of.<br>**Participant 3: 45 years, 4 children, married, secondary education, screened**<br>Others say you can tell that you have cervical cancer if you start having a vaginal discharge which is smelling.<br>**Participant 5: 29 years, 3 children, married, primary education, screened**<br>This discharge is not the usual creamish colour and it becomes too much.<br>**Participant 1: 37 years, 3 children, married, secondary education, not screened**<br>The other sign of cancer of the cervix is that you start to bleed most of the time even when you are not due for your monthly period.<br>**Interviewer:** Anything else?<br>**Participants:** Silence<br>**Interviewer:** By what means do you get information about cervical cancer, and are you confident with the knowledge you have to be able to easily notice the signs and symptoms of the disease?<br>**Participant 1: 37 years, 3 children, married, secondary education, not screened**<br>I would say we are not so confident because some of the things we don't know. The best a woman can do for herself is to go for screening where the nurses will easily identify if there is anything not right.<br>**Participant 5: 29 years, 3 children, married, primary education, screened**<br>What I have realised is that as women we mislead each other when we talk about these things. Others say the instrument which they insert during screening stretches the birth passage and after that you will always have pain in your private parts. But that is not true.<br>**Participant 1: 37 years, 3 children, married, secondary education, not screened**<br>People do not want to be screened because they are afraid, they will be told they have cancer. This is very frustrating to hear and causes stress knowing that you are suffering from a disease which has no cure. I personally have not been screened and to me it's better that way because if I don't know, then I will not be subjected to stress. Stress can kill you sooner than the cancer. It is very easy to go for treatment for a condition you know about but looking for a disease which has not shown itself is not easy. As for lessons on cervical cancer, we get plenty of them, but people are afraid to be screened. These lessons are given at the clinic especially in Maternity. When you get to the clinic, the first thing they do is give health talks on many different health issues including cervical cancer.<br>**Participant 4: 25 years, 1 child, single, primary education, not screened**	Foul smelling vaginal discharge<br>Unusual vaginal discharge<br>Bleeding between periods<br>Lack of adequate knowledge on cervical cancer<br>Important to be screened<br>Myths and misconceptions<br>Fear of a cancer diagnosis<br>**Sources of information:**<br>Clinic gives plenty of lessons

Women do get the education on cervical cancer at the clinic, but we are overwhelmed with fear from information we get from the women who have been screened.<br>**Interviewer:** You have mentioned that health education on cervical cancer is given at the clinic. How else is information disseminated especially when considering those women who do not regularly visit the clinic?<br>**Participant 1: 37 years, 3 children, married, secondary education, not screened**<br>At times community meetings are called where education on cervical cancer can also be included by the Health Promoters. That is; information on what causes it, the signs and symptoms and how it is treated. They also encourage us to be screened. We as women are the ones who fail to take up the education and get screened. But as for information on that, we really get it.<br>**Interviewer:** Ok. So, after noticing some signs or symptoms which you suspect could indicate cervical cancer, what action would you take?<br>**Participant 1: 37 years, 3 children, married, secondary education, not screened**<br>The first option would be to go to the clinic immediately.<br>**Participant 3: 45 years, 4 children, married, secondary education, screened**<br>Others would go to these churches which prophesy or to traditional healers because they always think they will have been bewitched if they get any illness. The problem with us African people is that we put so much importance on issues of witchcraft.<br>**Participant 1: 37 years, 3 children, married, secondary education, not screened**<br>I get worried if I see unusual things happening in my body. So, I always do as they teach us that one should go to the clinic without delay if you feel unwell.<br>**Interviewer:** Could there be anything else others who have not contributed an answer to this question would do?<br>**Participant 4: 25 years, 1 child, single, primary education, not screened**<br>Other women go to the clinic first, then only resort to traditional healers if the problem continues.<br>**Interviewer:** I hear you. According to your knowledge, is there a vaccination which is available for the prevention of cervical cancer?<br>**Participant 2: 43 years, 5 children, married, secondary education, screened**<br>Recently we have seen girls being vaccinated against cervical cancer at schools. There has also recently been an announcement that they will be vaccinated again soon. But we are not aware of a vaccine which is given to adults.<br>**Participant 1: 37 years, 3 children, married, secondary education, not screened**	Clinic<br>Fear of screening<br>Myths and misconceptions<br>Health Promoters<br>**Action when you notice s& s of cervical cancer:**<br>Go to clinic immediately<br>Go to prophets<br>Go to traditional healers<br>Belief that cervical cancer is caused by witchcraft<br>Go to clinic immediately<br>Go to clinic first then traditional healers if problem continues<br>Inadequate knowledge of HPV vaccine

I don't know of any vaccine which protects against cervical cancer but what I know is that women should be screened so that they are treated on time and cervical cancer prevented.<br>**Participant 3: 45 years, 4 children, married, secondary education, screened**<br>The other prevention is to look after yourself. As I mentioned earlier, some women have many sexual partners and the same applies to men. This practice promotes the spread of the disease as it gets passed on to many women.<br>**Interviewer:** Would you know the name of the vaccine, and at what age it is given?<br>**Participant 5: 29 years, 3 children, married, primary education, screened**<br>It is given from Grade 5. I think they will be 8 years old.<br>**Participant 2: 43 years, 5 children, married, secondary education, screened**<br>No, at 8 years they are still in Grade 2. But yes, it starts from grade 5 up to Form 3 or Form 4 I think.<br>**Participant 3: 45 years, 4 children, married, secondary education, screened**<br>Its 10 years upwards.<br>**Interviewer:** Can I please clarify on this one. The vaccine is called HPV and is given to school going girls aged 10-14 years here in Zimbabwe. This is the age at which it is considered that girls would not have started engaging in sexual activities. The concept is the same as immunisations which are given to babies. The vaccine is given before the girls start sexual activity so that should they be exposed to the human papilloma virus later in life when they are now sexually active, their risk of developing cervical cancer is reduced. Please seek for more information on this vaccine from your clinic because it is important that you understand more about it. Your daughters need to be vaccinated.<br>You have continuously referred to screening as we were talking. Which screening methods do you know and what are the screening procedures?<br>**Participant 3: 45 years, 4 children, married, secondary education, screened**<br>There is this one where they use antiseptic acid. They put this antiseptic acid on the cervix, and you can also see your cervix on the screen.<br>**Interviewer:** Ok. That is called VIAC. Visual inspection of the cervix using acetic acid and Cervicography.<br><br>**Participant 2: 43 years, 5 children, married, secondary education, screened**<br>There is this other one where they scrap the cervix and send the specimen to the laboratory and you are told to come check for your	No knowledge of HPV vaccine<br><br>Avoid multiple sexual partners<br><br>Inadequate knowledge of HPV vaccine<br><br>Inadequate knowledge on HPV vaccine<br><br>Inadequate knowledge on HPV vaccine<br><br>**Screening method:**<br><br>Correct description of VIAC<br><br>Correct description of Pap Smear

results after some time. That’s how I was screened but I did not ask the name of the test.	
**Interviewer:** That should be the Pap smear.<br>So which facilities or Organisations offer cervical cancer screening services here in Gwanda district?	**Screening places:**
**Participant 5: 29 years, 3 children, married, primary education, screened**<br>Here at Phakama clinic and also at the big hospital.	Phakama clinic<br>Gwanda Provincial Hospital
**Interviewer:** Is there anywhere else where the services are provided?	
**Participants:** Silence.	
**Interviewer:** At what stage should a woman start screening and how frequently should they be screened?	**Screening frequency:**
**Participant 3: 45 years, 4 children, married, secondary education, screened**<br>A woman should be screened every year, but I don’t know at what age screening should be started.	Incorrect information on screening frequency
**Participant 2: 43 years, 5 children, married, secondary education, screened**<br>Long back it used to be every 5 years that a woman should be screened. You would be screened today and then told to come back after 5 years. But now I think it’s every year. Maybe they realised that there were many women now suffering from cervical cancer.	Incorrect information on screening frequency
**Participant 2: 43 years, 5 children, married, secondary education, screened**<br>Which screening method are we talking about here?	
**Participant 2: 43 years, 5 children, married, secondary education, screened**<br>The VIAC.	VIAC
**Participant 2: 43 years, 5 children, married, secondary education, screened**<br>In your own assessment, how common is it for women in your community to have cervical cancer screening?	
**Participant 4: 25 years, 1 child, single, primary education, not screened**<br>As it has been said earlier, people are afraid to be screened. Yes, some have been screened but many fear that instrument which is inserted because it stetches your vagina and men don’t like a hollow organ when they have sex.	Few screened<br>Women fear to be screened<br>Myths and misconceptions
**Participant 3: 45 years, 4 children, married, secondary education, screened**<br>Yes, women mislead each other and will exclaim about the size of that instrument. This results in other women getting scared of sustaining injuries during the procedure. That’s why many women have not been screened.	Few screened<br>Lack of confidence in health services
**Participant 1: 37 years, 3 children, married, secondary education, not screened**<br>I think it is very important that education on screening be intensified to get more women screened. As of now, very few women can tell	Intensify education on screening

you that they have been screened. At one time my neighbour was saying she has an unusual vaginal discharge. I advised her to go for screening yet I myself have never been screened because I fear the pain I will experience when they perform the procedure.	Few women screened<br>Procedure painful
**Interviewer:** That's interesting. Could we hear the screening experiences of those that have been screened?	**Screening experiences:**
**Participant 3: 45 years, 4 children, married, secondary education, screened**<br>My experience was very pleasant. These people know that women are afraid, so they chat with you throughout the procedure so that you relax. The environment was so relaxing that it made me feel that I had taken a wise decision by screening.	Treated professionally
**Participant 2: 43 years, 5 children, married, secondary education, screened**<br>I also did not have any challenges when I was screened, and it was not painful when they inserted the instrument. Even if they can say we need to be screened every month I would still go running.	Treated professionally
**Interviewer:** What then could be the reasons why most women have not been screened in addition to what you have already mentioned?	
**Participant 1: 37 years, 3 children, married, secondary education, not screened**<br>Screening is performed when a woman is well. So, can you imagine walking in for screening then being told that you have cervical cancer. That is very stressful because you can become ill just from knowing that you have cervical cancer and will soon die. That is another reason why women choose not to be screened.	**Barriers to screening:**<br><br>Fear of a cancer diagnosis<br>Fatalistic view of cervical cancer
**Participant 2: 43 years, 5 children, married, secondary education, screened**<br>The other big challenge is exposing your private parts. You hear women saying that they have no choice when they are in labour and delivering but when you are not sick, it is difficult to just open up your legs for the nurses unnecessarily. That's the other major thing which women are not comfortable with. Maybe if there could be a different way of screening, the uptake would be much higher than it is now.	Modesty issues<br>Recommend different way of screening
**Interviewer:** Could there be any other reasons? Let's hear from everyone.	
**Participant 4: 25 years, 1 child, single, primary education, not screened**<br>I have not been screened myself, so I don't know what happens in that department and who does the screening there. But when you are sick and you go to the clinic, there are some known nurses who you want to avoid because they go around talking about your illness. So, then I think if the same thing happens at the screening unit, it means everyone will be told everything about my private parts. Nurses should learn not to preach about their patients' illnesses. It discourages us from going to screen if you think of that, but I am not saying that is what is happening at the screening department. I am just saying.	Nurses lack confidentiality

**Participant 3: 45 years, 4 children, married, secondary education, screened**<br>Up to now, there are some people who still do not know their HIV status. These are the same people who are also afraid to be screened for cervical cancer. They fear that they may be screened for both and if found positive, family members and other people in the community will not want to associate with them.<br>**Interviewer:** Is being HIV positive such a big issue that people still discriminate each other on those grounds?<br>**Participant 3: 45 years, 4 children, married, secondary education, screened**<br>Yes, it still happens with other people.<br>**Interviewer:** How easy is it for women in your community to discuss about sexually related issues?<br>**Participant 4: 25 years, 1 child, single, primary education, not screened**<br>It is not a usual thing to talk about these things. It only happens when we are having lessons together as women on reproductive health matters. That is when we get the freedom to discuss sex related issues, not generally.<br>**Interviewer:** Any other views? Ok, nothing.<br>We have discussed at length about the barriers which women face in relation to cervical cancer screening. Now I want us to talk about what you would recommend improving access of the screening services by most women.<br>**Participant 1: 37 years, 3 children, married, secondary education, not screened**<br>Workshops should be organised where women are taught about the disease and the benefits of screening in more detail. I think this would motivate more women for screening. Full information would be delivered because the workshop would be dedicated just to that. Most women who have not been screened rely on what other people who may also not be well informed tell them. But if they were to get more knowledge to clear all their fears, I am sure they could accept to be screened. Even in churches, there are days when women meet on their own on Thursdays. Pastors should ensure that women's programmes include lessons on cervical cancer screening.<br>**Interviewer:** Could there be more ways of reaching more women with information which could also correct the wrong information which is circulating about screening?<br><br>**Participant 5: 29 years, 3 children, married, primary education, screened**<br>Education is the most important thing. It would also be better if women could be shown a person who has cervical cancer. I don't know if that is possible. I am sure if that is done no woman would want to remain unscreened because most people believe by seeing. If you just talk, to many it sounds like a fairy tale. Unfortunately, the	Fear of being tested for HIV<br>Fear of stigma and discrimination<br><br>No open discussions about sex related issues in the community<br><br>**Recommendations to increase screening rates:**<br><br>Organise workshops where women will get full information<br>Use churches as education platform<br>Involve Pastors in programming of cervical cancer education<br><br>Intensify education<br>Women to be shown someone who has cervical cancer

area affected is too private maybe it would be too hard on the affected person. But it's true that people are shaken into action when they see for themselves someone who is suffering from the disease. It will make them realise they don't want to get there.<br>**Interviewer:** How best could those workshops which were mentioned be organised so as to cover all women?<br>**Participant 1: 37 years, 3 children, married, secondary education, not screened**<br>Workshops will work well so that we have time to ask all the questions we have. These could be organised by Ward to have small numbers to teach at a time. The workshops should be continued till all Wards are covered and everyone understands the importance of screening.<br>**Participant 5: 29 years, 3 children, married, primary education, screened**<br>Let's also not forget that even if workshops are organised, some women will still choose not to attend. I personally think that it is better for Health Promoters to go door to door teaching about this, till all houses in their Wards have been covered. This way, it can be assured that the information has reached everyone.<br>**Participant 4: 25 years, 1 child, single, primary education, not screened**<br>In general, nurses should also stop the habit of discussing people's illnesses. That way, more would have the confidence to go for screening.<br>**Interviewer:** Anything else<br>**Participants:** Remain silent and some indicate by shaking their heads that they have nothing more to say.<br>**Interviewer:** Thank you so much ladies for your participation. It was nice hearing your views which could contribute to improving the cervical cancer screening programme in your district.<br>**Participants:** Thank you.	Organise workshops in each ward<br><br>Door to door education by Health Promoters<br><br>Nurses to behave in a professional manner
**FGD 3**<br>**Interviewer:** Good afternoon ladies. Thank You for attending this Focus Group Discussion. I am aware you had to attend a funeral this morning, but you still managed to come for the discussion. I would like to pursue further the responses which you gave during last year's survey so that I get a better understanding of the barriers which women face in accessing cervical cancer screening realising the uptake of screening in the district is much lower than expected. Could we please discuss the most common concerns afflicting women in Zimbabwe? You can just give me the top 3 according to what you know or think.<br>**Participant 4: 42 years, 3 children, married, secondary education screened**<br>The most troublesome cancer is that of the cervix. When one has this cancer, they always experience abdominal pains, sometimes one has a watery discharge or bleeding all the time. When you go to hospital many times you will be told that the cervix is now beyond treatment	Cervical cancer<br>S&S: abdominal pains, watery discharge and vaginal bleeding

and they end up removing it. The second that I know is breast cancer. It starts as a lump in the breast and you may ignore it thinking that it's a common boil. If you don't go to hospital, it will eventually burst, and this wound will never heal as it is now a cancer. The third one is cancer of the skin. If you get scratched in any part of the body, it becomes a wound which gets infected and never heals. Sometimes your end up losing limbs or any part of the body.<br>**Interviewer:** Anyone who has a different opinion or wants to add?<br>**Participants:** No<br>**Interviewer:** Cervical cancer cases are continuously increasing in Zimbabwe. What do you know about cervical cancer? You can tell me anything you heard about cervical cancer.	Breast cancer where you<br>S&s: breast lumps<br><br>Cancer of the skin<br>S&S: Wounds which never heal
**Participant 2: 29 years, 3 children, married, secondary education, screened**<br>This cancer is caused by inserting herbs in the vagina or having sex when you are still young, married to a person who sleeps with many other people and if you have suffered from a sexually transmitted disease, you are most likely to get the disease.	Inserting herbs in vagina<br>Early sexual debut<br>Partner who has multiple sexual partners<br>Sexually transmitted diseases
**Participant 1: 47 years, 3 children, married, secondary education, screened**<br>What I have also heard is that cervical cancer is caused by using things like herbs in the private parts. This affects the cervix. Also use of perfumed soap can cause the cervix to swell and cause cancer. Another cause is that when your sexual partners has sex with another woman who has cervical cancer, he can carry it back to you. Having sex with many people be it the woman or the man increase the chances of having cervical cancer for the woman because you are likely to pick the disease from one of the partners.<br>**Interviewer:** What else would you like to tell me about cancer of the cervix?	Inserting herbs in vagina<br>Use of perfumed soaps in vagina<br>Sexual partner having sex with another woman who has cervical cancer<br>Multiple sexual partners
**Participant 3: 36 years, 1 child, married, secondary education, screened**<br>Another cause of cervical cancer is a history of criminal abortions. Others use sharp objects to induce abortions. It is possible to pierce the cervix and this causes a wound which later may turn into cancer.<br>**Interviewer:** Which class of women is more at-risk for cervical cancer?	Criminal abortions
**Participant 2: 29 years, 3 children, married, secondary education, screened**<br>People who are HIV positive are more at risk because their immune system is already not strong. So, an HIV positive person should be screened regularly.	HIV<br>Need for regular screening
**Participant 4: 42 years, 3 children, married, secondary education, screened**<br>Yes, the immune system is disturbed in HIV positive people and the soldiers have no power to fight so it's easy for a person to be attacked by any disease.	HIV
**Participant 2: 29 years, 3 children, married, secondary education, screened**	

Those who are more at risk are those aged 16-50 years, or I can say any age as long as you are having sexual relations. Those not having sex cannot get cervical cancer.<br>**Interviewer:** Could there be any other risk factors for cervical cancer apart from those which have already been mentioned?<br>**Participant 7: 27 years, 1 child, single, secondary education, screened**<br>Women who drink a lot are also more likely to get cervical cancer because alcohol also weakens the immune system.<br>**Participant 6: 34 years, 1 child, married, secondary education, not screened**<br>Smoking also increases the risk. They say it also lowers the body defence system.<br>**Interviewer:** Here in your community, are there any particular practices which increase women's chances of getting cervical cancer?<br>**Participant 5: 50 years, 4 children, married, secondary education, not screened**<br>Sleeping with other people's husbands. Women here have a bad habit of going out with a man when they know that he is married. They lure other people's husbands to sleep with them. That is very bad.<br>**Other participants**: Murmur, Yes<br>**Participant 4: 42 years, 3 children, married, secondary education, screened**<br>I wish to add on that. It happens that me having my husband, I will not be knowing that my husband will leave home and go to have sex with my neighbour. From my neighbour he will move to another woman. At the end of the day, he will have slept with many of us exchanging us. The sad part is that those other women agree to sleep with him when they know that he is married. This hide and seek game promotes spread of the disease because he will carry the it from A to B to C to D.<br>**Interviewer:** Anything else?<br>**Participant 1: 47 years, 3 children, married, secondary education, screened**<br>People here always suffer from sexually transmitted diseases. This increases the risk of developing cancer in our community as well.<br>**Interviewer**: There are warning signs which can make a woman suspect that they may have cervical cancer. Could you please name those which have not previously been mentioned?<br>**Participant 3**: **36 years, 1 child, married, secondary education, screened**<br>Bleeding most of the time even when you are not due for your monthly period could be a sign of cervical cancer.<br>**Participant 5: 50 years, 4 children, married, secondary education, not screened**<br>Isn't it that every woman has a vaginal discharge which is normal, but if you notice that the discharge is now of a different colour which is not the usual, this means that something is wrong in the cervix.	**Risk factors:**<br>16 – 50 years<br>Anyone in a sexual relationship<br><br>Drinking alcohol<br><br>Smoking<br><br>Multiple sexual partners (Sleeping with other people's husbands)<br><br>Partner with multiple sexual partners<br><br>Sexually transmitted diseases<br><br>Bleeding between periods<br><br>Unusual vaginal discharge

**Participant 7: 27 years, 1 child, single, secondary education, screened**<br>People with cervical cancer will always complain of pain the lower part of the abdomen and sometimes a smelly discharge.<br>**Interviewer:** Anything else?..... I see you have exhausted all that you had to say. How do you get the knowledge on the signs of cervical cancer and other information on cervical cancer in general?<br>**Participant 1: 47 years, 3 children, married, secondary education, screened**<br>We get the information from the nurses and Community Health Workers. We also get the information from church. I am a Seventh-day Adventist. In our church we have a health department which gives us these lessons.<br>**Participant 5: 50 years, 4 children, married, secondary education, not screened**<br>When we go to the clinic, nurses educate us on cervical cancer.<br>**Participant 3: 36 years, 1 child, married, secondary education, screened**<br>At the clinic we get lessons on cervical cancer every Wednesday. There are 2 nurses who always give us lessons on that.<br>**Participant 1: 47 years, 3 children, married, secondary education, screened**<br>In our church there are different departments including that of health. There is a person responsible for educating us on health matters. This person is a nurse. So, when that week the health department has been scheduled to give a lesson, they also teach us about cervical cancer.<br>**Interviewer:** How does the information reach those women who do not attend churches where lessons are given on cervical cancer, and those who do not frequently go to the clinic?<br>**Participant 5: 50 years, 4 children, married, secondary education, not screened**<br>In the church previously mentioned, they have also created a WhatsApp group whose administrators also include medical doctors. They always send messages to create awareness about cervical cancer and screening and ask people to forward the messages to their friends and neighbours. That way the information spreads fast because these days almost everyone has a cell phone and most people will not ignore messages. They read them and sometimes those we send messages to will come back with questions which gives us an opportunity to discuss further amongst ourselves and refer back to the WhatsApp group if there are things we do not understand. It works. This WhatsApp system is a good way which can be used to reach many people.<br>**Participant 1: 47 years, 3 children, married, secondary education, screened**	Low abdominal pain<br>Foul smelling vaginal discharge<br><br>**Sources of information:**<br><br>Clinic (Nurses)<br>Community Health Workers<br>Church<br><br>Clinic<br><br>Clinic<br><br>Church<br><br>Church based WhatsApp group messages<br>Innovative education strategies

The Community Health Workers also move door to door teaching us about cervical cancer. The frequency of these visits has however been reduced since COVID-19 started.<br>**Interviewer:** Having received the education and now being aware of the signs and symptoms of cervical cancer, what action would you take if you notice a sign or symptom that is suggestive of cervical cancer?<br>**Participant 5: 50 years, 4 children, married, secondary education, not screened**<br>I would go to the clinic for examination and treatment.<br>**Participant 2: 29 years, 3 children, married, secondary education, screened**<br>The usual practice is to go to the clinic. If you haven't been screened, they will also refer you to Gwanda for screening chop-chop.<br>**Interviewer:** Could there be any other actions that women would take?<br>**Participant 1: 47 years, 3 children, married, secondary education, screened**<br>Yes, there are some who run to traditional healers because they have not yet fully understood how dangerous cervical cancer is. Others also seek help from these churches which prophesy and also claim that they can treat cancer, instead of going to the clinic. They don't realise that cervical cancer is a very dangerous disease because if you delay, the disease will reach a stage where it can no longer be treated, and you end up dying.<br>**Participant 5: 50 years, 4 children, married, secondary education, not screened**<br>I see it in a different way. Sometimes women are afraid such that even if they see those signs, they won't do anything about it. Their reasoning is that even if they can be referred to Gwanda for treatment, it costs, and they have no money. They say, otherwise I will die of stress. So, some women find it better to remain with their symptoms and do nothing about it. So, the big challenge we have is that for treatment you have to pay. All that is free is the screening.<br>**Participant 2: 29 years, 3 children, married, secondary education, screened**<br>I would still encourage women to go for screening because even if you don't have money for treatment, you can make a payment plan and pay bit by bit so please don't stay at home when you notice any unusual things, let's go and get treated.<br>**Participant 3: 36 years, 1 child, married, secondary education, screened**<br>Yes, that's what the people from Gwanda told us when they came. They said even if women have no money to pay, they should just come to Gwanda and make arrangements for later payments.<br>**Interviewer:** As far as you know, is there a vaccine available to protect women from cervical cancer?	Door to door education by Community Health Workers<br>**Action when you notice S& S of cervical cancer:**<br>Go to the clinic<br>Go to the clinic<br>Visit traditional healer<br>Seek help from prophets<br>Cervical cancer is a dangerous disease<br>Do nothing<br>Anticipate referral to Gwanda<br>Lack of money transport<br>Fatalistic view of cervical cancer<br>Lack of money for treatment<br>Make payment plan<br>Make payment plan

**Participant 6: 34 years, 1 child, married, secondary education, not screened**<br>We have been told that people should protect themselves by using condoms one, and two, there is an injection which is given.<br>**Interviewer:** Can we discuss more on the injection?<br>**Participant 4: 42 years, 3 children, married, secondary education, screened**<br>Honestly speaking, I have never heard of such a vaccine which can protect someone from developing cervical cancer. The only prevention I know is to use condoms as someone has already said.<br>**Participant 3: 36 years, 1 child, married, secondary education, screened**<br>No, there is a programme where school girls aged 14 years were given injections and were told that it's for the prevention of cancer. But there I am not sure whether that's the vaccine you are talking about, but they said they only give it to young girls who have not yet started to have sex. So, we don't know because then if it's the one how does it protect you from having cervical cancer?<br>**Interviewer:** Anyone who would like to respond to that? ….. I see you would like to have more information on that. Yes, that's the vaccine I am talking about. It is called the Human papilloma virus (HPV) vaccine and is given to girls aged 10-14 years; the age group that is expected not to have started sexual activity. That is to ensure that they are protected at the time they start sexual relations should they be exposed to the HPV. It does not give protection to someone who is already sexually active. Please do get more information on this from the clinic.<br>**Participant 3: 36 years, 1 child, married, secondary education, screened**<br>Oh, thank you. I had never understood how it works.<br>**Interviewer:** Let's talk about the means which are available to detect cervical cancer early while it can still be treated.<br>**Participant 2: 29 years, 3 children, married, secondary education, screened**<br>We know about VIAC which is a screening method used to detect cancer cells then they treat you before it moves to the next stages.<br>**Participant 3: 36 years, 1 child, married, secondary education, screened**<br>There is also the Pap smear which is done in other places. In South Africa that's what they use and that's where I was screened.<br>**Interviewer:** Anything else?......Ok I see you have nothing else to add. In this district, where can you access cervical cancer screening services?<br>**Participant 2: 29 years, 3 children, married, secondary education, screened**<br>You can be screened using VIAC at Gwanda Provincial Hospital.<br>**Participant 1: 47 years, 3 children, married, secondary education, screened**	Lack of full information on vaccine<br>No knowledge of vaccine<br>Protect with condoms<br>Lack of full information on vaccine<br>**Screening methods:**<br>VIAC screening<br>Pap Smear<br>Used in South Africa<br>**Screening places:**<br>Gwanda Provincial Hospital

Screening is also done at the Sisters Clinic in Jahunda in Gwanda, next to the Police camp and there they use the Pap Smear.<br>**Participant 7: 27 years, 1 child, single, secondary education, screened**<br>Gwanda Hospital also conducts mobile screening services but after a very long time. They sometimes come to our clinic and they use VIAC. They bring their equipment and screen women here at the clinic.<br>**Interviewer:** Could someone explain more about the Sisters Clinic?<br>**Participant 1: 47 years, 3 children, married, secondary education, screened**<br>It is a clinic run by Red Cross and caters mostly for the "ladies of the night". They provide screening services using Pap smear, but they offer all other health services. That's where I was screened long back before Gwanda Provincial Hospital started doing VIAC. They took a specimen and sent it to Bulawayo for testing.<br>**Interviewer:** Thank you for the explanation. At what age should a woman start screening?<br>**Participant 2: 29 years, 3 children, married, secondary education, screened**<br>At any age as long as you are between 18 and 50 years you can go for screening. I am not so sure though, but I think so. Then if you are HIV positive you have to be screened every year, and if you are HIV negative every 2 years.<br>**Interviewer:** Anyone else who wants to say something on this?<br>**Participants:** Silence<br>**Interviewer:** How common is it for women in this community to have cervical cancer screening?<br>**Participant 4: 42 years, 3 children, married, secondary education, screened**<br>I think it's about a quarter of women in our community who have been screened, or even less than that. The reason is that when the mobile team comes, they pick who they want to screen. They don't just screen everyone who wishes to be screened. If the service could be offered to everyone, I am sure most women here would have been screened by now. The problem is they pick who they want to screen then the rest have to go to Gwanda to be screened but they don't go because they have no money.<br>**Participant 2: 29 years, 3 children, married, secondary education, screened**<br>Yes, they say they screen only those women who are HIV positive. Those are the ones they look for. But if that group does not come because some of them don't want their HIV status to be known, that's when they can screen other women.<br>**Participant 7: 27 years, 1 child, single, secondary education, screened**<br>They came here last year and the message that was delivered to the community was that they only wanted those who are HIV positive. So, most women did not go and when the outreach team found few	Sisters Clinic<br><br>Mobile screening services<br>Inconsistent visits by Mobile team<br><br>Sisters clinic offers health services to commercial sex workers<br><br>Incorrect information on screening ages<br>Incorrect information on screening frequency for HIV negative women<br><br>Few women screened<br>Mobile team has a target group<br>Recommend offering service to everyone<br>Lack of money for transport<br><br>Screen only HIV positive<br>Fear stigma and discrimination<br><br>Screen only HIV positive<br>Only screen others if those on ART don't come

people, they then said that everyone regardless of HIV status was supposed to have come. But when they came this year again, they had already booked those on ART which they wanted to see. They already had a list of names of the women they had come to screen but when those on ART did not come, I understand they ended up screening everyone who was at the clinic and wanted to be screened.	
**Interviewer:** Could those who have been screened please share their screening experiences?	**Screening experience:**
**Participant 1: 47 years, 3 children, married, secondary education, screened**<br>I was afraid initially to have the procedure done on me because of the stories I had been told that it's very painful. But it was not painful. I cannot remember if I was told when to come for repeat screening because at the time they gave the education, I was very nervous and afraid. Maybe it would be better if individualised education is also given after the procedure for it to be more effective. As it is, I don't even know when I am supposed to go back for rescreening.	Treated professionally<br>Lack of effective education on timing for repeat screen<br>Recommend individualised education after procedure
**Participant 2: 29 years, 3 children, married, secondary education, screened**<br>I was also very tense and scared of that big instrument that they insert into the vagina. But I was treated very well, and everything was explained to me. The procedure was a bit uncomfortable, having to lift up your legs; but it was not painful. I would like to encourage those women who have not been screened to go because honestly, it's for your own good ladies. I was relieved when I was told all was well but even if it was not, I would have been happy that now I can get help. The truth is even if you have not been screened, you are always worried, so get screened and then deal with the results.	Treated professionally<br>Procedure uncomfortable
**Participant 7: 27 years, 1 child, single, secondary education, screened**<br>I was screened at the VIAC clinic in Gwanda because I had not been well for a long time. I had gone to prophets many times and treated but never got any healing. I was treated well and got referred to UBH (central hospital). I'm glad I was screened at the time I did and got treated. If I had delayed, maybe I could be telling a different story today, that is, if I would be still around to tell it.	Was screened due to ill health<br>Treated professionally
**Interviewer:** What reasons do you think women have for not screening?	**Barriers to screening:**
**Participant 1: 47 years, 3 children, married, secondary education, screened**<br>Women are afraid of screening for different reasons. Others are afraid they will be told they have cancer then what? Others are afraid their wombs will be removed then they will be incomplete, and their marriages will be affected. Others are afraid of an imminent death once they know they have cancer, so they think it's better if they stay without knowing.	Fear of a cancer diagnosis<br>Myths and misconceptions<br>Lack of partner support<br>Fatalistic view of cervical cancer
**Participant 5: 50 years, 4 children, married, secondary education, not screened**	

Women have fear of the unknown because people out there say a lot of things about screening and its after effects. So, this lack of information is what makes women believe all the things they hear. Secondly, I think it is important for women to be continuously encouraged to screen through repeated reminders. As human beings, naturally we respond after repeated messages. Mass education campaigns should be done from time to time so that women realise that this thing is important. The mobile team should also screen all women and not only those on ART. That way, other women not in the targeted group will also get an opportunity to be screened without having to find money to go to Gwanda. Reminders on screening should be done at the clinic and by the Community Health Workers all the time not on certain days. Naturally, people take action upon constant reminders.	Myths and misconceptions<br>Lack of adequate knowledge on cervical cancer and screening<br>Lack of effective education programmes on cervical cancer<br>Periodic mass education campaigns<br>Mobile team to screen all women<br>Constant reminders at clinic and by CHWs
**Participant 7: 27 years, 1 child, single, secondary education, screened**	
I would like to emphasize on the earlier recommendation. The Mobile team should screen everyone not just those on ART. If they can't do it in one day, it's better they have a second day when they come for those not on ART so that everyone is covered. Otherwise what is the point of being told about the importance of screening then denied the opportunity to be screened? And better still, they should not say only those on ART because even if on ART, some women will not come for fear of being stigmatised by the community. I am on ART myself and I went to be screened but most of my counterparts did not come for fear of discrimination. We don't view these things in the same way.	Mobile team to screen all interested women<br>Schedule screening days appropriately<br>Education on screening, but opportunity to screen denied<br>Fear of stigma and discrimination
**Interviewer:** Anything else, may I encourage everyone to please participate?	
**Participant 4: 42 years, 3 children, married, secondary education, screened**	
Some women are just reluctant. As long as life goes on, they don't mind about other things. We kindly request that more education be given on a wide scale to reach everyone including those who do not show interest. Eventually they will understand.	Intensify education<br>Women aware of programme but lack interest (Apathy)
**Participant 2: 29 years, 3 children, married, secondary education, screened**	
Other women are afraid to test because they don't know how the husband will react to a positive result. They fear that the husband will leave her. They may take a second wife if the first gets cervical cancer. So, I suggest that men should also be involved in these lessons so that they also understand and encourage their wives. Find ways of involving men in these lessons when you teach women about this disease and the importance of screening.	Lack of partner support<br>Male involvement in education about cervical cancer
**Interviewer:** Could there be cultural beliefs which influence women's decisions to be screened or not to go for screening?	
**Participant 5: 50 years, 4 children, married, secondary education, not screened**	

It's more of the negative things which people tell you about screening, for example I have heard that after you are screened, you get a whitish discharge which never stops, and men don't like a watery woman. So, it's better if I don't screen and keep my husband.<br>**Interviewer:** Anything else?<br>**Participant 6: 34 years, 1 child, married, secondary education, not screened**<br>What might also prevent women from screening is their religious beliefs. Some churches discourage attending health services and they say they are able to heal illnesses. Women from these churches are not allowed to go to the clinic. We also have people who are still very traditional. They encourage the use of herbs to treat the vagina to remain healthy and this may later cause cancer.<br>**Interviewer:** We have discussed a lot of barriers to cervical cancer screening in this community and given solutions to some of the problems. Is there anything else you would like to see being done to improve access to screening services by all women?<br>**Participant 4: 42 years, 3 children, married, secondary education, screened**<br>I recommend that screening services be offered at local health facilities too so that we won't have the problem of finding money and time to go to Gwanda. If it is offered here then women can access the services anytime they decide to be screened.<br>**All Participants:** Yes, Yes.<br>**Participant 1: 47 years, 3 children, married, secondary education, screened**<br>Before the service can be provided at the local facilities, I suggest that the mobile team should come here more frequently like every month and cater for all women. And if they can give it two days. The last time they came here, when those on ART were not that many, the clinic started sending word that other people should come but when some people got there, they said their time was up and they had to leave. So, more time like dedicating 2 days to each facility would enable more women to be screened.<br>**Interviewer:** Any other recommendations to improve delivery of cervical cancer screening which we haven't discussed?<br>**Participant 5: 50 years, 4 children, married, secondary education, not screened**<br>Although this is a study it has also been educative. Thank you for coming.<br>**Interviewer:** I thank you all for your participation and hope that the information you have given me will contribute towards improving the programme so that more women will be screened.	Myths and conceptions<br>Lack of partner support<br><br>Religious beliefs<br>Socio-cultural beliefs<br><br>**Recommendations to increase screening rates:**<br><br>Provide screening services at local health facilities<br><br>Provide screening at clinic x6<br><br>Frequent mobile screening services<br>Schedule 2 days to cater for everyone
**FGD 4**<br>**Interviewer:** I would like to thank you ladies for agreeing to join this discussion. It is very important to understand why women are still not utilising the cervical cancer screening services in the way we expect. This could help find ways to address the problem. Now that we are all	

on the same page about the commonly and acceptably used terms for intimacy and reproductive health organs, we will begin our discussion. So, according to your knowledge, which are the most 3 common cancers which affect women in Zimbabwe? You can give them according to the most problematic.	
**Participant 3: 26 years, 2 children, married, primary education, not screened**<br>The most common reason for cervical cancer is starting to have sex at an early age and smoking.	Early sexual debut<br>Smoking
**Participant 7: 43 years, 3 children, married, primary education, not screened**<br>Interrupts: You did not understand the question. The first is cancer of cervix, then cancer of the breast. Those are the two I know.	Cervical cancer<br>Breast cancer
**Participant 1: 39 years, 2 children, married, primary education, screened**<br>Its cervical cancer, then breast cancer, then cancer of the throat. Those are the ones I have heard of people who suffered from them.	Cervical cancer<br>Breast cancer<br>Cancer of the throat
**Participant 6: 37 years, 3 children, married, secondary education, not screened**<br>There are many cancers as far as I know. There is cancer of the cervix, cancer of the breast, lung cancer. These are all dangerous because they kill.	Cervical cancer<br>Breast cancer<br>Lung cancer.<br>All cancers kill
**Participant 7: 43 years, 3 children, married, primary education, not screened**<br>The most common we hear about is that of the cervix, then breast cancer and last lung cancer.	Cervical cancer<br>Breast cancer<br>Lung cancer
**Interviewer:** As you have correctly mentioned cancer of cervix is the most common in Zimbabwe and is increasing and affecting more women. What do you know about cervical cancer? Just tell me anything you have heard about this disease.	
**Participant 4: 47 years, 3 children, married, primary education, screened**<br>Cervical cancer is a problem in Zimbabwe because women like using flannel materials as pads. Those particles from the cloth stick to the cervix and accumulate and can cause cancer later on. Two, women like using herbs to tighten the vagina, these herbs can eat the lining of the vagina and cause cancers. Three, some women like prostitutes will have sex with different men without bathing in between. This can cause cancer of the cervix.	Cervical cancer is a problem in Zimbabwe<br>Improvising sanitary pads with other materials<br>Inserting herbs in vagina<br>Multiple sexual partners<br>Poor sexual hygiene
Interviewer: What else can you tell me about cervical cancer?	
**Participant 5: 38 years, 1 child, married, secondary education, screened**<br>If you have cervical cancer you can feels sores inside in the cervix and have a smelly discharge coming out of the vagina. These sores will keep extending and turn into a wound. If you have this, then you have to go and see the doctors.	Feel sores in cervix<br>Foul smelling vaginal discharge

**Participant 4: 47 years, 3 children, married, primary education, screened**<br>One can get cervical cancer through sexual intercourse. If my husband gets it from another woman he can pass it on to me. If you have cancer of the cervix you cannot bear children, and then if you have a vaginal discharge which has a bad smell, it can be a sign that you may be having cervical cancer.	Sexually transmitted<br>Cannot bear children<br>Foul smelling vaginal discharge
**Participant 6: 37 years, 3 children, married, secondary education, not screened**<br>Just to add something, you can have cervical cancer and stay for a long time without any signs or symptoms and think you are alright when you are not. So, the most important thing is to go for screening. That is how you can know for sure whether you have it or not.	Long time with no signs and symptoms
**Participant 2: 30 years, 1 child, married, primary education, not screened**<br>Yes, it can stay 10-20 years without feeling pain. If one has many sexual partners, or my sexual partner has many sexual partners, it is very easy to get cervical cancer. Girls who start having sex when they are too young can also get cancer because their organs are not yet fully developed so it's easy to get it.	Long time with no signs and symptoms<br>Multiple sexual partners<br>Partner has many sexual partners<br>Early sexual debut
**Participant 4: 47 years, 3 children, married, primary education, screened**<br>If you feel that pain that feels like something eating you up down there, then you must know you have cancer of the cervix. It eats you up to the extent that you get very thin and you can even see your bones.	Pain that feels something is eating you up<br>Severe loss of weight
**Participant 5: 38 years, 1 child, married, secondary education, screened**<br>If you are HIV positive, chances of getting cervical cancer are very high because your immune system is weakened, and it is very easy to be attacked by any illness.	HIV
**Participant 7: 43 years, 3 children, married, primary education, not screened**<br>Cancer of the cervix is seen more in people who have children.	Women with children
**Participant 4: 47 years, 3 children, married, primary education, screened**<br>It's usually seen in people from 18 years going up.	Incorrect information on at risk age group
**Participant 1: 39 years, 2 children, married, primary education, screened**<br>From 10 years to 50 years.	Incorrect information on at risk age group
**Interviewer:** Please give reasons for your answers. Why are you saying those age groups are more at risk?	
**Participant 7: 43 years, 3 children, married, primary education, not screened**<br>That is because those people are in sexual relationships and cervical cancer can be transmitted sexually.	Cervical cancer is sexually transmitted
**Participant 1: 39 years, 2 children, married, primary education, screened**	

I say 10 years upwards because children start their menstruation very early at even 10 years and also start having sex early and if they do, they can have cervical cancer at that age.	Incorrect information on at risk age group
**Participant 5: 38 years, 1 child, married, secondary education, screened**<br>I think from 15 years because I see the injections given to school children to prevent cervical cancer start from 15 years.	Incorrect information on at risk age group
**Interviewer:** In this community, roughly at what age do you think girls start engaging in sex?	
**Participant 2: 30 years, 1 child, married, primary education, not screened**<br>13 years	Early sexual debut
**Participant 5: 38 years, 1 child, married, secondary education, screened**<br>15 years	Early sexual debut
**Participant 1: 39 years, 2 children, married, primary education, screened**<br>It depends on the child. These days children start menstruation early. Once that happens, they start engaging in sex even at 10 years.	Early sexual debut
**Interviewer:** Are there other things or behaviours which increase the risk of getting cervical cancer apart from that which has been mentioned?	
**Participant 1: 39 years, 2 children, married, primary education, screened**<br>If you suffer from sexually transmitted disease it's also easy to get cervical cancer.	Sexually Transmitted Infections
**Participant 3: 26 years, 2 children, married, primary education, not screened**<br>Smoking also causes cervical cancer	Smoking
**Participant 5: 38 years, 1 child, married, secondary education, screened**<br>Even inserting your fingers inside your vagina when bathing can cause cervical cancer because you don't know how clean your fingers are. They could be having some germs which cause cervical cancer.	Inserting fingers in vagina when bathing
**Interviewer:** What if I am certain that my hands are clean, is it safe to insert the fingers in the vagina?	
**Participant 4: 47 years, 3 children, married, primary education, screened**<br>I think you cannot avoid inserting your fingers there because you have to check every time that there are no sores there and you can only do that by using your fingers. So, what I can say is that women should always keep their nails short because those nails can keep dirt underneath and introduce it into the cervix, or scratch your private parts making it easy for the germs to get inside then you get cancer.	Inserting fingers if nails are long
**Participant 2: 30 years, 1 child, married, primary education, not screened**<br>As for me what we were taught that we should never insert fingers when washing our private parts. The cervix is able to clean itself. So,	Inserting fingers in vagina

there is no need to insert our fingers because we will hurt ourselves and it's easier for the cancer to stick on if it finds an open wound.<br>**Interviewer:** What are the other warning signs of cervical cancer to add onto those you have already mentioned?<br>**Participant 5: 38 years, 1 child, married, secondary education, screened**<br>You can have a lot of vaginal discharge although you won't be feeling any pain.	Profuse vaginal discharge
**Participant 1: 39 years, 2 children, married, primary education, screened**<br>The discharge will have a bad smell like rotten fish<br>**Participant 4: 47 years, 3 children, married, primary education, screened**<br>May I ask if cancer of the cervix is the same as fibroids? I once was operated on to remove fibroids.<br>**Interviewer:** No, a fibroid is a growth which is in the womb. Cervical cancer starts at the mouth of the womb.<br>**Participant 4: 47 years, 3 children, married, primary education, screened**<br>Thank you.	Foul smelling vaginal discharge
**Participant 2: 30 years, 1 child, married, primary education, not screened**<br>You can also feel pain when you pass urine.	Pain when passing urine
**Participant 5: 38 years, 1 child, married, secondary education, screened**<br>I think because the cervix is damaged, you can feel pain during sex, when he inserts the penis it is very painful because he will be touching on the wound. No, it won't be bearable.<br>**Interviewer:** How do you get to know this information about cervical cancer?	Pain during sex
**Participant 4: 47 years, 3 children, married, primary education, screened**<br>I personally get information from our church which is Masowe. All women are taught, and I am very satisfied with the education which the church gives. The Holy Spirit can visit you and tell you that you have cervical cancer, or so and so in the congregation has cancer which you need to attend to. The Holy Spirit will tell you how it should be treated. We give treatment but still advise the congregants to go for screening for proof because they will always be found negative as our treatment is effective.<br>**Interviewer:** Kindly explain on the treatment which is given.	**Sources of information:**<br><br>Apostolic Church<br><br>Content with quality of information received<br><br>Church gives treatment but also encourages screening
**Participant 4: 47 years, 3 children, married, primary education, screened**<br>You have to get the treatment before you go to the clinic. You cut your lemons and boil them, then you add cooking oil, and a bit of salt. Then boil these for a long time till the mixture becomes brown. Then we strain it using a cloth to make sure no particles remain. Then we soak cotton wool in the solution and insert it in the vagina and leave	Treatment consists of a concoction which is prepared then inserted into the vagina as a pack

it there for some hours. This pack pulls out all the impurities from the cervix and removes all cancer cells which will be starting. Even if its cancer that has progressed the dead cells get removed then fresh cells are pulled together. We have some women who had this cancer of the cervix in 2012 and we treated them. Up to now they are still well and healthy.<br>**Interviewer:** Ok. Where else do women get education on cervical cancer?<br>**Participant 1: 39 years, 2 children, married, primary education, screened**<br>We get lessons from the clinic and also from our Community Health Workers. We are also given information pamphlets so that you can read for yourself at home.<br>**Other Participants:** Start whispering, some start laughing, others shake their heads.<br>**Interviewer:** Can you share with us what you have? Please be free to say whatever you have. Can those who are whispering please share with us?<br>**Participant 7: 43 years, 3 children, married, primary education, not screened**<br>The truth is we have never seen any pamphlets. When the outreach team came, they gave a talk about cervical cancer, but we never got any pamphlets. Even at the clinic we have never been given any pamphlets<br>**Participant 1: 39 years, 2 children, married, primary education, screened**<br>A few of us got pamphlets when the outreach team came.<br>**Interviewer:** Is there any other way you get information on cervical cancer?<br>**Participant 5: 38 years, 1 child, married, secondary education, screened**<br>I once got a pamphlet from the clinic.<br>**Interviewer:** It is evident that some women have received pamphlets from the outreach team and some from the clinic and some have not.<br>**Participant 1: 39 years, 2 children, married, primary education, screened**<br>The mobile team from Gwanda is the one which brought pamphlets. They left a few and if you don't go to the clinic often you won't know they are there.<br>**Participant 6: 37 years, 3 children, married, secondary education, not screened**<br>For some of us who did not go to the clinic when the outreach team came, it is difficult to say whether or not the pamphlets were there, but I have never received pamphlets even when I visit the clinic.<br>**Interviewer:** Is there anything else?<br>**Participant 5: 38 years, 1 child, married, secondary education, screened**	Clinic<br>Community Health Workers<br>Information pamphlets<br><br>Mobile Team<br><br>Pamphlets given out by Mobile team<br><br>Pamphlets from clinic<br><br>Mobile team only distributed a few<br><br>Difficult to get information if you do not go to clinic regularly

The other reason which makes women reluctant to go for screening is because they are told wrong information by others. I was asked by one woman what exactly is done during screening. When I told her how I was screened she was surprised. What she had heard was that during screening the uterus is removed and put on a table or wherever then they inspect it. If it is not alright they treat it then put it back. The problem is that sometimes they fail to put it back in its proper place and then your cervix will always give you problems that's why women are scared of screening because they hear all sorts of stories which are not true.	Myths and misconceptions
**Participant 4: 47 years, 3 children, married, primary education, screened**<br>Although I have not used this method myself there some traditional healers who are able to treat this cancer. They boil some roots and give the person with the cancer to drink. The solution kills all the cancer cells even if they have spread to other parts of the body. I was once a member of the Zion Church; they also teach their members how to treat any type of cancer.	Traditional healers able to treat cervical cancer<br>Zion churches are able to treat cervical cancer
**Participant 6: 37 years, 3 children, married, secondary education, not screened**<br>I have never been screened myself, but I hear stories that the instrument that they insert down there causes much pain especially when they move it around looking for cancer. However, I still wish to be screened. I failed last time when the mobile team came because they said they only screen those on ART.	Myths and misconceptions<br>Outreach team targets HIV positive women<br>Women turned away<br>Still wish to be screened
**Interviewer:** May I check on how confident you are with the knowledge you have on cervical cancer to be able to identify its warning signs.	
**Participant 6: 37 years, 3 children, married, secondary education, not screened**<br>We do not have the full knowledge yet especially if you do not go to the clinic very often. Could we have other people who can gather us together in the community as women when we are free so that we are able to get full and correct information. We want to understand what this cancer of the cervix is, what causes it and how one can tell if they have it. Honestly speaking knowledge is lacking. We don't have the full knowledge.	Lack adequate knowledge about cervical cancer<br>Intensify education
**Participant 4: 47 years, 3 children, married, primary education, screened**<br>I don't know if I am wrong but, in our church Masowe, we are happy with the knowledge we receive on cervical cancer from the church.	Religious beliefs
**Interviewer:** Suppose you notice from what you have leant a symptom suggestive of cervical cancer, what would you do and how long would it take before you take that action?	**Action when you notice S&S of cervical cancer:**
**Participant 4: 47 years, 3 children, married, primary education, screened**<br>In our church people report immediately if they experience any illness and because if they don't, the Holy Spirit identifies you though the	

church leaders. So, people will always report to the church when they notice a problem.	Consult church leadership immediately
**Participant 7: 43 years, 3 children, married, primary education, not screened**<br>I would discuss the problem with my friend first then we advise each other on what I should do.	Consult friend for advice
**Participant 1: 39 years, 2 children, married, primary education, screened**<br>There is nothing else I would do except to go to the clinic.	Go to clinic
**Interviewer:** How common is it in this community for people to go to traditional healers to seek advice before they eventually go to the clinic?	
**Participant 6: 37 years, 3 children, married, secondary education, not screened**<br>Many people still go to traditional healers for problems to do with cancer because there, it can be completely healed. In hospitals, you are only given something to weaken the pain and they also burn the area where the cancer is, but they don't know how to completely cure it.	Consult traditional healers<br>Medically cervical cancer cannot be completely cured
**Participant 5: 38 years, 1 child, married, secondary education, screened**<br>I personally think that even if people go to these traditional healers, they still remain with doubts on whether they have been completely cured or no. So, I think it's better for those who prefer traditional healers to still go to the clinic so that they are examined to see if the disease has completely cleared.	Consult traditional healer but go to clinic too
**Interviewer:** According to the knowledge you have, is there a vaccine which can protect women from developing cervical cancer?	
**Participant 2: 30 years, 1 child, married, primary education, not screened**<br>Yes, it is there.	Vaccine available
**Interviewer:** Could you please elaborate on that?	
**Participant 2: 30 years, 1 child, married, primary education, not screened**<br>There is a vaccine which is only given to girls from 10-14 years. Not sure of the name. After that age, they don't give it because it is only given to those who have not started on sexual activities.	Inadequate knowledge on HPV vaccine
**Interviewer:** Anyone else?.....<br>**Participants:** No response	
**Interviewer:** What means are available to detect cervical cancer very early while it can still be treated?	**Methods to detect CC**
**Participant 5: 38 years, 1 child, married, secondary education, screened**<br>You can be screened.<br>**All other participants respond in unison**: Yes.	Screening
**Interviewer:** I see you are all in agreement that one can go for screening to detect cervical cancer early. Please elaborate on the	Lack of adequate information on screening methods

means of screening……..Ok, so, where are these screening services provided in the district?<br>**Participant 1: 39 years, 2 children, married, primary education, screened**<br>At Gwanda Provincial Hospital and I think they also screen at Manama Mission Hospital.<br>**Interviewer:** Apart from Gwanda and Manama would there be any other places which offer screening services?<br>**Participant 5: 38 years, 1 child, married, secondary education, screened**<br>We can also go to the clinic to be screened if the mobile clinic comes.<br>**Interviewer:** How frequent does the mobile team come?<br>**Participant 1: 39 years, 2 children, married, primary education, screened**<br>Ah, maybe once a year.<br>**Interviewer:** Any other places?<br>**Participants:** Shake their heads<br>**Interviewer:** From the knowledge you have, at what age should a woman start screening and how often should they be screened?<br>**Participant 1: 39 years, 2 children, married, primary education, screened**<br>Screening starts at 18 years or when you start having sex. If you are HIV negative you should be screened every 2 years, after every year if you are HIV positive.<br>**Interviewer:** How common is it for women in your community to have cervical cancer screening? May I please hear from all of you.<br>**Participant 3: 26 years, 2 children, married, primary education, not screened**<br>This programme only started coming to us through the outreach team last year and they don't come often so there are few who are screened. People don't have money to go to Gwanda.<br>**Participant 2: 30 years, 1 child, married, primary education, not screened**<br>And another thing, when the mobile team came, they said they only screen those who are HIV positive. Those who are HIV negative were told they could not be screened because their instruments are not enough.<br>**Participant 7: 43 years, 3 children, married, primary education, not screened**<br>So many of us were turned back. We also wish to be screened so that we know where we stand. But it seems the priority is given to women who are HIV positive.<br>**Interviewer:** For those who have been screened, could you please share your screening experiences?<br>**Participant 4: 47 years, 3 children, married, primary education, screened**<br>I was screened because I had a problem with fibroids. That was in 2017. Everything was done well, and I later had an operation to	**Screening places:**<br><br>Gwanda Provincial Hospital<br>? Manama Mission Hospital<br><br>Mobile clinic<br><br>Inconsistent visits by Mobile team<br><br>Correct information on screening age<br>Incorrect information on screening frequency for HIV negative women<br><br>Few women screened<br>Inconsistent visits by Mobile clinic<br>Lack of money to go to Gwanda<br><br>Mobile team targets HIV positive women<br>Women turned back, shortage of instruments<br><br>Many women turned back<br>Wish to be screened<br>Priority given to HIV positive women<br><br>**Screening experiences:**<br><br>Treated professionally

remove the fibroids. I have not had problems again, but I also have not been screened again, I get treatment from church.	Have not been screened again<br>Get treatment from Apostolic church
**Interviewer:** Ok. Does the church allow its members to go to hospital for screening?	
**Participant 4: 47 years, 3 children, married, primary education, screened**<br>Yes, even if we treat most conditions, the church does not refuse for its members to also seek medical treatment.	Church allows members to seek medical treatment
**Participant 1: 39 years, 2 children, married, primary education, screened**<br>I was screened at Selonga Clinic by the mobile team. The lady was friendly, and I did not experience any pain and we did not wait a long time before the service was provided.	Treated professionally<br>Short waiting period
**Participant 5: 38 years, 1 child, married, secondary education, screened**<br>I also did not experience any pain though I was very afraid of the procedure. The problem we have is that some women who screen will tell false and scary things about the procedure which discourages others from screening.	Treated professionally<br>Myths and Misconceptions
**Interviewer:** What could be the reasons which make women fail to access screening?	**Barriers to screening:**
**Participant 7: 43 years, 3 children, married, primary education, not screened**<br>The problem is that even if you get screened, if they find anything wrong, instead of getting treatment there you are told to go to Gwanda. Most fail to go because they have no money for transport and treatment. This makes women not to bother about screening. If it means dying, it's better to die because it's the same thing even if you are screened, you don't get treated.	Lack of money for transport and treatment<br>Renders screening pointless
**Participant 6: 37 years, 3 children, married, secondary education, not screened**<br>I will present my personal reason for not screening. When the outreach team came, I was having my period and I understand screening is not done on menstruating women. But I am ready to be screened anytime I get the opportunity.	Screening not done during menses
**Participant 2: 30 years, 1 child, married, primary education, not screened**<br>Some women do not go for screening because we hear that there is only one machine which is used on all women. So, the machine will screen a woman who has cancer and use the same machine on the next who does not have it. So, the one who did not have it may then get it from the machine.	Lack of confidence in health services
**Participant 4: 47 years, 3 children, married, primary education, screened**<br>Some women lack knowledge and they are also influenced by friends who also have no knowledge about the importance of screening. They then decide as a group that they will not be screened from the wrong information they have.	Lack of knowledge about screening<br>Peer influence<br>Myths and misconceptions

**Participant 5: 38 years, 1 child, married, secondary education, screened**<br>Some of us who have been screened, are the culprits of spreading wrong information which discourages others. The day I was screened, the lady who was screened before me came out frowning and exclaiming that it was very painful. I almost left without being screened.	Myths and misconceptions
**Participant 1: 39 years, 2 children, married, primary education, screened**<br>Laughing, others actually went back unscreened that day. They were now scared of the procedure.	Myths and misconceptions
**Participant 4: 47 years, 3 children, married, primary education, screened**<br>Some women are satisfied with the services they receive in church that's why they don't come. But we always encourage them to still go for screening	Religious beliefs
**Participant 2: 30 years, 1 child, married, primary education, not screened**<br>At times as women we mislead each other and go to traditional healers because of our beliefs. If we notice signs of cervical cancer, they may be able to treat but not always. So, it's better to go for screening and get assured help.	Peer pressure<br>Socio-cultural beliefs
**Interviewer:** How much support do men provide for screening programme?	
**Participant 4: 47 years, 3 children, married, primary education, screened**<br>Men understand if you explain it properly. They even provide us with money to go to hospital.	Men are supportive if well informed<br>Even provide transport money
**Participant 3: 26 years, 2 children, married, primary education, not screened**<br>Hey, it's difficult in other families. You see; if you go for screening, since you are already HIV positive, then you are told you also have cancer, it may create friction because your husband will say you brought the disease and it's your problem. He won't even give you the money to go to Gwanda.	Lack of partner support
**Participant 7: 43 years, 3 children, married, primary education, not screened**<br>I agree that men are a problem. They are very difficult to convince. They don't want even to be tested for HIV. If you talk about cervical cancer screening, he will ask you why you want to go looking for diseases and won't allow you to go for screening. They refuse and tell you that you can go if you want but if you are found to have the disease, it will be yours because you will have looked for it. Men can be very difficult.	Lack of partner support
**Interviewer:** After all the challenges we have discussed related to cervical cancer screening, what recommendations do you have for improving the service to make it more accessible to women?	**Recommendations to increase screening uptake:**

**Participant 2: 30 years, 1 child, married, primary education, not screened**<br>My recommendation is that educating the women about cervical cancer and screening should come first before women are invited for screening. If women understand these things well, they will willingly come for screening. The problem we have now **is that things are done in reverse. Awareness campaigns are centred on calling women for screening before educating them.** Start with the education and if women have understood the benefits of screening, they will be willing to be screened.	Education should come first before call for screening
**Participant 6: 37 years, 3 children, married, secondary education, not screened**<br>My recommendation is that whenever women are taught about cervical cancer screening, men should also be involved so that they understand that having cervical cancer is not the fault of the woman. That way they would be more supportive to their partners.<br>**Interviewer:** Anything else?	Male involvement in the education of women
**Participant 1: 39 years, 2 children, married, primary education, screened**<br>The problem is that the screening team from Gwanda only comes once a year. That means if during the time that they come one is not free to attend, then the screening passes you by. If they come the following year and maybe again you are not around or have prior commitments, you miss the opportunity again. My recommendation is that the mobile clinic should come more often, maybe 3 or 4 times a year so that every woman gets the opportunity to screen.	Mobile team should come more frequently
**Participant 7: 43 years, 3 children, married, primary education, not screened**<br>I also support the idea of male involvement because this can make everyone have an appreciation of the programme. This way, the number of women coming up for screening would increase. Education about the disease should also be done more so that women get to better understand why they need to be screened. If they understand the benefits of screening, they will want to be screened.	Male involvement<br>Intensify education of women
**Participant 4: 47 years, 3 children, married, primary education, screened**<br>I suggest that a committee for cervical cancer screening be formed in each village and trained on the subject so that they will have the responsibility of teaching the community on cervical cancer and screening. The community will agree on how often these learning sessions can be held but I think every Wednesday when people do not go to the fields. People will get full knowledge on cervical cancer and be motivated for screening and the number of women who develop the disease will be reduced.	Community involvement in education about screening
**Participant 5: 38 years, 1 child, married, secondary education, screened**<br>Even if community meetings are called, men will not attend. They will push women to the meetings like they normally do. **My suggestion is**	

**that there should be a programme which focuses on male cancers where men will also get the education more appropriate to them**. This will arouse their interest since the programme will be directly affecting them. As they get these lessons, they will stop blaming women for bringing such diseases home and women will get more support for screening.<br>**Participant 3: 47 years, 3 children, married, primary education, not screened**<br>We need more education. We have heard about this disease and that we should be screened but the knowledge we have is too little and there is still a lot which we would like to understand better.<br>**Other participants:** Yes, yes, yes.<br>**Interviewer:** I see. Could there be anything else to add?<br>**Participant 1: 39 years, 2 children, married, primary education, screened**<br>The other important thing which could get more women screened is that the Mobile clinic should not only focus on women who are on ART should offer the service to everyone of all age groups who is willing to be screened.<br>**Participant 2: 30 years, 1 child, married, primary education, not screened**<br>Education about cervical cancer should start in schools so that by the time these girls get to the age of screening, they will already be more informed about the programme.<br>**Participant 6: 37 years, 3 children, married, secondary education, not screened**<br>It would also help if information on cervical cancer is also delivered in our health clubs where we are taught about cleanliness in our homes and diseases like TB and Malaria. That's another platform the Community Health Workers can use.<br>**Participant 4: 47 years, 3 children, married, primary education, screened**<br>The Community Health Workers visit us in our homes to teach us. But when it comes to other programmes like COVID-19, special teams from the Ministry of Health come down to Village level. The same should be done from time to time because that way, people get to understand that the programme is very important for people to come all the way to teach about that subject. It can help to have the same thing happen with cervical cancer.<br>**Participant 5: 38 years, 1 child, married, secondary education, screened**<br>The mobile team should open screening to all women whether positive or negative and to all ages without choosing a particular group. This will help a lot of women to be screened. People have no money to go to Gwanda.<br>**Interviewer:** Anything else<br>**Participants:** Murmur, no.	Programme which focuses on male cancers<br>We are aware of the disease and screening<br>Inadequate knowledge about cervical cancer and screening<br>Intensify education<br>Mobile clinic to screen all women<br>Find innovative ways to educate people about cervical cancer<br>Find innovative ways to educate people about cervical cancer<br>Find innovative ways to educate people about cervical cancer<br>Mobile team should screen everyone<br>Lack of transport money to go to Gwanda

**Interviewer:** I see you have exhausted all you had to say. Thank you very much for your participation and for all your contributions. The information you gave will be used to come up with recommendations towards improving the cervical cancer screening programme in the district. Thank you for your time.	
**FGD 5**<br>**Interviewer:** I would like to welcome you to this focus group discussion and thank you for coming. We are still continuing with our investigation where we are looking at the barriers to cervical cancer screening in the district. Since we have agreed on the sexual and reproductive health terms which you use in this area, we will now start.<br>Which do you think are the 3 most common cancers among women and why is that?<br>**Participant 1: 26 years, 2 children, secondary education, screened**<br>The one which is always talked about is cervical cancer, followed by breast cancer.<br>**Participant 6: 44 years, 6 children, married, primary school, screened**<br>It's first cervical cancer, then breast cancer and then cancer of the womb.<br>**Interviewer:** Anyone else?.......No? Ok. The two answers given were that cervical cancer is the most prevalent in Zimbabwe. What could be the reason for this?<br>**Participant 10: 38 years, 2 children, married, primary education, screened**<br>It's because it's a sexually transmitted disease and women in Zimbabwe start being active at an early age so they are likely to get it from the many sexual partners they are likely to meet.<br>**Participant 5: 25 years, 2 children, married, primary school, not screened**<br>Women have no power to protect themselves during sex. It's men who have the power to say how they want it done and they don't want to use condoms in marriage so they can bring it from their other sexual partners.<br>**Participant 10: 38 years, 2 children, married, primary education, screened**<br>The other reason is that men have many sexual partners, so the disease is likely to be transferred to many women.<br>**Participant 7: 50 years, 6 children, single, secondary education, not screened**<br>It is also because a lot of women use herbs and other different things inserted in their private parts to dry up their vaginas for satisfaction of their partners during sex. This causes damage to the cervix and can result in cancer.<br>**Interviewer:** Anything else? …………Ok. What can you tell me about cervical cancer? It is a public health problem and the number of	Cervical cancer<br>Breast cancer<br><br>Cervical cancer<br>Breast cancer<br>Cancer of the womb<br><br>Early sexual debut<br>It is a sexually transmitted disease<br><br>Socio-cultural practices<br><br>Partner with multiple sexual partners<br><br>Inserting herbs in vagina

women with cervical cancer continues to rise in Zimbabwe. What do you know, or have heard about this disease?<br>**Participant 3: 31 years, 3 children, married, primary education, screened**<br>What I have heard about cervical cancer is that when you have it, you will have a vaginal discharge which will not the normal and it will be smelling.<br>**Participant 2: 40 years, 10 children, married, primary education, not screened**<br>I hear that if you have AIDS, the possibility of getting cancer of the cervix is very high because your body will be too weak to fight off diseases.<br>**Participant 11: 43 years, 6 children, widowed, primary education, not screened**<br>When women insert fingers down there when bathing, the nails can damage the cervix and result in cuts which can later change to cancer. So, we have been taught not to insert our fingers in the vagina when bathing. The nurses say it is enough to clean just the outside of the private parts without going inside.<br>**Participant 4: 34 years, 3 children, married, secondary education, screened**<br>Using perfumed soap to clean inside the vagina can also cause cancer the cervix to swell and then later cancer can develop.<br>**Participant 10: 38 years, 2 children, married, primary education, screened**<br>Using toilet paper for padding during menses results in the accumulation of paper particles in the cervix. After sometime this may cause cancer to develop.<br>**Interviewer:** Which other factors which have not been mentioned could increase a woman's chances of developing cervical cancer?<br>**Participant 10: 38 years, 2 children, married, primary education, screened**<br>Using some herbs and other things which may disturb the natural state of the vagina which makes it very easy for a woman to have cancer of the cervix. This is because the natural secretions of the vagina are disturbed. Those substances are absorbed by the cervix and poison it. We were also taught that we should not keep pubic hair because the germs which cause cervical cancer can hide there and this puts the woman in danger of getting the disease if the germs leave the pubic hair and get into the vagina and move up to the cervix.<br>**Participant 7: 50 years, 6 children, single, secondary education, not screened**<br>Sleeping with many people leads to cervical cancer. Like some men have up to 7. So, they can pass the cancer cells from one woman to the next.<br>**Participant 8: 50 years, 3 children, single, primary education, not screened**	Unusual vaginal discharge<br>Foul smelling vaginal discharge<br>HIV<br>Inserting fingers in vagina<br>Use of perfumed soap in vagina<br>Improvising sanitary pads with other materials<br>Inserting herbs in vagina<br>Long pubic hair<br>Partner with multiple sexual partners

People believe that once you have cancer, the next thing is death because the disease cannot be treated. So, they are scared to know that they have cancer. That is why some women will avoid screening because they say knowing that they have the disease will bring them stress.	Fatalistic view of cervical cancer<br>Fear of a cancer diagnosis
**Participant 5: 25 years, 2 children, married, primary school, not screened**<br>A person can also get cancer through witchcraft. If you go out with a married man, his wife can bewitch you so that you get this cancer of the cervix when her husband has sex with you. First, you can get pain in the cervix, then you start discharging pus from the vagina. Eventually the cervix will protrude through the vagina and with time it starts to rot. Those are the results of promiscuity.	Cervical cancer is due to witchcraft<br>Pain in the cervix<br>Unusual vaginal discharge<br>Cervix protrudes through vagina
**Participant 7: 50 years, 6 children, single, secondary education, not screened**<br>I would also like to add onto this point. If you snatch someone's husband, it's painful to that man's wife. So, the wife will work on her husband's penis and when that man has sex with you, the preparation will be transferred to you and you start to rot inside. The medical people refer to it as cancer of the cervix. It is very painful for another woman to be always shedding tears because of people who lust after other people's husbands. What I can advise women is to stay away from other people's husbands then you won't invite cervical cancer to yourself.	Cervical cancer is due to witchcraft
**Interviewer:** You have said what you know about cancer of the cervix. How would you rate the knowledge that women have on cervical cancer in general?	
**Participant 4: 34 years, 3 children, married, secondary education, screened**<br>The knowledge is there although it is still low. I say so because when we hear of a death from cervical cancer, people always say someone had a hand in that death. **So that means as a community, our understanding of cancer is still lacking.**	Inadequate knowledge on cervical cancer
**Participant 3: 31 years, 3 children, married, primary education, screened**<br>In my opinion, women have the knowledge, but they fear to be screened.	Women have knowledge<br>Fear to be screened
**Participant 10: 38 years, 2 children, married, primary education, screened**<br>I also think that women have the knowledge. The problem is that the hospital where screening is done is far such that even if you may have a desire to be screened, money to travel to Gwanda for screening is a challenge because now transporters charge in forex. Women have the knowledge but the other reason is that most are not comfortable with the way the screening is done. It is not an easy thing to just open up your private parts for someone when you are not ill. Somehow it's just wrong.	Women have the knowledge<br>Screening hospital far<br>Lack of transport money<br>Modesty issues

**Interviewer:** Someone mentioned that women are afraid of screening. May l hear more about that.<br>**Participant 1: 26 years, 2 children, secondary education, screened**<br>Women fear that instrument which is inserted into the private parts because they think it is painful. Then opening it up when it is inside eish.<br>**Participant 8: 50 years, 3 children, single, primary education, not screened**<br>Other women would have neglected working on their private parts as is expected of a full woman. So, they will be embarrassed and think the nurses who screen will laugh at them.<br>**Interviewer:** Could someone please elaborate on that one.<br>**Participant 7: 50 years, 6 children, single, secondary education, not screened**<br>I will expound on that. Culturally a full proper woman should have 2 ears in her private parts. They don't just appear on their own but during adolescence, girls are taught that they should pull those ears so that they hang out. This gives sexual pleasure to her partner and also covers the opening into the inside of her private parts so that she does not get prone to diseases of the sexual and reproductive health organs. That is what she means. So, if your private parts are naked, it is a shame to your womanhood which make women embarrassed to go for screening.<br>**Participant 3: 31 years, 3 children, married, primary education, screened**<br>For some women it's not about the missing ears, it's just the embarrassment to expose yourself.<br>**Participant 2: 40 years, 10 children, married, primary education, not screened**<br>Others are afraid that they may screen and are told they have cervical cancer. These days we are also not very confident with hospitals. We have heard that operations are done, and things go wrong. So that's why women are afraid to screen in case something goes wrong. Women now tend to combine both traditional methods with medical treatment just to be on the safe side.<br>**Interviewer:** What else can increase a woman's chances of developing cervical cancer?<br>**Participant 9: 44 years, 6 children, married, primary education, screened.**<br>Staying without screening increases chances of developing cervical cancer. If you don't screen, the cancer will not be seen and then it will continue to progress till you reach the stage where it cannot be treated.<br>**Participant 8: 50 years, 3 children, single, primary education, not screened**<br>The other danger is sleeping with John today, tomorrow with Joseph and then again with Mike. The discharges from all those 3 will be left in you and chances of meeting one who will transmit the disease to	Procedure is painful<br>Modesty issues<br>Modesty issues<br>Modesty issues<br>Fear of a cancer diagnosis<br>Lack of trust in hospital services<br>Belief in combining traditional and medical methods<br>Lack of screening increases risk of cervical cancer<br>Multiple sexual partners<br>Inserting herbs in vagina

you is higher than on someone who only sleeps with Tom. The other risk is inserting herbs into the vagina to tighten the muscles for sexual pleasure. Herbs eat away at the cervix and cause sores which may later change to cancer.	**Sources of information:**
**Interviewer:** How do we get information on cervical cancer and screening procedures?	
**Participant 6: 44 years, 6 children, married, primary school, screened**<br>When we go to the clinic, sometimes we find the nurses teaching about cervical cancer that day. So, I can say we get education from the clinic.	Clinic
**Participant 10: 38 years, 2 children, married, primary education, screened**<br>Once a team came from Gwanda to screen women. They gave us some education before they started screening. We also have Community Health Workers who visit us in our homes to tell us about this disease and also to advise us that it is important to be screened. That's where we also get information then they tell us to go to the clinic if we need further information.	Mobile team<br>Community Health Workers
**Interviewer:** Anything else to add?.... OK. Are there any other warning signs of cervical cancer you would want to add? You have already mentioned some of them.	
**Participant 1: 26 years, 2 children, secondary education, screened**<br>A vaginal discharge which is blood stained with pus.	Unusual vaginal discharge
**Participant 7: 50 years, 6 children, single, secondary education, not screened**<br>A woman may experience pain below the umbilicus because the cervix will be swollen.	Lower abdominal pain
**Participant 1: 26 years, 2 children, secondary education, screened**<br>I have also heard that at times you do not feel any pain at all even if you have that cancer. By the time you start experiencing any symptoms, usually the cancer will be in advanced stages.	No symptoms in early stages
**Participant 10: 38 years, 2 children, married, primary education, screened**<br>If you feel a burning sensation in your private parts, that pain which feels as if fire has been lit inside your private parts, it might be that you now have cancer of the cervix. The urine may also have a bad smell because the cervix will be rotting.	Burning sensation in private parts<br>Bad smell in urine
**Interviewer:** What then would you do if you notice any signs which you suspect may indicate that you have cervical cancer?	**Action when you notice s& s of cervical cancer:**
**Participant 5: 25 years, 2 children, married, primary school, not screened**<br>I would immediately go to the clinic.	Go to clinic immediately
**Participant 2: 40 years, 10 children, married, primary education, not screened**<br>I would start with the Community Health Worker because the clinic is far. I would tell her my problem and she would advise on what to do.	Consult Community Health Worker

Usually if it's something she cannot help you with, they tell you to go to the clinic.	
**Participant 3: 31 years, 3 children, married, primary education, screened**<br>Usually women will start at the clinic immediately they feel unwell and if the clinic treats you and you get well, fine. But if nothing changes or things get worse, then we may think of going to consult either traditional healers or prophets depending on your beliefs.	Go to clinic immediately<br>Consult traditional healers or prophets if nothing changes
**Participant 7: 50 years, 6 children, single, secondary education, not screened**<br>One may start by monitoring the situation for about 2 days maybe up to a week. You don't want to run to the clinic in case the problem resolves itself without the need for medication. If after a week the signs or symptoms have not cleared, then I go to the clinic.	Do nothing for a few days before you run to the clinic
**Participant 4: 34 years, 3 children, married, secondary education, screened**<br>Yes, not rushing to the clinic is because in the first one or two days, you may think you're not feeling well as a result of overworking because we work hard. You decide to rest and see if it will become better. If it does not change, then the next thing is to go to the clinic.<br>**Interviewer:** Let's move on. As far as you know, is there a vaccine which can be given that protects against cervical cancer?	Do nothing for a few days, then go to clinic if no change
**Participant 1: 26 years, 2 children, secondary education, screened**<br>Yes, these days there is a vaccine that is given to girls at school to prevent them getting cervical cancer when they become adults.	Inadequate information on HPV vaccine
**Participant 2: 40 years, 10 children, married, primary education, not screened**<br>I don't know of any such vaccine. The best thing is to seek advice from the clinic on these things.	No knowledge of HPV vaccine
**Participant 5: 25 years, 2 children, married, primary school, not screened**<br>There is a vaccine which is given to girls. It starts at 10 years. Girls who are 10 years old.<br>**Interviewer:** Is there anyone who would like to add anything about the vaccine?	Inadequate information on HPV vaccine
**Participant 1: 26 years, 2 children, secondary education, screened**<br>It's given to girls from 10 to 14 years in schools. The reason it is given to girls is because these days children engage in sex very early.	Inadequate information on HPV vaccine
**Participant 7: 50 years, 6 children, single, secondary education, not screened**<br>I think the girls are examined first to see if they have started having sex, then those who have started are given the vaccine.<br>**Interviewer:** May I just clarify on that. The girls are not examined, they are just vaccinated against the Human papilloma virus which is the primary cause of cervical cancer. The vaccine is called HPV and is only effective when one has not yet started on sexual activities to reduce their chances of acquiring the Human papilloma virus at the time when they start sexual relations and are exposed to that virus	Inadequate information on HPV vaccine

which causes cervical cancer. It does not work on a woman who is already sexually active.	
**Participant 7: 50 years, 6 children, single, secondary education, not screened**<br>Oh!	
**Interviewer:** Let's talk about means which are used to detect cervical cancer in its early stages when it can still be treated. Remember earlier some of you highlighted that if identified late, the precursor lesions progress to cancer and it may be too late to cure it.	
**Participant 4: 34 years, 3 children, married, secondary education, screened**<br>There is nothing else except for someone to go for screening.	Screening
**Participant 10: 38 years, 2 children, married, primary education, screened.**<br>The vaccination which was mentioned is very important. Girls should be protected when they are still young so that they do not get this cancer of the cervix.	Vaccination is important
**Interviewer:** Anyone who would like to elaborate on the screening methods and the purpose of screening?	
**Participant 2: 40 years, 10 children, married, primary education, not screened**<br>Screening helps for them to see the cancer so that you can be treated	Purpose of screening not fully understood
**Interviewer:** Any other purpose it serves apart from detecting early cancer?	
**Participants:** No response.	**Screening places:**
**Interviewer:** Ok. Which places do you know in the district where screening services are available?	
**Participant 10: 38 years, 2 children, married, primary education, screened**<br>At Gwanda Provincial Hospital.	Gwanda Provincial Hospital
**Interviewer:** Are there any other places where screening is done?	
**Participant 2: 40 years, 10 children, married, primary education, not screened**<br>Screening is only done in Gwanda that's why some of us have not been screened although we want because it is expensive to go to Gwanda.	Gwanda Provincial Hospital<br>Lack of transport money<br>Wish to be screened
**Interviewer:** Let us discuss which category of women need to be screened and why, and how frequently they need to be screened.	
**Participant 5: 25 years, 2 children, married, primary school, not screened**<br>All women who are 25 years and upwards need to be screened.	Incorrect information on screening age
**Participant 6: 44 years, 6 children, married, primary school, screened**<br>I think screening starts at 15 years so that this disease does not spread fast. Girls now start having sex very early.	Incorrect information on screening age
**Participant 10: 38 years, 2 children, married, primary education, screened**	

After every 3 years a woman should be screened. If you are screened today then you have to be screened again after 3 years going forward.<br>**Interviewer:** Anything else? ………..Ok, I see there is nothing. According to your assessment, how common is it for women in this community to be screened?	Correct information on screening frequency<br>Generally, no knowledge of screening frequency
**Participant 1: 26 years, 2 children, secondary education, screened**<br>Of the few women I associate with, many have been screened.	Few women screened
**Participant 3: 31 years, 3 children, married, primary education, screened**<br>As for me, I think there are only a few women who have been screened. Most women cannot afford transport costs to go to Gwanda for screening.	Few women screened<br>Lack of transport money
**Participant 4: 34 years, 3 children, married, secondary education, screened**<br>But there was a team which once came to screen people at the clinic. Many women were screened.<br>**Interviewer:** Earlier on when I asked where one can access screening services, the response I got was that screening services are only offered at Gwanda Provincial Hospital. So where was this team which came to screen women coming from? I need to understand about the facilities and organisations which offer screening services in the district.	(Many screened by Mobile team which came **once)**<br>Therefore, generally few women screened
**Participant 1: 26 years, 2 children, secondary education, screened**<br>The clinic sent a message through the Community Health Workers that there was a team coming to screen people at the clinic. Many people went there and were screened.	Mobile team
**Participant 9: 44 years, 6 children, married, primary education, screened.**<br>We don't know where they were coming from and we did not ask since we were told by the clinic to come.	Clinic advised of Mobile team visit
**Participant 11: 43 years, 6 children, widowed, primary education, not screened**<br>Going back to the question you asked, I think very few women have been screened. If it is possible, those people should come back again and screen us because a lot of education has been given and now, we understand better than last year (2019) when they came.	Few women screened<br>Mobile team should come frequently<br>We want to be screened
**Interviewer:** What could be the reasons why women keep postponing screening and some even having no desire to be ever screened and some having the desire but failing to access the service? You have touched on some reasons but perhaps you could add on to those.	**Barriers to screening:**
**Participant 1: 26 years, 2 children, secondary education, screened**<br>Women want to be screened but the main challenge is that they have no money to go to Gwanda to have that screening done. Women try to look for money over a long period so that they can go to Gwanda but before they get enough, the bus fare goes up. It's really a problem. The ones who are better are those with forex because the fare does not change for them.	Women want to be screened<br>Lack of transport money

**Participant 2: 40 years, 10 children, married, primary education, not screened**<br>The best thing that can be done for women is that those who screen should come here more often so that everyone can be free to have screening without the challenge of thinking about money. Yes, they should come and screen us here. That will help a lot of women to get screened.	Mobile team is inconsistent<br>Mobile team to come more frequently
**Participant 10: 38 years, 2 children, married, primary education, screened**<br>The mobile team has only come once. I also suggest that there should be a mobile clinic for screening coming here every month or at least every 3 months to screen people. I have also heard that if they find anything wrong when they screen you, they will ask you to pay for the treatment and sometimes they will say you need to be operated. That calls for large sums of money. Most of the time women do not have that money. So, in a way you have brought stress to yourself since you have started something you cannot see through.<br>**Interviewer:** Apart from the challenge of money to go to Gwanda, are there any other barriers which women face?	Mobile team inconsistent<br>Mobile team to come more frequently<br>Lack of money for treatment if result is positive
**Participant 7: 50 years, 6 children, single, secondary education, not screened**<br>The problem is money. Women want to be screened but have no money to go to Gwanda where screening is done. Although we may talk about other things, money is our major challenge.	Women want to be screened<br>Lack of transport money
**Participant 2: 40 years, 10 children, married, primary education, not screened**<br>Could you please send our request that we do not have money to go to Gwanda so it would be better for our clinic to also provide screening services so that screening is done here at the clinic instead of us having to travel to Gwanda because we do not have the money to go there.	Lack of money for transport<br>Screening services should be provided at the local clinic
**Participant 11: 43 years, 6 children, widowed, primary education, not screened**<br>Yes, money is a challenge, but the other thing is that our men do not want us to be screened. They think that if you go for screening, your womb will be removed. They do not understand why screening is done.<br>**Interviewer:** You have mentioned a lot of barriers which prevent women from screening and the challenge of money came up many times. So, what would you like to see being done differently in order to improve the screening programme so that more women are screened?	Lack of transport money<br>Lack of partner support<br>Myths and misconceptions
**Participant 11: 43 years, 6 children, widowed, primary education, not screened**<br>Men should also be involved in the education of women on cervical cancer screening. Maybe if they understand why it is done, they will encourage their partners to be screened. It is not very helpful to teach	Male involvement in education about cervical cancer

women alone. We should be taught together with our partners. Men believe that if your womb is removed, you will be hollow and unable to have sex so we should be taught together.<br>**Other participants:** Yes, yes, that's true.	Myths and misconceptions
**Participant 2: 40 years, 10 children, married, primary education, not screened**<br>I support the idea of involving men. If he understands what causes cervical cancer and what puts a woman in danger of getting the disease, he will understand better because our men are the ones with many sexual partners who bring home these diseases. If they understand why women should be screened, they will support us.	Male involvement in education about cervical cancer
**Participant 7: 50 years, 6 children, single, secondary education, not screened**<br>I recommend that our Community Health Workers should schedule a day every month where they call all women together and give us these lessons on cancer of the cervix. If you hear the same thing being talked about over and over, you are forced to take action. After all, these people stay with us and as women we won't need to go to the clinic, and we get our lessons closer. They tell us about most programmes which are taking place.<br>**Interviewer:** What can be done to address the challenge of money to go to Gwanda which appears to be the major challenge?	Intensify education
**Participant 3: 31 years, 3 children, married, primary education, screened**<br>Involving men when teaching us will solve the problem because they are the ones who give us money. So, if they are involved, they will freely give us the money to go to Gwanda for screening.	Male involvement
**Participant 7: 50 years, 6 children, single, secondary education, not screened**<br>Let us also remember that some women do not have those men in their lives so screening will still remain a challenge. A solution should be found which covers even those without men to give them money to go to Gwanda.<br>**Interviewer:** What do others recommend for an all embracive solution? Please let's hear from everyone.	Find innovative solutions which benefit everyone
**Participant 7: 50 years, 6 children, single, secondary education, not screened**<br>I think it is better if our community leaders meet with the Health Committee and send a request to Gwanda for screening to be provided right here at this clinic. Our Councillor should also take this request up.<br>**Participants:** Yes, yes that's the best solution.	Community involvement to request screening services by local clinic
**Participant 4: 34 years, 3 children, married, secondary education, screened**<br>I would also like to suggest that if men are to be called to join women when we are being taught about cervical cancer, this be done through the community leaders. Without doing that, men will never come. They are stubborn and don't like to attend any meetings unless if it	Involve community leaders in the education of men

has to do with them directly. Even for school meetings, they will send women.<br>**Interviewer:** Any other recommendations to improve access of the screening services?<br>**Participant 7: 50 years, 6 children, single, secondary education, not screened**<br>On behalf of all the women here, I would like to thank you for coming to meet us. We have shared our concerns about the screening programme for cervical cancer and hope you will take them up to the relevant authorities. We really want to be screened but it is not easy.<br>**Interviewer:** I would also like to thank you all for participating. Everyone who was asked to come did. I have heard all your views and at the end of this study I will submit a report to the Gwanda district health authorities on the recommendations which have been given, but please do take your requests to the health facility staff they will know how to channel them to the relevant offices. Thank you very much for coming.	Take request to relevant authorities

FGD 1 (Urban)	FGD 2 (Urban)	FGD3 (Mining community)	FGD4 (Rural)	FGD5 (Rural)
**General knowledge on CC:**<br>Most female common cancers<br>✓ Cervical cancer x3<br>✓ Breast cancerx1<br><br>Risk factors for cervical cancer<br>✓ Inserting herbs in vagina x3<br>✓ Witchcraft x3<br>✓ Multiple sexual partners x 3<br>✓ Inserting fingers in vagina x 2<br>✓ HIV x 1<br>✓ Using perfumed soap in vagina x 1<br>✓ Poor menstrual hygiene x1<br>✓ Anyone who is sexually active x1<br>✓ Older women x1<br>✓ Sexually transmitted Infections<br><br>Signs and symptoms	**General knowledge on CC:**<br>Most common female cancers<br>✓ Cervical cancer x2<br>✓ Breast cancer x2<br>✓ Cancer of the stomach x 1<br><br>Risk factors for cervical cancer<br>✓ Inserting herbs in vagina x 2<br>✓ Partner with multiple sexual partners x 1<br>✓ Sexually transmitted Infections x1<br>✓ Early sexual debut x1<br>✓ Socio-cultural practices x1<br>✓ Smoking x1<br>✓ Drinking alcohol x1<br>✓ Witchcraft x1<br>✓ Inserting fingers in vagina x1<br>✓ Use of perfumed soap in vagina x1<br><br>Signs and symptoms<br>✓ Unusual vaginal discharge x1	**General knowledge on CC:**<br>Most common female cancers<br>✓ Cervical cancer x 1<br>✓ Breast cancer x 1<br>✓ Cancer of the skin<br><br>Risk factors for cervical cancer<br>✓ Inserting herbs in vagina x 2<br>✓ Partner with multiple sexual partners x 2<br>✓ Multiple sexual partners x2<br>✓ Sexually transmitted Infections x 2<br>✓ Early sexual debut x1<br>✓ HIV x 2<br>✓ Smoking x 1<br>✓ Drinking alcohol x 1<br>✓ Anyone who is sexually active<br>✓ Criminal abortions x 1<br>✓ If partner has sex with someone who has cervical cancer<br>✓ Use of perfumed soap in vagina x1<br>Signs and symptoms<br>✓ Unusual vaginal discharge x 2	**General knowledge on CC:**<br>Most common female cancers<br>✓ Cervical cancer x 4<br>✓ Breast cancer x4<br>✓ Lung cancer x 2<br>✓ Cancer of the throat x1<br>Risk factors for cervical cancer<br>✓ Inserting herbs in vagina x 1<br>✓ Inserting fingers in vagina x 3<br>✓ Multiple sexual partners x 1<br>✓ Sexually Transmitted Infections x 2<br>✓ Early sexual debut x 4<br>✓ HIV X 1<br>✓ Smoking x 1<br>✓ Poor sexual hygiene x 1<br>✓ Having children x 1<br>✓ Improvising sanitary pads with other materials x 1<br><br>Signs and symptoms<br>✓ Unusual vaginal discharge x 1	**General knowledge on CC:**<br>Most common female cancers<br>✓ Cervical cancer x 2<br>✓ Breast cancer x 2<br>✓ Cancer of the womb x 1<br><br>Risk factors for cervical cancer<br>✓ Inserting herbs in vagina x 2<br>✓ Inserting fingers in vagina x 1<br>✓ Multiple sexual partners x 1<br>✓ Socio-cultural practices x1<br>✓ Early sexual debut x 1<br>✓ Partner with multiple sexual partners x 1<br>✓ Witchcraft x 2<br>✓ Using perfumed soap in vagina x 1<br>✓ Improvising sanitary pads with other materials x 1<br>✓ HIV x 1<br>✓ Keeping long pubic hair x1<br><br>Signs and symptoms<br>✓ Unusual vaginal discharge x 3

✓ Unusual vaginal discharge x 1<br>✓ Bleeding between periods x 1<br>✓ Foul smelling vaginal discharge x 1<br>✓ Abdominal pains x1<br>✓ Lack of symptoms in early stages<br><br>**Sources of information**<br>✓ Clinic x1<br>✓ Other women x 1<br><br>**Usual practice when women notice symptoms**<br>✓ Go to clinic x 4<br>✓ Consult traditional healers x2<br>✓ Self-treatment then clinic x1<br><br>**Knowledge of HPV vaccine**<br>✓ Inadequate knowledge x2<br>✓ Full knowledge x1	✓ Bleeding between periods x1<br>✓ Foul smelling vaginal discharge x 1<br><br>**Sources of information**<br>✓ Clinic x 2<br>✓ Health Promoters x1<br><br>**Usual practice when women notice symptoms**<br>✓ Go to clinic x 3<br>✓ Consult prophets x1<br>✓ Consult traditional healers x 2<br><br>**Knowledge of HPV vaccine**<br>✓ Inadequate knowledge x 4<br>✓ No knowledge x 1<br>**Means of detecting cervical cancer early**<br>✓ VIAC described x1<br>✓ Pap smear described x1	✓ Bleeding between periods x 1<br>✓ Low/ Abdominal pain x2<br><br>**Sources of information**<br>✓ Clinic x 2<br>✓ CHWs x 2<br>✓ Churches x 3<br><br>**Usual practice when women notice symptoms**<br>✓ Go to clinic x 2<br>✓ Consult prophets x 1<br>✓ Consult traditional healers x 1<br>✓ Do nothing<br><br>**Knowledge of HPV vaccine**<br>✓ Inadequate knowledge x2<br>✓ No knowledge x 1<br>**Means of detecting cervical cancer early**<br>✓ VIAC<br>✓ Pap smear	✓ Foul smelling vaginal discharge x 2<br>✓ No symptoms in early stages x 2<br>✓ Severe weight loss x1<br>✓ Pain during sex x 1<br>✓ Pain when passing urine x1<br>✓ Niggling pain in cervix x 1<br><br>**Sources of information**<br>✓ Clinic x1<br>✓ Church x 1<br>✓ CHWs x 1<br>✓ Mobile team x 1<br>✓ IEC materials x1<br>**Usual practice when women notice symptoms**<br>✓ Go to clinic x 2<br>✓ Consult traditional healers x 2<br>✓ Consult friend for advicex1<br>✓ Consult church x 1<br><br>**Knowledge of HPV vaccine**<br>✓ Inadequate knowledge x 6<br><br>**Means of detecting cervical cancer early**<br>✓ Screening x 1	✓ Foul smelling vaginal discharge x 1<br>✓ Low Abdominal pain x1<br>✓ No symptoms in early stages x 1<br>✓ Burning sensation in vagina x 1<br>✓ Bad smell in urine<br>✓ Cervix protruding through vagina x 1<br>**Sources of information**<br>✓ Clinic x 1<br>✓ CHW x 1<br>✓ Mobile team x 1<br><br>**Usual practice when women notice symptoms**<br>✓ Go to clinic x 2<br>✓ Consult CHW<br>✓ Consult traditional healers x 1<br>✓ Consult prophets x 1<br>✓ Do nothing (monitor situation for a few days) x 2<br>**Knowledge of HPV vaccine**<br>✓ Inadequate knowledge x 4<br>✓ No knowledge x 1<br>**Means of detecting cervical cancer early**<br>✓ Screening

**Means of detecting cervical cancer early**<br>✓ VIAC X2<br>✓ Pap smear x1<br>✓ Screening prevents CC<br><br>**Known screening places**<br>✓ Phakama clinic x 2<br>✓ Gwanda Provincial Hospital x 2<br>✓ Private Doctor x1<br>✓ Church coordinated Health Expos once in a while x1<br>**Recommended screening frequency**<br>✓ Incorrect information x1<br><br>**Screening uptake**<br>✓ Uptake is low x 2<br><br>**Screening experiences**<br>✓ Service rendered in a professional manner x 3<br>✓ Procedure was uncomfortable x 1<br><br>**Barriers to screening**<br>✓ Myths and misconceptions x 4	**Known screening places**<br>✓ Phakama clinic x1<br>✓ Gwanda Provincial Hospital x 1<br><br>**Recommended screening frequency**<br>✓ Incorrect information x 2<br><br>**Screening uptake**<br>✓ Few women have been screened x 2<br>**Screening experiences**<br>✓ Service rendered in a professional manner x 2<br><br>**Barriers to screening**<br>✓ Myths and misconceptions x 3<br>✓ Fear of a cancer diagnosis x 1<br><br>✓ Modesty issues x 1	**Known screening places**<br>✓ Gwanda Provincial Hospital x 1<br>✓ Sisters clinic x 1<br>✓ Mobile clinic x 1<br><br>**Recommended screening frequency**<br>✓ Incorrect information x1<br><br>**Screening uptake**<br>✓ Very few women have been screened x1<br>**Screening experiences**<br>✓ Service rendered in a professional manner x 2<br>✓ Information not individualised x 1<br>✓ Procedure was uncomfortable x 1<br>**Barriers to screening**<br>✓ Inconsistent visits by Mobile team x 1<br>✓ Mobile team gives priority of HIV +ve women x 4	**Known screening places**<br>✓ Gwanda Provincial Hospital x 1<br>✓ Manama Mission Hospital x 1<br>✓ Mobile clinic x 2<br><br>**Recommended screening frequency**<br>✓ Incorrect information x 1<br><br>**Screening uptake**<br>✓ Few women have been screened x 1<br>**Screening experiences**<br>✓ Service rendered in a professional manner x 2<br><br>**Barriers to screening**<br>✓ Inconsistent visits by Mobile team x 1<br>✓ Mobile team gives priority of HIV +ve women x 3	**Known screening places**<br>✓ Gwanda Provincial Hospital x 2<br>✓ Mobile clinic x 2<br><br>**Recommended screening frequency**<br>✓ Inadequate information on screening frequency<br>✓ No knowledge of screening frequency x 10<br>**Screening uptake**<br>✓ Few women have been screened x 2<br><br>**Barriers to screening**<br>✓ Inconsistent visits by Mobile team x 2<br>✓ Lack of money x 7<br>✓ Modesty issues x 4<br>✓ Lack of partner supportx1

✓ Negative staff attitudes x 4<br>✓ Religious beliefs x2<br>✓ Socio-cultural beliefs x2<br>✓ Modesty issues x2<br>✓ Gender of person screening x2<br>✓ Long waiting periods x2<br>✓ Questions asked too personal x 2<br>✓ Lack of effective education programmes in health facilities x1<br>✓ Fear of stigma and discrimination x 2<br>✓ Lack of adequate knowledge x1<br>✓ Procedure is painful x 1<br>✓ Disclosure issues x1<br>✓ Lack of partner support x1<br>✓ Nurses lack confidentiality x 1<br>✓ Women are turned away x1<br>✓ Lack of confidence in health services x1<br>**Recommendations to improve screening rates**<br>✓ Intensify education on cervical cancer x 3<br>✓ Introduce mobile clinics x1	✓ Fear of stigma and discrimination x 1<br>✓ Lack of adequate knowledge x 1<br>✓ Procedure is painful x2<br>✓ Fear of HIV test x1<br>✓ Lack of partner support x1<br>✓ Nurses lack confidentiality x 1<br>✓ Lack of confidence in health services x1<br>**Recommendations to improve screening rates**<br>✓ Intensify education on cervical cancer and screening x 2<br>✓ Organise workshops for women x 2<br>✓ Nurses to behave in a professional manner x 1	✓ Myths & misconceptions x3<br>✓ Lack of money x 3<br>✓ Apathy x 1<br>✓ Fear of a cancer diagnosis x 1<br>✓ Lack of effective education programmes in health facilities x 2<br>✓ Fear of stigma and discrimination x 2<br>✓ Lack of adequate knowledge x 1<br>✓ Procedure is painful x 1<br>✓ Disclosure issues x1<br>✓ Lack of partner support x3<br>✓ Socio-cultural beliefs x1<br>✓ Religious beliefs x1<br>**Recommendations to improve screening rates**<br>✓ Provide service at local clinic x 7<br>✓ Provide more frequent mobile screening services x 2<br>✓ Mobile service to provide service to all willing x 3	✓ Myths and misconceptions x 6<br>✓ Lack of money x 5<br>✓ Religious beliefs x 2<br>✓ Socio-cultural beliefs x 2<br>✓ Lack of partner support x3<br>✓ Lack of adequate knowledge x 2<br>✓ Lack of confidence in health services x1<br>✓ Screening not done during menses x1<br>✓ Peer influence x 2<br>✓ Women turned away x3<br>**Recommendations to improve screening rates**<br>✓ Provide more frequent mobile screening services x 1<br>✓ Mobile service to provide service to all willing x 2<br>✓ Intensify education on cervical cancer and screening x 6	✓ Fear of a cancer diagnosis x 1<br>✓ Procedure is painful x 1<br>✓ Lack of adequate knowledge x1<br>✓ Lack of confidence in hospital services x 1<br>✓ Myths and misconceptions x1<br>**Recommendations to improve screening rates**<br>✓ Provide service at local clinic x 1<br>✓ Provide more frequent mobile screening services x 2<br>✓ Male involvement x 3

✓ Nurses to respect client's privacy x 1<br>✓ Review screening questions x 1<br>✓ Use a screening method which does not involve exposure of private parts x 1<br>✓ Use churches as education platforms x 3<br>✓ Have IEC materials written in local languages x 1<br>✓ Reduce waiting period by having separate queues for each service x1	✓ Show women someone with cervical cancer x 1<br>✓ Use a screening method which does not involve exposure of private parts x 1<br>✓ Use churches as education platforms x 1<br>✓ Involve religious leaders in programming education on CC x 1<br>✓ Door to door education campaigns by Health Promoters x 1	✓ Intensify education on cervical cancer and screening x 3<br>✓ Use innovative education strategies x1<br>✓ Periodic mass education campaigns x 1<br>✓ Male involvement x 1<br>✓ Individualised post screening education x1	✓ Education should come first before call for screening x 1<br>✓ Find innovative ways of giving education x 3<br>✓ Male involvement x2<br>✓ Community involvement x 1<br>✓ Introduce programmes which focus on male cancers x 1	✓ Intensify education on cervical cancer and screening x 1<br>✓ Use innovative education strategies x1<br>✓ Community involvement x 1<br>✓ Involve community leaders in educating men x 1

EMERGING THEMES BASED ON THE THEORETICAL FRAMEWORK

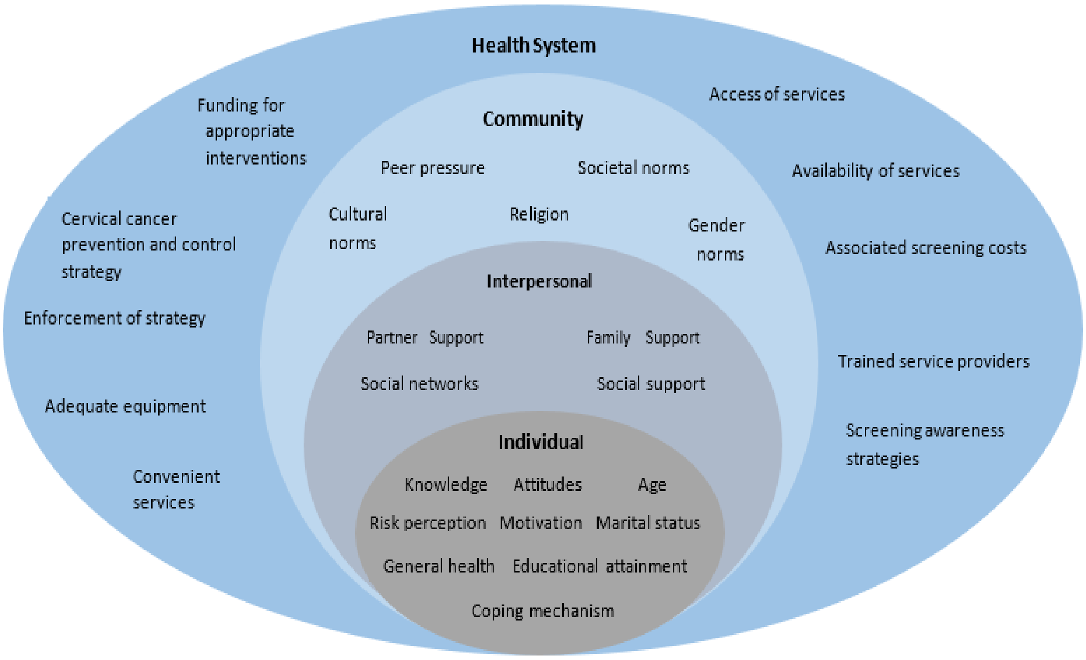

**Superordinate theme**	**Subordinate themes**
Individual factors	✓ Financial Constraints: Lack of money for transport and treatment<br>✓ Modesty issues: Embarrassment at exposing private parts<br>✓ Lack of adequate knowledge on cervical cancer and screening<br>✓ Lack of confidence in health services<br>✓ Fear of pain during procedure<br>✓ Fear of a cancer diagnosis (screening outcome)<br>✓ Apathy<br>✓ Fear of HIV test
Interpersonal factors	✓ Lack of partner support
Community factors	✓ Myths and misconceptions<br>✓ Religious beliefs<br>✓ Socio-cultural beliefs<br>✓ Stigma and discrimination<br>✓ Peer influence
Health System factors	✓ Screening services not provided at rural primary health facilities<br>✓ Mobile screening team gives priority to women on ART<br>✓ Inconsistent visits to Rural Health Centers by Mobile Clinic<br>✓ Negative staff attitudes<br>✓ Women are turned away<br>✓ Lack of effective health education programmes<br>✓ Gender of services provider<br>✓ Long waiting periods<br>✓ Sexual history asked is too personal<br>✓ Nurses lack confidentiality<br>✓ Screening not done during menses<br>✓ Method of screening inappropriate

Objective 5:

To examine existing strengths and facilitators that could be incorporated into the cervical cancer screening programme in Gwanda district

1. Cervical cancer known to be a disease which kills
2. Personal experience with cervical cancer
3. Awareness of cervical cancer screening services
4. Willingness to be screened
5. On-going health education strategies by institution and community based health workers
6. Many women attend church services
