# Supplementary material for: Women and health providers’ perspectives on male support for cervical cancer screening in Gwanda district, Zimbabwe
Source: PLoS One. 2023 Oct 12;18(10):e0282931. doi: 10.1371/journal.pone.0282931 (PMC10569579; doi:10.1371/journal.pone.0282931)
Supplement: S5 File — (PDF) [file pone.0282931.s005.pdf]

IN-DEPTH INTERVIEW GUIDE

Barriers to cervical cancer screening in Gwanda district, Zimbabwe: A mixed method analysis

<u>Introduction</u>

My name ………, a PhD student from ……. University. I would like to ask you some questions about cervical cancer and screening practices among women in Gwanda district. As you will recall the purpose of the study is to identify barriers that prevent women in the district from accessing screening services. Your opinion is very important as it could help find ways to address those barriers to improve the cervical cancer screening programme in the future.

Your participation is completely voluntary, and you can choose not to participate in part or at all in the project. You may refuse to answer any question you feel uncomfortable with and you can withdraw at any stage without being penalised or disadvantaged in any way. Any information that you provide is strictly confidential. Your privacy will be protected, and full steps will be taken to ensure anonymity. Whatever you say will not be linked to your name so feel free to express your honest opinion and make suggestions. The discussion should take about 45 minutes.

I would like to record the interview session so that I do not lose any important information that will come up during the session. Please speak loudly so that I will not miss out on anything you say. I want to assure you that the recording will not be used for any other purpose other than this study and will not be accessible to anyone else other than the research team. You can verify your comments and responses after the recording before the final inclusion. I will transcribe all the information from the recorder and the audio-recordings will be destroyed at the end of the study. You may let me know if you do not want to be audio recorded. If that is the case, I will have to take notes of all that you will say.

Thank you for your willingness to participate in this study. Do you have any questions or concerns before we begin?

Personal Information

Participant ID ____

Site ID ____

Work position ____

Interview Date ____

Time started ____

Time completed ____

1. Could you tell me about your experience in the cervical cancer screening programme?

- What is your position within the health system?
- How long have you been involved in the cervical cancer screening programme?
- What are your responsibilities in relation to cervical cancer screening services?
- Have you received training on cervical cancer screening?

2. What are your views on cervical cancer in relation to this community?

- Cervical cancer prevalence in the district
- Community knowledge and perceptions about cervical cancer
- Women's knowledge on cervical cancer prevention

3. What cervical cancer screening models are available in the district?

4. What are your views on women's behaviours in relation to screening?

- Prevalence of screening
- Women's awareness of the screening services available in the district
- Perceptions about screening

5. Could you tell me about the general practices relating to cervical cancer screening in the district?

- National cervical cancer prevention and control guidelines
- Feasibility of implementing the policy
- Institutional cervical cancer screening standard operational procedures
- Strategies used to reach and motivate eligible women for cervical cancer screening

6. What could be the reasons that hinder women in the district from utilising cervical cancer screening services?

- Health system factors
    - Community health education on cervical cancer and screening

- Access to screening services
- Health system capacity to carry out screening
- Who provides VIAC screening? Follow up and treatment of abnormal conditions
- Cultural appropriateness of screening test for clients

➢ Individual factors
- Knowledge
- Attitudes
- Beliefs
- Risk perception
- Nature of screening test

➢ Socio-cultural factors
- Social stigma
- Religion
- Norms

➢ Interpersonal factors
- Partner support: psychological, financial, HPV prevention support
- Family support
- Peer pressure
- Social networks

7. How do you think these barriers can be overcome?

➢ Probe on existing strengths and facilitators that can be incorporated into the programme

Thank you for participating in this study. Is there anything else you would want to comment on?
