# Supplementary material for: Women and health providers’ perspectives on male support for cervical cancer screening in Gwanda district, Zimbabwe
Source: PLoS One. 2023 Oct 12;18(10):e0282931. doi: 10.1371/journal.pone.0282931 (PMC10569579; doi:10.1371/journal.pone.0282931)
Supplement: S6 File — (PDF) [file pone.0282931.s006.pdf]

IDI THEMATIC ANALYSIS

1DI 1
**Interviewer:** Thank you for agreeing to participate in this interview. The goal of the study is to get the views of health service providers on the cervical cancer screening programme in the district with the view of improving access of the service by all women who require screening.<br>**Participant**: Its ok<br>**Interviewer:** Please explain to me how you are involved in the cervical cancer screening programme<br>**Participant**: The cervical cancer programme; I am a GMO, Government Medical Officer under Obs and Gynae at Gwanda Provincial Hospital. The sisters perform VIAC. VIAC is visual inspection under acetic acid for screening of the cervix. So those who are positive are referred for LEEPs. If there's precancerous lesions we do a LEEP to remove the lesion and send a specimen for histology and that is my participation in the screening programme. And when we get opportunities, we have meetings to do quality control of the VIAC itself whether the images can be said to be positive or negative with sisters here and doctors who are under Obs and Gynae participating and any other doctor and nurse who wishes to participate. VIAC was pioneered by UBH, United Bulawayo Hospitals. The Obs and Gynae there are pioneers and spearheading the programme there in Bulawayo. They have a very active programme there. I had the opportunity to do my internship there and was exposed to what VIAC is all about. So, the sisters are the ones who do the actual VIAC and then they take images with the training that they have received, the sisters decide whether it's a positive or a negative. So VIAC doesn't find cervical cancer but detects precancerous lesions. If you find cancer, there is no need to for you to now do a VIAC, you can see that its suspicious of cancer then you take a biopsy. So, for those who have precancerous lesions, they do interventions. If it's a small lesion, you can do cryotherapy or you can do ablation, either one of those then review the patient in 3 – 6 months to see if there is resolution or progression of the underlying pathology that was found. So, after the sisters have done the VIAC they assign the appropriate result whether negative or positive. Sometimes there are lesions that are not advanced which might not need intervention then, they may need a review. If it is a precancerous lesion you can have resolution. If it's not yet that bad after 6 months you may find that the lesion has just resolved. Others are referred but most are given treatment; cryotherapy, ablation or LEEP. If it's now at an advanced stage, you can do a hysterectomy to remove the uterus if the patient has fulfilled their reproductive ambitions.<br>**Interviewer:** Thank you for the detailed information, may be can we move on and you tell me how many doctors are trained in VIAC procedures.<br>**Participant:** The people who do VIAC are the nurses. Doctors are used in the programme to do quality control just to see if the images which will have been assigned as positive or negative are really that. Because the cervix is a dynamic organ which changes with age and also depending on the period of the menstrual cycle. So sometimes you may see something and think it's positive when it's not so those quality control programmes that's where the doctors come in. So, doctors are not hands on in doing the actual VIAC, we do quality control and the interventions like ablation and LEEP which doctors need to do.<br>**Interviewer: O**k, thank you. So how many doctors are involved in doing those interventions and how often do you have these quality meetings.<br>**Participant:** Its 2 doctors and recently we now have a Consultant Obs and Gynae Dr…… He is on leave, but you should get hold of his number and interview him as well because he also has vast experience from UBH. Previously a consultant used to come from UBH every month to see the lined-up cases but now we have our own right here. We usually try to do the quality control meetings weekly but with COVID it has been less frequent, about monthly.<br>**Interviewer:** How prevalent is cervical cancer in the district?<br>**Participant:** I do not have the data off head because I am still new in the district. But you can get the data from the VIAC clinic although those won't be the statistics for the whole district. You can also check the national statistics; they won't be very different from Gwanda.

Interviewer: In your own view, how knowledgeable is the Gwanda community on cervical cancer?
Participant: From my opinion, they are really not that knowledgeable because you talk to patients, for example a 26-year-old and you find that most of the women have never heard about VIAC which means they have no knowledge on that, so I think there is still much to be done. The problem comes because VIAC is only done in Gwanda. If this programme can be rolled out to every clinic in the district. Phakama (urban council clinic) now has a VIAC clinic but if everyone had the knowledge and were to come here, you could only imagine the disaster. Because us we are a provincial hospital, but we also almost function as a district hospital because there is no district hospital in Gwanda so if the whole district and the whole province comes here, we would just be overwhelmed. So, we just need to have more screening sites. Screening resources and personnel are needed but we have a challenge with nurses, the remuneration. Like here nurses who are doing VIAC also have their other day to day duties, they have to see ANC patients and all that. So VIAC is just an addition, more work to be done on top of what she is doing which is already too much because they are under staffed already, you are now telling them to do VIAC, you don't put people specifically for it and you do not remunerate them for the additional things that they are doing, you will not have it done effectively. That's the problem, we just want people to push programmes, but we don't want to add remuneration. They won't bother educating women because it will mean more work for them. For example, in UBH, VIAC is a separate entity from ANC and Obs and Gynae. The people who are in VIAC do VIAC. They come in and do VIAC and when they are done, they go home. If people are really concerned about VIAC, they should really consider incentives for the nurses. If someone is not motivated and are overwhelmed with many other duties, their level of proficiency may not improve. Nurses may be there, but their minds are not in it, nothing will materialise.
Interviewer: What guidelines are available for the management of the VIAC programme?
Participant: We have a book, the national strategic document on VIAC although Harare prefers the Pap Smear. The Southern part of the country has no resources to do Pap smears therefore we cannot talk much about it.
Interviewer: You mentioned that the level of knowledge on cervical cancer and screening is poor among the community …….
Participant: Interrupts……Yes, and it's just that even if you push the knowledge to the people, we might not be able to accommodate them with the 2 VIAC clinics that we have. If you are going to do an intervention to educate people about VIAC and your clinic can only take 30 people a day and 500 people come, its unethical because you will have to turn them back without proving the service.
Interviewer: May I continue on my previous question where I wanted to ask what strategies you use to sensitive and educate the communities on VIAC so that more women may come for the service?
Participant: Laughs…. that you can ask the sisters.
Interviewer: As we were talking, you have said a lot about some of the factors which contribute to low uptake of screening by women, would you like to add more on that?
Participant: They don't know, and some are afraid of VIAC. Our people are very spiritual, and they are human beings. There are people who are saying 'I would rather not know that I have cancer so it's better not to screen'. It's now up to us as health professionals to educate them, but I will repeat that there is a disconnect between the Ministry of Health and its staff. You will not get a nurse going around telling people about VIAC out of their own sheer will to get more work on their shoulders when they have bread and butter issues to worry about. You want them to be pushing the agenda, but no, they will only tell their relatives and friends. Remunerate them properly to get them motivated. The programmes take away your time.
Interviewer: What recommendations do you have for the improvement of the VIAC programme so that more women have access to it?
Participant: Those doing VIAC should get regular training. Adequate equipment has to be provided. They get incentives, a person should get something for what they are doing. Address the issue of poverty because there is a correlation between poverty and health seeking behaviours. Poverty stricken people will not bother about preventive health services because they have more pressing

issues like how they are going to get food to put on their tables. If poverty is addressed, then screening services may improve.

Interviewer: Would there be something else you would like to add?

Participant: The most important thing is that if the VIAC programme is not properly financed as is the case now, it is bound to fail. More funding should be allocated for the programme. I would encourage you to try and meet Dr Gororo. He will add much value to your study.

Interviewer: Thank you for sparing your time to participate in this study. The information you provided has given much insight into the factors influencing the low uptake of cervical cancer screening in the district.

Participant: You are welcome

1DI 2

Interviewer: Thank you for allowing me to interview you while you are on leave. The focus of the study is to identify the barriers to cervical cancer screening in Gwanda district from the perspective of both the recipients of the service and the service providers.

Participant: Interesting. Please go ahead.

Interviewer: Would you please tell me your experiences in relation to cervical cancer in Gwanda District and the screening programmes, its prevalence, the knowledge women have on the programme, and what the barriers to screening could be, and the recommendations which you may have to improve access of the programme by the women.

Participant: Umm this was, cervical cancer screening in Gwanda District and in Matabeleland South Province as a whole is mainly done with VIAC- Visual Inspection with Acetic Acid and Cervicography. I myself am a specialist, consultant Obstetrician and Gynaecologist for the Province. My duty maybe is to oversee most of the programmes and mentor the units which are offering the programme. And most of the support that we have is from our partners. In terms of prevalence of cervical cancer in general I don't have specific statistics. If I had known I would have prepared them for you. But in general, all I can say is that the burden is quite significant both for the cervical cancer itself and for the precancerous conditions and it's quite a significant burden within the district and also within the province. Probably for me it's very easy to keep referring to the province because the clients that we see are from all over the province and not just from Gwanda district alone. So, the prevalence is quite significant.

Interviewer: Ok.

Participant: In terms of availability of screening services, when it comes to Gwanda district per se, currently we have 2 units that offer cervical cancer screening services that I am aware of. These are Gwanda Provincial Hospital and Phakama Clinic. These are the areas in Gwanda district that are offering cervical cancer screening. At Gwanda Provincial Hospital we had opened another unit, so Gwanda Provincial Hospital has 2 separate units within the same Hospital. We have 1 unit in OI (Opportunistic Infections) Department, that's the one located in Outpatients, then we have the VIAC Clinic in the Antenatal Care Department. The reason why we opened another unit in OI Department is our realisation that people living with HIV are a special group of people when it comes to cervical cancer. The rate of precursor lesions progressing into cervical cancer is a bitter faster when we look into HIV positive patients compared to people who do not have HIV. And even if you look at the screening itself, people living with HIV need more frequent screening whereas HIV negative clients can be screened after 3 years. So, we thought that if we have a unit in the OI department itself it would be easy to capture those clients when they come to collect their ARVs, that's the reason why we opened another unit there, though of course we have challenges that we faced after opening the unit. One of the challenges that we faced was the camera that we use for VIAC. That camera is one for Gwanda Hospital. In fact, there are 2 cameras, but the issue is the lenses that are to be attached to the camera. So, we realised that ultimately, it's one camera that has to be shared between the 2 units so its ultimately the same numbers of clients that we are able to screen because its 2 units but in brackets 1 unit because it can only be one unit functioning per given time. So, that's

one challenge we faced as well as when COVID-19 came a lot of things closed down that also included our VIAC unit they were also slowed down since the era of COVID-19. Including the clients that we had lined up for treatment, clients had been booked for treatment. When COVID-19 came, all those things stopped. We tried to compensate by doing treatment at outreach centres, but we didn't manage to clear the burden. We still have some treatment outreach lined up for next week so that we clear the burden and go back to our normal routine. So COVID-19 should I say brought its own unique challenges.
Interviewer: What other challenges do you face apart those related to COVID-19?
Participant: One of the challenges we face is inadequacy of screening services themselves. The district is too large to have only 2 units to screen. This is not only for the district alone but for the entire Province.
Interviewer: OK
Participant: It's not adequate to have only 2 screening units. Even the numbers that we screen per day in general they are quite too high for 2 units. And that brings a challenge in terms of accessibility of services because it means that even gogo from rural areas of Gwanda has to come to Gwanda town to be screened. The challenge now is that in the screening for cervical cancer, we want to pick it before it becomes cancer. For someone to look for bus fare for something which does not cause pain to them is not likely to happen. So, accessibility of services becomes a challenge because of inadequate number of units that are offering services. My belief is that every rural hospital, every rural clinic**,** every clinic in the urban area, everywhere where there is a clinic, there should be VIAC trained nurses and cervical cancer screening should be offered there. Every place where there is an OI Clinic, VIAC should also be offered. VIAC is easier to establish if more funds could be allocated for the programme. VIAC is less costly to run which I think that should be an area to look into so that people have access to services.
Interviewer: Would you please comment on the knowledge women have on VIAC?
Participant: I think people who have knowledge on VIAC are very very few compared to the number of people who need to be screened for VIAC. If you look at the general statistics of clients that we see at the Hospital, it's a very small fraction that will be coming for the second or third VIAC. The majority are clients that are coming in for the first time. That on its own shows you that very few people are having access and that people are not having information on the frequency of screening. Even for those who are screened and those who have information, the information is not adequate for them to know that one screening is not good for life, they need to be screened again regularly. And even if you look generally at the number of our clients who are actually diagnosed with cervical cancer the majority of them have never had screening. You see someone coming in their 50's for screening for the first time and if you ask them, they will tell you they had never known there is something like this that exists. So, I think that the knowledge gap is very wide. Knowledge on cervical cancer is not what it's supposed to be considering the high burden of the disease that we have.
Interviewer: In June 2019 when I collected quantitative data it was just Gwanda Provincial Hospital screening and now there is an addition of Phakama Clinic also offering VIAC screening. I have been to the Opportunistic Infections Clinic (OIC) and I am surprised that there was no mention of the unit by the staff there.
Participant: I think it's because of that issue I mentioned of camera shifting. You see we do not have both units operating at the same time. Also, the other challenge is the COVID-19 that affected us and the issue of shortage of staff. So, we had to prioritise on one unit to be functional and we chose the one at the OIC. We really need to put emphasis on OIC also if you look at our strategy as well, it's rare for us to have a patient with cervical cancer who is HIV negative. Most of our patients that are diagnosed with cervical cancer are HIV positive. So, that group is actually very significant.
Interviewer: So, in terms of human resources capacity, are you capacitated enough to run both units, not regarding the equipment which has to be shared by the 2 units?
Participant: In terms of human resources now I can say yes, we are capacitated because what we have started doing is to train our own VIAC nurses, that's what we have started to do. So, recently

we trained 4 nurses for that VIAC which brings the number of trained VIAC nurses to 7 apart from the one seconded by OPHID. And next week start on Monday we are going to have another VIAC training again. Our thinking is to rather have an excess of nurses trained in VIAC. They can still be shifted to other departments than fail to screen because we have run out of sisters that can screen, and also to curb the brain drain that is affecting our health system by training more.
Interviewer: Are the Rural Health Center nurses being included in that training?
Participant: Unfortunately, at this point no, because the support that we have to train is from partners. So, as you know when you are funded by partners you have to schedule according to their programme. And the reasons why funders didn't get into training Rural Health Clinic staff and other hospitals in the district is the fact that there is no equipment for the Rural Health Clinics. So, for them to train people that are then going to go back and not practice is a waste and not wise in terms of resources. So, they are saying if the unit is already there then they can train the people. So, the partners happy are because we are now doing the training in our province and not relying on Bulawayo. And there are other districts in the province already lined up to which we will go to train after this training. The other thing that our partners also want is that people should be trained at their places of work so that they are exposed to the things they will remain using which they feel will bring a better uptake and outcome at the end of the day.
Interviewer: Can we move on to the concern of primary health centres that outreach services provide screening services only to a specific age group 25-49 years and just to HIV positive clients. Is there a way this could be addressed so that all potential women receive the service, or is the issue of implementing partners again?
Participant: One of the things that I feel is important is that we appreciate that there are a lot of things which we have achieved which we would not have managed to achieve without them. Unfortunately, partners have their requisites and the way they want things to be done. Although there is a gap in the service, if someone is giving you something you can't dictate. So, I think it's up to us, by us I mean me being part of the government and Ministry of Health to say the burden of cervical cancer is too high to be depending on mobile clinics. Mobile clinics are just doing what they can now but that's not the best. The best is to have our Rural Clinics provide VIAC screening then we don't need to have patients that depend on mobile clinics that come when, I don't know to be screened. That's the way to go Rural Health Centres should have VIAC units. Anyone who want to be screened should be able to walk in any day they want to be screened, every clinic that offers OIC services should have a VIAC unit there so that there is no dependence on partners and the mobile clinics. The mobile clinics should be there to complement the existing services. Depending on a partner to come through mobile clinics may not do much to address the current cervical cancer burden.
Interviewer: In your own opinion, how far are we from reaching the cervical cancer prevention and control targets for 2020 according to our 2016-2020 strategy document?
Participant: I personally feel we could be still a bit far because of those issues of unavailability of services, issues of facilities that are actually offering the service. We are still very very far. As I am looking at the population of eligible women in the district, and there are still 2 places offering screening. How are all those people in areas distant from Gwanda town going to access the services? Should all those people come to Gwanda town just for screening? So, I feel we are still quite far. Also, the reason why I am saying that is because when we look at statistics where we still have the majority of the people being first timers, it means the service itself is not where it is supposed to be. When the availability of the service is now good, we should start seeing repeat clients increasing - clients that have been screened before. That should show that we have screened a lot of people before, the knowledge is now widespread among the people and people have the knowledge that they have to come for their 3 yearly cervical cancer screens. But when every day we are still seeing women coming for the first time and half of the clients we screen are not people who would have specifically come for VIAC itself but are people who would have come to Gynae Clinics, then referred to be screened. So, if this patient did not have a problem that warranted them to be seen at Gynae

Clinic, they were not going to be screened, you see. So, we want a point whereby our clinics are fully operational, and people just walk in for screening, not people who have been referred because they have other problems. Even in my private rooms here I have set up a VIAC unit, but of course not everyone can come because of the charge. But all clients we screen are also first timers. So, it cuts across from clients that are in the rural areas to clients that are in town and affluent. You will see that the knowledge is generally too little and not many women have been screened.
Interviewer: So, is there a strategy that can be used to motivate more women for screening? Like you have said clients go maybe because they have been to the Gynae Clinic and referred? Some women could be coming to the hospital and may not even be aware that there are screening services offered.
Participant: One of the reasons why we have opened a screening unit at OIC Clinic is that the health provider should initiate screening. Each client that comes to collect medication should be offered VIAC. But in order to make a great impact within the province and even in the country, I go to the same point again, let's have VIAC offered at every clinic. Let's have involvement of community nurses to spread the message. Let's have the community health workers told about these things to tell everyone in the village that we are now offering this at the clinic. All the mothers that are coming for the 6th week postnatal clinic, let them be told and offered VIAC. This can be done.
Interviewer: How easy is it to follow up the VIAC positive cases, maybe before the COVID-19 era? Has it been easy to follow them up to come for treatment?
Participants: Yes. That area is one of the things that have quite a very good strength. The follow up is quite good. In those registers the clients leave their telephone numbers. And one thing that is good about VIAC, someone gets their results there and there. You get screened, you come out knowing, are you negative, are you positive or is the result suspicious of cancer. So, you are told that you are supposed to come on this date for treatment. So, in most cases it's very very easy because the moment you test positive, you are booked there and then for LEEP. In fact, before the COVID-19 era every Friday we were doing LEEPs at the hospital. So, if someone tests positive, they are booked for LEEP on Friday that same week. Those who don't come the phone number is there, we call them to ask why they didn't come. So, the follow up is quite easy and it's quite good. Even for those on whom we would have done LEEP, when the histology results come, we go through every result, analyse every result, what lesions is it, are the margins free or they are involved. If the margins are involved, we phone them, we recall them, discuss the results with them and do another LEEP and some opt for hysterectomy. So, the follow up of those that are screened is very good.
Interviewer: Is treatment like LEEP also free like VIAC screening?
Participant: The treatment for LEEP is in 2 forms. There is a small fee which is paid to the accounts dept if the LEEP is done under the hospital. Then there is the LEEP which is paid for by partners from clients seen at outreach clinics that is those which are done under LEEP camps from morning to evening. Those ones partners pay for. Clients don't pay anything.
Interviewer: Thank you so much for a very informative discussion. Is there anything else you would like to comment on that we didn't touch on?
Participant: In general, my point of view is that things that are going to make a difference in terms of reducing the burden of cervical cancer are low cost high impact initiatives. It's not doing fancy things, they are good, and we also have to move with everyone else within the globe, but when we come to making an impact to go deep in the rural areas, it's low-cost high impact interventions which will work, and I think VIAC is one of them. VIAC is not very expensive to set up. I talk from experience because I have also set up a personal VIAC unit. So, if those things are done throughout the country, 20-30 years from now, the burden of cervical cancer should have been reduced. Of course, combined with other things like the HPV vaccination that is going on. But since the HPV vaccination is targeting a population that is still young, it will take us time to see its impact. But for the population that is now in the adulthood, our only hope is to screen and to screen every woman. That way we would be able to make a difference. I tell you if the screening is done adequately, because our general pathophysiology of cervical cancer is that it takes 10 to 15 to 20 years from a premalignant

condition to become cancerous; so, if for the next 10,15,20 years every woman is screened regularly, I tell you in the next 25 years or so, the burden of cervical cancer will be drastically reduced.
Interviewer: Thank you so much for the fruitful discussion. May I come back to you during the writing of my report if I need to clarify some issues.
Participant: You are welcome anytime. Just send me an email.

1DI 3

Interviewer: Good afternoon. Thank you for your willingness to participate in this study despite your busy schedule. I will ask questions pertaining to the cervical cancer screening services in the district and ask that you be as objective as possible as the findings could help improve delivery of the programme.
Participant: Thank you.
Interviewer: How involved are you in the cervical cancer screening programme?
Participant: As a nurse administrator, I am responsible for ensuring the smooth running of the VIAC department. I am also responsible for supervising the staff that is there so that they adhere to the national screening guidelines in terms of management of patients in the VIAC department. I am also responsible for monitoring the records that are developed in that department to ensure that they develop reports and also compile statistics and reports accordingly depending on the levels that are available so that the Ministry can make informed decisions. And I'm also responsible for ensuring that all patients that access these services are given enough information and ensure availability of sundries for use in the department.
Interviewer: How long have you been working in this institution?
Participant: As a manager?
Interviewer: Yes
Participant: As a manager at this level I started in December 2014.
Interviewer: Have you received any training on VIAC?
Participant: No, personally I haven't had any training on that.
Interviewer: How would you rate the prevalence of cervical cancer in this district?
Participant: I think from the time this programme become active; we have actually observed that there are a lot of patients who present with problems related to cervical cancer. It may be above 15% because from the patients we have observed and from the reports that we see, a lot patients have had problems identified which relate to cervical cancer.
Interviewer: In your opinion, how knowledgeable are the women on cervical cancer including the screening programme?
Participant: I think based on observation, if we look at the uptake of the programme, although not high, it has actually improved so much that we have actually seen a lot of women accessing these services, some coming from other districts. We see a lot of patients coming from other districts from within our provincial constituency. And we have a lot of women who we meet in the community who will ask you about the availability of services in relation to VIAC and what actually happens in terms of the procedure. By so doing you can actually tell that a lot of women have now been sensitised in terms of availability of this service in this institution.
Interviewer: The way I understand you, women are actually asking how the procedure is done?
Participant: Yes
Interviewer: That is good. And what services do you have on offer, how do you deliver the screening services?
Participant: When patients come in, as they get through the consultation area in the out patients department, when they state that they have come for VIAC services, they are sent to the VIAC clinic. In terms of VIAC screening, everything is free, they can now incur other costs if they now have to get treatment and then they can also incur costs like when a specimen is taken. We do not test them at this level in terms of our laboratory services, so they incur costs for the specimen to be taken to the next level for testing. But generally screening is for free and medication is free unless

there are other extra costs. But because we now provide a bit of comprehensive VIAC services, you will find that for other treatment options, they are available within such as cryotherapy and LEEP. This is also complimented by the availability of the doctor who is specialised in Obstetrics and Gynaecology. So now there are reduced costs as patients do not need to be referred to the next level.

Interviewer: Would you explain what guidance you have such as on deciding when women should start screening and how frequent they should be screened.

Participant: We have national guidelines, but the problem that we have is that the nurses who are trained will be in the VIAC department. When clients go there, they can actually explain everything from A-Z on how they will proceed from screening to follow up where they are given reference cards on when they should next come for screening. So, the procedures are there for us to follow as a screening site.

Interviewer: How feasible do you think these guidelines are to implement?

Participant: They are practicable and not challenging because VIAC is a simple procedure. The guidelines actually tell you what to do when a client comes for the initial assessment. Based on what they see during the examination, the guidelines tell you what procedures to follow depending on whether the case is an infection or requires surgical intervention. It's all there. With the availability of the doctor the patients are seen there and there. The doctor determines the next steps that are to be followed. And whenever a procedure needs to be done, the patients are booked and there is a room from which they are done for surgical interventions plus follow up. So, its simplified and straight forward and all takes place in the same department.

Interviewer: You mentioned that the community seems to be aware of cervical cancer as a disease and about screening. What strategies is the district using to disseminate that information?

Participant: What we normally do as the provincial hospital is to liaise with the community district offices who deal directly with rural communities. What we have also done is that during outreach clinics when the district teams go to Rural Health Clinics for other services within the community, we second nurses from VIAC clinic so that they go with them and inform the community about the available screening services so that they can come to access them. And then while we are still at that, within the urban community at times there are sensitisations that are done by the Municipality office with the Health Promoters. They usually move around with a car reporting upcoming programmes including VIAC. And then we also have our OIC (Opportunistic Infections Clinic) clients who are given information on VIAC with the hope that when they go back to their communities, they will also cascade the information down. We operate as a district hospital also, so we see patients from as far as rural communities. And at times we also conduct outreaches with the district office for the purposes of screening women from other institutions within the district. We go as far as Manama (this is a mission hospital) who have selected clinics that they cover. The nurses from Gwanda will get their gadgets and everything and move with the community and sensitise them to say on a particular day you are invited to come and access the VIAC services from the selected health facility.

Interviewer: So, you are saying these outreach services are done by the staff from the Gwanda VIAC clinic?

Participant: Yes, because basically, I am not so sure if they have cascaded the screening service down as yet, but our centre is the initial centre within the province to start doing VIAC. So, our nurses who have been trained are the ones who have been providing mentorship plus outreach services in collaboration with the community district office and partners.

Interviewer: How often are outreaches clinic conducted in the communities?

Participant: It's not really consistent because it is dependent on the availability of vehicles for them to go. Normally the district nursing office has a schedule for community programmes and then asks if a VIAC nurse can join the outreach team.

Interviewer: Ok, so in your opinion, what do you think could be the reasons why women fail to utilise these screening services which are provided for free?

Participant: I think the major reason is financial constraints. If we look at the population that we are servicing, you find that we do get challenges whereby if we say the client should come back for follow up, at times they don't come back. If we call to ask why they did not report for check-up, they will cite reasons due to financial constraints. It is a challenge for them to move from their homes to this institution. And then I think at times we have challenges of resources not being fully available, and disruptions in supply of power for us to ensure that our gadgets are in place and to ensure that we have sterile packs. We used to be challenged by power outages such that at times patients may not be able to access the services. It's better now. But generally, I think it's the socio-economic status of the community although some still by all means come and access the services.
Interviewer: How adequate is the staffing at the VIAC clinic for comprehensive delivery of VIAC services?
Participant: I think in terms of staffing we were very challenged because the same staff are the ones manning the antenatal clinic. But for now, what we have done, we have actually trained extra nurses. I think in the last 4 weeks we trained 4 other nurses so that if the others are not there, there will always be someone on duty but before then staff was a bit low. But we were also being assisted by a non-governmental organisation OPHID. They have a specific nurse that is strictly doing VIAC services. So, from the time this nurse came, the uptake improved, this must be her second year. But as of now they have actually moved her from our VIAC clinic to Phakama clinic. The reason was that Phakama clinic is located within the residential area where they see volumes of patients she has to be there. So that then has prompted us to ask ourselves if this nurse at one point moves out, what will we do? This made us consider training our own nurses so that at least we always have VIAC trained nurses available.
Interviewer: Ok, how do you view the VIAC clinic operational hours considering accessibility of screening by all women?
Participant: Ummmh, I am not sure because the VIAC clinic is closed during the weekend and public holidays. Maybe it's something we can put into perspective and try to analyse because the other issue that we are actually looking at is the availability of people that are there, manning the department. Monday to Friday viz-a-vis the inclusion of other services. But maybe if we have an increased number of these trained nurses, we may actually spread the days to say some can be off beginning of the week so that they can be available over the weekend to cater for those not free during the week. It's something which could be considered.
Interviewer: So, looking at the socio-cultural factors, could any of these factors be contributing towards the decision making for screening by the women?
Participant: Probably to a certain extent. I may not say in relation to VIAC exactly because it's just an observation based on general provision of services. We still have mothers who will come with complicated situations because of socio-economic and cultural issues. So, probably with VIAC we may have those. But I am not really sure that I can say I have really observed such challenges.
Interviewer: From the highlighted challenges, what would you recommend for improving the accessibility of the programme to women?
Participant: I think looking at the size of our district, you will find that it's only the hospital that is providing this VIAC service. So, it infringes on other clients especially from rural areas who are unable to access the service due to issues to do with finance. There are other socio-economic issues that are there that need to be dealt with first. I don't know how the health system can improve the operations so that these women can access the services. May be the Ministry should increase the budget for outreach services so that instead of women coming this side, we can go and get them closer to where they are. Or we can also work with rural health centres. If funds are available, it would actually be better to have VIAC screening at the local clinics so that women are serviced within their local clinics. I think that one can take care of the financial constraints. And, then while we are also still at that, there was a time when we had delays in turnaround times of results or sending specimens from this level to the other level. These specimens were sent to Lancet House, which is a private laboratory, and the results determine the intervention. If the government can make sure

that the local laboratories have the capacity to do the testing, this would reduce the turnaround time so that we have early interventions for these people because at times when we get the results back the patient is no longer available. The process of starting to look for these patients is very challenging because the phone may fail to go through because of network challenges, meanwhile the cancer is progressing. As we are improving the uptake of screening, the government should also improve the provision for testing to cut the turnaround time for early interventions. Whilst we are still on that, maybe there should be a provision to train more nurses and open more screening sites.

Interviewer: Could there be anything else you would like to highlight?

Participant: I am not sure about the extent of the involvement of religion because we have a religious sect in Gwanda South which does not allow its members to access any health services. So, if they are not allowed to have their children immunised, no family planning or even paracetamol for a headache what more of VIAC? This is an important area which has to be explored to help such communities access health services including VIAC. But I don't know how this can be done. It is an area which needs to be further explored because such beliefs are contributory factors to the high incidence of cervical cancer.

Interviewer: Thank you very much for the discussion which will help me in addressing the research question and hopefully contribute towards improved uptake of the cervical cancer services in the district. I will send a summary of the findings at conclusion of the study.

IDI 4

Interviewer: Good morning and thank you for agreeing to participate in this study although my appointment was not with you. Although you have indicated that you are just four months in your current role, I am sure you will assist with the information I need since you are also a community health nurse.

Participant: You are welcome, it's unfortunate that the District Nursing Officer got engaged in another week long programme as well as the senior community nurse.

Interviewer: That is fine. Like I said, the study is meant to evaluate the challenges which women face in accessing cervical cancer screening services in the district with a view of making recommendations for improving the programme if indicated. So, I would like you to tell me about how you are involved in the cervical cancer screening programme in the district?

Participant: Ok, as a community health nurse, my job is to disseminate information. We meet these women during our day to day work like when we are going for EPI (Expanded Programme on Immunisation) services. When we are immunising children, we meet these child bearing women and then we take the opportunity to disseminate information about cervical cancer and its dangers. And, also, we meet men out there on social settings. We sensitise them to understand the dangers of cervical cancer too because my general understanding of this cervical cancer, it takes 2 to tango, there's a man and a woman. Usually, a man will pass this HPV virus to the woman without any knowledge of how it was passed during sexual intercourse so they should know the mode of transmission of the virus that is involved in the causation of cervical cancer. So, we meet men out there, we also give them the relevant cervical cancer information. And, if we have pamphlets, we place them in strategic places. My primary role is that of dissemination of information.

Interviewer: Have you received training in VIAC even from where you have been before you come to Gwanda?

Participant: No, I have not been trained.

Interviewer: What are your views on the community's knowledge about cervical cancer and their awareness of the cervical cancer programme?

Participant: I think the awareness is very low, I have served this district in different capacities. When I worked in the female ward, I discovered that women knew very little about cervical cancer. I witnessed many women who would be admitted in the female with stage 4 cervical cancer. It means they did not know about cervical cancer. Meaning that when they developed the signs and symptoms, they were not aware of what that meant. This is in an indication that the communities

where these women were coming from have no knowledge of cervical cancer. This means we need to raise awareness on cervical cancer and cervical cancer screening in the district.
Interviewer: In your opinion, what are the women's perceptions about screening? How do they accept it?
Participant: Women are forth coming, but there are challenges here and there. Like we need to take the services to them considering that there is no transport from their areas to Gwanda. Especially with the Covid-19 in the picture, there are transport challenges. It's difficult and expensive to move from point A to point B. Services are provided at Gwanda Hospital and Phakama Clinic, but it's not easy for a woman from for example Sengezane to stand up and come to Phakama clinic. So, the VIAC team has to frequently take the services to the community so that people are able to access the services.
Interviewer: Having mentioned about the importance of taking the services to the people, would you care to elaborate what type of screening services the district has to offer currently?
Participant: Apart from offering screening services at the two static sites Gwanda and Phakama, I understand there are other non-governmental organisations that are doing VIAC screening in the rural areas. So, they identify specific sites in the rural communities where the women will go for VIAC screening. There are very few nurses in the district, so with that identified gap, the gynaecologist from the hospital I understand is organising a programme to train more nurses on VIAC. That way maybe there will be frequency in the operation of the outreach clinic to the Rural Health Centres with more trained staff.
Interviewer: I was informed at the provincial hospital that there are outreach services which the VIAC clinic staff do in collaboration with your department. You have mentioned non-governmental organisations. Would you like to elaborate further on this, and also explain what services you provide to communities in which you also request a nurse from the VIAC clinic.
Participant: Yes, we do an integrated approach programme. We go out as a team which has the psychiatric nurse, community nurse, rehabilitation technician, and also the environmental health technician and someone who has experience in VIAC. The primary role is to sensitise the communities through disseminating information. This is different from the VIAC mobile clinic which goes out for the purpose of screening and this service is provided by other organisations. The role of our district team is community mobilisation and information dissemination to lay the ground for the outreach screening team. So, when dates are set, people in that community are already aware of the programme.
Interviewer: I see. How is the uptake of cervical cancer screening in the district?
Participant: At the moment I think we are around 30% coverage, maybe we could reach 40% by the end of the year because we are really speeding up the dissemination of information.
Interviewer: Are you aware of any guidelines for the cervical cancer prevention and control which direct you in the provision of cervical cancer prevention services?
Participant: Yes, we have a national strategy document which address that. It's there somewhere.
Interviewer: What strategies do you use to disseminate information on cervical cancer and screening to reach more women with information since you mentioned that women's knowledge levels are low?
Participant: We encourage the Rural Health Center nurses to give health education to mothers and sensitise them for screening. But I am not sure if they will be included in VIAC training because they need the full information for teaching mothers. The integrated approach to provision of health services which I mentioned started recently; its main objective is to increase awareness to the communities on health programmes, especially the young women. We camp out there for the whole week in those respective areas.
Interviewer: So, when you go out and camp the whole week how do you deliver your services to the communities?
Participant: We cover different components of health such as Family and Child Health services including VIAC. The team picks the difficult to reach areas in different Wards. The programme is

being funded by Matabeleland Aids Council. They provide the technical skills and transport. Today we are actually going out for a week, we are waiting for transport as we speak.
Interviewer: That sounds like another good information dissemination approach. So, with all these efforts, why do you think women are not utilising screening services as much as we expect them to?
Participant: Silence
Interviewer: What challenges could women be facing on accessing screening services? Maybe you could look at the challenges holistically from the individual woman, the community and right up to health system related factors.
Participant: The challenges are two pronged. Looking at the side of the community, the perceptions around cervical cancer screening; there is a need to really sensitise the community so that they have a good understanding of the importance of all this. Sit down with them and explain. On our side we should go to them with the screening services and avoid promising then failing to go. What has sometimes happened is that we group those people out there, then later phone them to say there is no fuel we are unable to come. Next time they won't come. Like what happened last week. Women waiting for us then we fail to go. It's a challenge. And the other thing, generally, issues of reproductive health among people in our communities are sensitive to talk about. When you talk about those things traditionally in our culture, you need to apply a lot of persuasion. This is because examining an African woman sometimes needs a person to be vigilant. You need to talk to them, sensitise them and go back time and again so that they understand. There is need to also show them any visual aids on the importance of cervical screening because they learn faster when you show them something than just talking; if we get even videos and visual aids so that they see exactly what we are talking about.
Interviewer: You talked about involving men, how supportive are the men of their women when it comes to screening?
Participant: The issue of male support is a challenge. One of the factors is with our economic environment. You find that in some areas men go out gold panning with these young women, so it's not easy to reach those we need yet that it is the age group we are targeting. You only find young girls and old people in the homes. The middle aged are out there with partners who are digging gold. The challenge is that people are mobile these days and they want to be incentivised. The good thing is that our Community Health Workers (CHWs) have been given good incentives and I really appreciate the good work they are doing in motivating women. Also having male (CHWs) has helped as they mobilise and teach other men which makes it easier than if they are taught by women CHWs.
Interviewer: The way I understood you, CHWs have been nicely incentivised and that helps in having them do more sensitisation work.
Participant: Yes, they are currently very motivated.
Interviewer: Any socio-cultural factors which might be influencing screening decisions?
Participant: Yes, using community leaders has helped in the positive response to the sensitisation sessions. We do not go straight to the villagers bypassing the influential leaders and community structures. Culturally they are respected and can help move the programmes, starting with the chief, the headman and councillors. And also, the husbands should not be left out because we should not take for granted that women will come without the knowledge of their husbands because they may refuse to allow their wives to attend screening if they have not been informed. So, there is a need to also target men with information in their circles for better acceptance of the screening programme. If you target one side, you lose.
Interviewer: Is there anything else you would like to add to the information you have provided.
Participant: We have said most things.
Interviewer: In a nutshell, what would you want to see changed to facilitate more women to be screened?
Participant: Number one thing is to equip the community with the knowledge in screening benefits so that when we take the service to them, they already understand it. We also need to have adequately screening equipment for the screening centres so that the services become available

anytime. Women should be able to access services at their local facilities without incurring expenses to come to Gwanda. The other issue is to intensify community mobilisation and involve men. We should not take these things for granted even without organising meetings. Find them where they are. Always involve community leaders. When information comes through their leaders, people will understand it better. We should use the language which people understand, including the materials we distribute. Pamphlets are available, but they are not reaching the intended populations. We need to have posters at sites so that people see for themselves what one is talking about.
Interviewer: Is there something else we did not mention which you would like to highlight.
Participant: The problem we have is with continuity. Programmes and interventions come; people take them, then the programme disappears. When it resurfaces, people will not come. There is no continuity of services after you have sensitised the people. What happens after that, communities will not trust the health system. Interventions which are introduced should be continued. There should be continuity of service provision. I am talking about the VIAC outreach programme. When women are screened, those identified as positive should get treatment for free from where they are screened. There is no use to create awareness on the VIAC programme if the service cannot be fully provided. The Ministry should find adequate means of funding programmes so that equipment and all needed resources for it are always available. People who are seen at outreach clinics should be treated there instead of being referred to Gwanda Provincial Hospital for treatment. That's another expense which some women cannot afford, and they are left knowing that they have a problem which they have no means of having it addressed. Sometimes that may develop into invasive cancer because they lack money for transport. That is a weakness. There is no system of monitoring patients who are diagnosed with cervical cancer. Once they are referred to a central hospital they are lost. There is no system compared to patients who has been diagnosed with Tuberculosis. There is notification and there is contact tracing and we know where this patient is but not with cervical cancer. They are lost somewhere, and we don't know what has happened to them.
Interviewer: Thank you for your valid contributions and I hope you will enjoy working in Gwanda district. Your contributions will add to the recommendations for improving the cervical cancer screening programme.
Participant: You are welcome. We also look forward to having more women reached with screening.

1DI 5

Interviewer: Thank you for participating in this interview. I appreciate that you are busy today and will try not to take too much of your time. As I mentioned earlier, we want to assess the performance of the cervical cancer screening programme in view of improving access of the screening service by women. Could you kindly tell me how you are involved in the screening programme.
Participant: I am a VIAC trained nurse in charge of the department which also provides antenatal care services. We screen all mothers who come for VIAC or who are referred from other hospital departments for screening.
Interviewer: Ok, how long have you been involved in the screening programme and would you please share your experiences.
Participant: Since 2016, 4 years.
Interviewer: Please share on the preparedness for screening of staff working in the VIAC Clinic
Participant: All nurses working here have been trained in VIAC. We are four who are trained and if I am not mistaken 3 others were trained in the past 2 weeks although they are not yet working here. So, I think we are now 7 nurses trained in VIAC.
Interviewer: In your opinion, how does the community view cervical cancer and cervical cancer screening in general?
Participant: I think most of them still need more information on cervical cancer screening because the turn up of clients, I don't know whether its economical or what, is not much, especially women coming from the rural areas, people don't know much, and also maybe it's due to the distance

because currently in Gwanda district it's just the hospital doing screening but recently Phakama clinic (urban council clinic) has also started screening but it does not help much because it's still in Gwanda town. But we've got some clinics in the rural areas, but screening is not done there. I don't know whether the information is lacking on the part of the nurses there, but I am also sure it's the transport problem. With Covid there is restriction in movement of buses.
Interviewer: The way I understand you, mothers may not be aware of the cervical cancer screening programme?
Participant: Yes
Interviewer: Ok. So how prevalent do you think cervical cancer is in the district?
Participant: If you are saying cervical cancer, I am not sure because when we screen, we detect precancerous lesions and suspicious cancer and to prove that a patient has cervical cancer we send the specimen for testing and need the results to prove that this one has cancer. We have many cases of VIAC positives. So, are you asking about cervical cancer or precancerous lesions?
Interviewer: I am referring to those with a confirmed diagnosis of cervical cancer.
Participant: Ok, that is complicated because we have to wait for lab results to prove that its cancer but per month, we see about 2 out of every 4 clients who get results suspicious of cancer. These are those before the lab results, ok.
Interviewer: How do you provide screening services in the district?
Participant: What do you mean about that?
Interviewer: Apart from offering screening services at the static sites you mentioned, are there other approaches you use for offering the service?
Participant: We offer screening services at this hospital and we also have outreach services which are sponsored by our partner OPHID. For those sponsored by our partners, they are concentrating on certain types of individuals and not everyone else.
Interviewer: Could you please shade more light on the certain type of individuals.
Participant: HIV positive women, but if they also find HIV negative women at the outreach site, they are also screened but their target is HIV positive women.
Interviewer: How do you get women to come for screening?
Participant: Usually in rural areas the nurses at the clinics are the ones who mobilise the women although there are some who just come on their own when they hear that the outreach team is coming to their area.
Interviewer: In your view, how keen or motivated are the women to get screened?
Participant: Mhh, I think ……I don't know, but usually the ones in the rural areas have interest because when we go for outreach to the rural areas, most women come wanting to be screened even when they have not been invited because they do not meet the criteria of our partner. I think outside town, they are more interested more than the ones in town. I'm not sure if they understand it much better or they are afraid, but the ones in town show less interest. We just give health education and it's for them to decide.
Interviewer: In other words, you are saying the response to screening is better in rural communities than in town?
Participant: Oh yes
Interviewer: Do you have any screening guidelines and if so, how effective are they at making your service delivery efficient?
Participant: We use the national guidelines which tell us which type of women to screen, that is the age group to screen; 18 – 65 years and how often they should be screened, which is 3 years for HIV negative women and yearly for HIV negative women.
Interviewer: How do you reach women who are eligible to motivate them for screening?
Participant: Eh, usually we give health education and it's up to the individual to come forward after hearing about cervical screening. And as I've already said that we have some partners, when it comes to treatment, they pay for the clients' treatment. I think that one has helped some clients come forward because they know that treatment is free so that motivates them.

Interviewer: How do other hospital departments contribute to the provision of screening services?
Participant: Usually in the outpatients department, MCH and OIC (Opportunistic Infections Clinic), health education is given and those in the wards have some information which they got from the OPD.
Interviewer: You have already touched on some issues on why women fail to access cervical cancer screening, would you like to talk more on that?
Participant: In town, locally?
Interviewer: Yes, in town and also pertaining to the whole district.
Participant: I will say it again that I think the issue is on knowledge, there is a knowledge deficit, they are not well versed with cervical cancer and screening also maybe as I have already said that since Gwanda Hospital and Phakama clinic are the only ones which do screening, maybe the challenge with rural women is the funds to come into Gwanda and have these services.
Interviewer: Could there be any socio-cultural issues influencing women's decision to screen?
Participant: Ya, that's a very important point. Gwanda district has some cultures and religions which do not go to hospitals, so definitely those women are not aware of the health services offered in health facilities. The other challenge is that if women are VIAC positive and are treated with cryotherapy, we encourage them to stay for 6 weeks without sex so our men do not want to stay that long without sex and they are the ones who make decisions related to sex, so women will not come for that reason to avoid problems at home.
Interviewer: Could you comment on the health system capacity in the provision of cervical cancer screening services?
Participant: I think also the challenge we have is that there are a few nurses who are trained to offer screening services. So, if one nurse is off sick and another is off duty, there is no one to do the screening.
Interviewer: What are your VIAC clinic operating hours like?
Participant: We are operating from Monday to Friday, 7 – 5.
Interviewer: Ok, does that mean that you are closed on weekends?
Participant: Yes, and on public holidays.
Interviewer: What are the strengths inherent in the health system which could be used to motivate more women to come for screening?
Participant: Silence.. seems to be deep in thought
Interviewer: What factors motivate more women to screen for cervical cancer?
Participant: The strength we now have is that we have recently opened another VIAC clinic in Phakama so women can get the service nearer to where they stay. Another screening unit was also opened at the OIC early this year but unfortunately the same people are the ones who have to operate there such that the 2 hospital units cannot function at the same time. It stopped doing screening when the sister from our partner went to open Phakama VIAC clinic.
Interviewer: What else could encourage more women to come for screening?
Participant: I think what could encourage more women to come is to open VIAC clinics at the Rural Health Centers so that women don't have to travel all the way to Gwanda then spend a lot of time in queues before they are screened.
Interviewer: Ok. On average, how many women are you able to screen in one day?
Participant: Usually we do not take a specific number but take all who come, we usually screen 10-15 if they come, but the challenge is that they are not coming.
Interviewer: How would you like to see the programme improved so that more women can seek screening?
Participant: silence
Interviewer: What recommendations do you have to make the programme more accessible to women?
Participant: I think it should be decentralised to the local clinics, have staff trained there so that screening is done at grassroot level. And also, of course VIAC is free, but some of the treatment is

paid for. So, if they could make everything related to VIAC free including the treatment then more women would be willing to be screened because what is the point of screening when you cannot afford to be treated if you require to be treated?<br>**Interviewer:** Is there anything else you would like to bring to my attention which we have not talked about?<br>**Participant:** I think it is important that as provincial hospital which is a tertiary referral center we have a stand-alone VIAC clinic as a referral unit for other clinics for treatment. But currently we do not have our own stand-alone VIAC clinic so that we provide comprehensive services. Of course, its ok to integrate services, but I think it is also important to have a specific VIAC referral center especially for issues of treatment like cryotherapy and LEEP and also have adequate equipment there. We do cryotherapy in the VIAC clinic but with LEEP currently as I'm saying we do not have space, so we are using the small theatre in the hospital. So, we take our machines to theatre and pack them and carry them back after the procedures, which is a challenge.<br>**Interviewer:** Anything else you would like to add?<br>**Participant:** No<br>**Interviewer:** Thank you again for taking your time to participate in this interview. I will share the findings with the hospital authorities at the end of the study.
**1DI 6**
**Interviewer:** Thank you for agreeing to participate in this interview. Like I said, I want to get your views on the VIAC programme in the district so please be as objective as possible in the information you give. It will not be used for any other purpose except for that related to the study.<br>**Participant:** OK<br>**Interviewer:** Could you kindly explain to me how you are involved in the cervical cancer screening programme.<br>**Interviewer:** I am a VIAC trained nurse. I am doing cervical cancer screening on mothers who are sexually active, all the childbearing mothers – HIV positive and HIV negative. We screen them on a daily basis.<br>**Interviewer:** How long have you been involved in this programme?<br>**Participant:** 2 years<br>**Interviewer:** In your opinion, how prevalent is cervical cancer in the district and how would you rate the knowledge that the community has on the disease and on the preventive measures which are available?<br>**Participant:** I would say the disease is quite prevalent, but we are doing sensitisation. There are some people who are not aware of this cancer. There are also some people who are ignorant. They don't want that procedure to be done to them like they are being told bad things by those individuals who have done it. They are saying they don't want things inserted in their private parts so there are people who still need education. I think we need to do an extra mile so that more people can come for this screening.<br>**Interviewer:** Please explain the screening services that are offered in the district.<br>**Participant:** We screen all mothers who come for cervical cancer screening here at the hospital and we also do outreaches to reach those mothers who cannot afford to come into town for screening because there are a lot of mothers out there who are who are saying they don't have money. Like even when we screen them out there, others get positive results and they are supposed to be done LEEP for treatment of precancerous lesions at the hospital, but they are saying they don't have money to come to Gwanda and for treatment.<br>**Interviewer:** When you do outreach services which areas do you cover and how often do you go to those areas?<br>**Participant:** All Rural Health Centres and we visit them every Saturday. We cover a different RHC each week, that means with the number of RHCs in the district we can visit each center maybe once every six months *if transport is available*.

Interviewer: How is the uptake of screening, how keen are the women on screening?
Participant: Like I said, there are some who are keen, but going down to the rural areas, there are some who are still not aware and so they do not want to be screened, they don't want to come for screening.
Interviewer: How close or far are you at reaching your screening targets?
Participant: Mhh…. I am not sure if we have set targets
Interviewer: Please comment further on the uptake of screening, you said earlier that more still needs to be done, would you like to elaborate further on that?
Participant: Women are not coming but there are some programmes that we are doing which are being sponsored by partners like OPHID. OPHID they say they need to screen women who are HIV positive that's what I think makes clients have that stigma so that they won't come for screening because if you go, it will be known that you are HIV positive. And then there are some clients who are HIV negative, those clients are coming in their numbers, but those who are HIV positive most of them don't want to come. When we are on outreach, we will be in need of those HIV positive clients, but they are not coming, that's the problem but those who are HIV negative usually they come even if they have not been called.
Interviewer: How then do you deal with the HIV negative mothers who want to be screened when you go on outreach with the implementing partners?
Participant: We still screen them, but they will be few because the nurses in the RHCs would have been told to mobilise those who are HIV positive only. So that means those who are negative are not given the opportunity to be screened, so that's the challenge we have.
Interviewer: What guidelines do you have on the screening processes?
Participant: Our national guidelines are saying all clients who are sexually active should be screened. We are supposed to screen all mothers from the age of 18 years and above but there are some young people who are having sexual activities at an early age, so we are saying everyone who is sexually active. We repeat for HIV negative clients after 3 years and for HIV positive clients we are screening them every year.
Interviewer: You mentioned that you do outreach clinics every week, what other strategies do you use to try and reach every eligible woman and motivate them for screening?
Participant: No, we do not do anything else apart from the outreach
Interviewer: In your own view, why are most women not attending the free screening services in the district?
Participant: I think they may not have the knowledge but there are some who are saying it's scary that's why they do not want to be screened. I think they need more sensitisation.
Interviewer: Could there be any factors from the side of health service delivery that contribute to women not being adequately covered with screening?
Participant: I think more staff should be trained and I think the service needs to be decentralised so that the clinic nurses cater for those women who do not have money to come for screening and those women who are unable to be screened during outreach programmes, they can be screened locally.
Interviewer: Could there be any issues to do with religious and other beliefs?
Participant: We have those from Madida church who are not coming to hospital but otherwise for the majority of women, they just don't want to come, they are saying it's scary.
Interviewer: Could there be any factors within the health system which could be used to attract more women into screening without pouring in a lot of resources?
Participant: Only sensitisation, especially to those who are in rural areas, some of them really don't have the knowledge. They don't even know what this is all about because if you teach them that's when you will find out. These people they don't have knowledge about the screening programme, so I think we need to go out there and sensitise them
Interviewer: What would you like to see changed in the programme to make it more accessible to more women?

Participant: More outreaches should be done because the outreach which we are doing now as I said is being sponsored by OPHID and they only screen HIV positive clients and those who are HIV negative are not screened. Women don't have money to come to Gwanda for screening so I think more outreach programmes would work for the rural areas to include HIV negative women so that more women would be screened. We usually go on a Saturday and that's once a week and only screening HIV positive, so those who are HIV negative are left out.
Interviewer: For those women who come to your VIAC clinic for screening, do you screen a specific number per day such that if more than that number come the others may not be screened on that day?
Participant: We do not have a target for the day. We never send any women back. We are able to screen everyone who comes because they don't come in big numbers that's why we are able to do all of them.
Interviewer: Is there anything else related to challenges women face in accessing screening which we haven't discussed?
Participant: Screening should be delivered as a full package. Every woman who visits the hospital should be referred for VIAC screening because some of them don't even know about this screening. All women coming for health services to any hospital department should be asked if they were ever screened and if not, they should be referred for VIAC. Usually, we screen these women who come direct to the VIAC clinic, so I think a lot has to be done by all hospital departments especially the OIC. There are clients who come to collect their medications, they are also supposed to be referred. FHS (Family Health Services) should also refer their clients so that all women get an opportunity to be screened.
Interviewer: In your opinion do you think nurses working in other departments are well informed to deliver appropriate information on cervical cancer and screening?
Participant: Mmhh, I am not sure. They are not trained but they do have pamphlets and charts which they can refer to. It would be better to have VIAC trained nurses in all departments
Interviewer: Is there anything else you would like to add?
Participant: No, I think we have covered everything.
Interviewer: Thank you for your time and for all the information which you have provided. The information will be useful for the study. A summary of the findings will be presented to the Hospital authorities at the end of the study.

IDI 7

Interviewer: Thank you for your participation in this study. As already explained, we want to assess the performance of the VIAC programme so that we identify areas which need to be improved in order for more women to access the screening services. Could you kindly tell me how you are involved in the cervical cancer programme?
Participant: Ok, I trained to be a VIAC nurse long back in 2015. When I trained I was under the Ministry of Health by then. So, when I finished my training, it was training for two weeks, actually we were trained for two weeks whereby we were taught on screening of clients for cervical cancer. We were also trained on how to treat those clients found to have precancerous lesions. We were also trained to identify any…. any…. any…. problems on the cervix so that we can treat. Especially you can find a client with STIs. We were also taught on how to interpret the results and treat. Then I started working in the VIAC department since 2016 at a district hospital whereby I was the first person to be selected at the hospital. Maybe they discovered I was competent I am not sure, but I was the first nurse at that hospital to be trained and a doctor. I screened for about 2 years at the hospital. First days it was very difficult because when the programme started, we didn't do like what those who were doing circumcision did. We didn't do those mass campaigns. We were just identifying clients from outpatients and the wards and take them for VIAC. That's how we started so we had a lot of challenges because most of the clients which we were screening were VIAC positive because those were the ones coming to hospital seeking treatment. It was just a tip of an

iceberg because we didn't have the picture of what was happening. Then I was there at that hospital for two years and I left the Ministry in 2018 November to join an NGO OPHID. At OPHID actually when we got the contracts, we wanted to….to help assist in identifying clients, more clients in fact because OPHID deals with figures. So, they wanted more clients to be screened. So, they wanted someone who is competent who can easily identify people in the community. So, I joined OPHID in November 2018 and my role in OPHID was to do cervical cancer screening in high volume sites. So, the high-volume sites which were selected in Matabeleland South were Gwanda, Beit Bridge and Plumtree. So, I was deployed here in Gwanda in 2018.
Interviewer: OK.
Participant: First days it was very difficult because I don't know, maybe people don't take VIAC seriously, maybe it's the attitudes or I don't know. So, we were having a lot of challenges. I still remember my first day here in Gwanda, I only screened 7 clients, only 7 the whole day. Out of those 7 the target group which I was supposed to screen were only 2. Then I started to talk to the sister in charge because I was working under the sister in charge. Are there any challenges we have at the hospital? Is it us the staff or is it the client's attitudes, do people have the knowledge? Then we discussed with the sister in charge and I said ok every day first thing in the morning sister kindly allow me to go do health education first at OI, OPD and FSH because I was targeting entry points. Sometimes I would go into the wards and ask; sister can I discuss VIAC with the patients so that they know. When they are stable, they can come for screening. That's when I become familiar with the community and screening figures started to increase. So, I discovered that people didn't have enough information on VIAC and CC.
Interviewer: Ok. So, according to your assessment, how prevalent is cervical cancer in the district?
Participant: Ummmh, it is high, very high. In this community I have discovered there are a lot of miners. I think the lifestyle in this community also contributes to this high prevalence.
Interviewer: You mentioned that initially you were seeing very few clients and you realised that the knowledge levels might have been low. So, what's your assessment now? Does the community have better knowledge and has there been an improvement in their awareness that such a programme exists?
Participant: They are aware but not to that level we want because we wish eehh, our wish is to do those mass sensitisations so that we are sure everyone is now aware. Of course, those who come to the hospital are the ones we target because they come to us, but we are not yet at that level where we reach out to people and we talk about it, give information so that the people can be informed so that they can come. We haven't reached that level yet.
Interviewer: So, what are the women's perceptions on screening?
Participants: Others they say that if you do cervical cancer screening you will end up being stressed with the result, others say it's painful, others say the nurses go around talking about you, this thing of VIAC is about your private parts so they will go around telling people about you, so they will actually say I won't go to the hospital, as long as a certain cadre is there. I won't because she will go around telling people about my condition.
Interviewer: Ok. Do you have screening guidelines which guide you on who should be screened, when to start and how frequent etc?
Participant: Our SOP?
Interviewer: Yes.
Participant: Ya, we use an SOP because if you don't use it, it will be very difficult. Actually, OPHID has its own guidelines but at the same time we also use the Ministry of Health guidelines because as OPHID we are there to give technical support to the Ministry so we follow whatever the Ministry wants while at the same time I will be targeting the figures for OPHID, but also doing what the Ministry wants. I will do both. The Ministry will say I want to screen all women of child bearing age, actually all women, whether you are HIV positive or negative, we follow that. So, when a client comes, and they are HIV negative I screen. When the client is positive I also screen. Thereby by I am

following both the guidelines of the Ministry and those of OPHID. But they interlink it's one and the same thing.
Interviewer: So, what's the focus for OPHID? Which women are you targeting exactly?
Participant: OPHID is targeting HIV positive women and their target group at the moment is 25-49 years. That's their target group. But we screen everyone who comes for VIAC. But at the end of the month OPHID wants the statistics on that target group.
Interviewer: You have mentioned that the strategies which you used for motivating women was going department by department, that was you doing that. So, would that be the strategy still being used at the hospital's VIAC clinic?
Participant: Ummmh, I am not sure if everyone else does that. Because at the moment I am here at Phakama clinic but there are clients from the hospital coming here, sister we were looking for you, we want you to screen us. But I will explain to them there are nurses that side. No sister, they don't explain the way you do. The way you explain to us we really understand I don't know maybe it's the attitude of nurses or she will be over whelmed with work because in the hospital we do VIAC in ANC. So, they do multiple jobs. They can do bookings and when a client for screening comes, they do VIAC, so I am not sure. But at the moment, information dissemination by the hospital's VIAC staff is a challenge.
Interviewer: What could be the other challenges which deter women from screening?
Participant: Ya, I have discovered the main challenge they always tell me is about accessing the services. If I phone them maybe as a follow up after screening, they always say they can't raise money to come, it's far. Can't you come here to us on outreach so that you attend to us here? Then the other challenge is male involvement. Other ladies are afraid to come for screening because they are afraid of their husbands. Let's say the woman comes and screens and is found positive, then we need to involve the husband in the treatment whereby after treatment she is supposed to abstain from sex for 6 weeks. So that's where the challenge is. So, the lady will prefer not to screen because she will say no I will have challenges in my home as he will get it from somewhere. And, the other challenge is the attitude of clients. Ah. That one is too much.
Interviewer: Could you please explain more on the attitudes of clients?
Participant: The information which always circulates in the community is that VIAC is painful. The instruments they use Mmmmh, and they reuse that instrument before they clean. I don't know whether you are getting what that means.
Interviewer: Yes.
Participant: So, when a client comes, we really have to show her the sterile pack that is sealed.
Interviewer: Could there be any other factors which deter women from screening?
Participant: I can't think of anything else.
Interviewer: We have discussed a lot of barriers to screening, so how do you think these barriers can be overcome?
Participant: Those barriers, ya, I think the first thing we can do, let's go to the community before we screen these ladies. I think we need to track the ladies even up to grassroot level. We sensitise them; we educate them so that they have knowledge because knowledge is power. If we manage to do that I tell you; every lady will want to be screened. The other barrier we need to address is those in hard to reach areas. We make outreach programmes to be more frequent. Here in Gwanda, we have got how many clinics, they around 40 I am not sure. We can make sure that every 3 months we go to each clinic, we screen after they have sensitised on when the outreach team is coming to the clinic. That's how we can reach women in those hard to reach areas.
Interviewer: At the moment how often are the outreach clinics done?
Participant: Ah, in fact the outreach clinics are being funded by OPHID, but they are not done on regular basis. At the moment they were not going because of funds. But very very soon we will start outreaches again. But there is only one team which caters for Gwanda South and Gwanda North, of which this is not enough. If we could have 2 teams so that the other will go south and the other will go north. We can screen much more women.

**Interviewer:** Am I right to understand that the outreach clinics are done depending on the availability of funding, and that the frequency is not often, and that they have been put on hold for some time now?<br>**Participant**: Yes, yes. It's not consistent. And, it's only being done by nurses from Gwanda hospital at the moment. The whole of Gwanda district. They do it on a Saturday when the VIAC clinic will be closed.<br>**Interviewer**: Thank you for the very informative discussion. Is there anything else you would want to comment on?<br>**Participant**: I always have imaginations, if possible, I wish we could link with that clinic which is called the Sister's clinic. The Sister's clinic has got so many clients, considering the job they do.<br>**Interviewer**: Kindly explain on the job they do.<br>**Participant:** Those ladies who go to the Sister's clinic are the ladies of the night.<br>**Interviewer**: Oh!<br>**Participant**: Yes. There is a clinic here in Gwanda which caters for the ladies of the night, for all their sexual and reproductive health needs I think so, but I don't know which organisation funds it.<br>**Interviewer:** Ok.<br>**Participant**: Because I always meet ladies saying I went to the Sister's clinic and they said I should do VIAC. But there are many, many, many. I don't know how best we can link with that clinic. It's another barrier, because those ladies at the moment, right now as we are talking those ladies are sleeping. They sleep during the day and start working during the night. If possible, for us to be able to reach them, maybe the staff there can talk to them, then we do VIAC for them from 5-6 after the clinic has closed because they don't want to be seen at the clinic those ladies. Actually, they are the ones carrying HPV because if you ask them about their sexually history, they tell you per night I can meet 15 clients. Are you using protection? Not with all of them because money so, hey, that's why the prevalence is so high in Gwanda.<br>**Interviewer**: Ummmh, Do this Sister's clinic screen for cervical cancer?<br>**Participant:** No, they don't, they don't, so that's why I 'm saying there should be a way to link with that clinic.<br>**Interviewer**: Thank you again for the fruitful discussion and please continue with the good work.<br>**Participant**: Thank you. Actually, next week I will be working from the hospital. We will be doing a LEEP campaign on all the women who tested positive on VIAC and have not been treated due to COVID-19. We have a backlog. There is a big list I had compiled, and we need to clear them. Thank you.<br>**Interviewer**: Thank you again. Good Afternoon.<br>**Participant**: You are welcome.
**IDI 8**
**Interviewer:** Good afternoon and thank you for your participation in this study whose findings I hope will contribute towards development of strategies to improve access of cervical cancer screening by women in the district.<br>**Participant:** It's alright.<br>**Interviewer:** So how involved are you in the cervical cancer screening programme?<br>**Participant:** I as a nurse at FSH department I attend to mothers those who come for post-natal visits and we encourage them to go via VIAC for screening, especially those who are HIV positive and those who have not done any screening before.<br>**Interviewer:** Ok.<br>**Participant:** We make use of the schedules like when someone has been screened like maybe HIV positive isn't it they are saying after Mmmmh (seems to be thinking) after a certain period you have to go for another screening and if you are HIV negative you go after 4 years so; we have to follow those schedules.<br>**Interviewer:** So how long have you been working in this department?<br>**Participant:** Since January 2020<br>**Interviewer:** Have you received any training in VIAC?

Participant: No
Interviewer: So, in your opinion, how prevalent or how common is cervical cancer in the district?
Participant: Mmmmh. Maybe the question is not clear to me.
Interviewer: How frequently diagnosed is cervical cancer among women in the district? Do you have a lot of women suffering from cervical cancer?
Participant: Ok. I cannot say of a percentage or number but when I was working in the female ward in 2016, there were a lot of women who were VIAC positive and some were with cervical cancer who had been staged 3 and 4. I have seen a lot. But then there was this Dr Gunguwo who was coming from Bulawayo to do the TAH (Total Abdominal Hysterectomy), TVH (Total Vaginal Hysterectomy). So, I can't say it was for Gwanda district only because some of the women were coming from other districts even as far as Bulawayo because Gwanda was the one offering these services at a cheaper price for those procedures the TAH or the TVH.
Interviewer: I see. In your opinion, how would you rate the knowledge of the community on cervical cancer and what are their perceptions on cervical cancer.
Participant: What I'm seeing is that women are now knowledgeable, but I cannot say they are coming because VIAC is not done in my department. So, I can't say 100 %, but people are coming for screening, so they must be having the knowledge.
Interviewer: Ok. How are cervical cancer screening services delivered in the district?
Participant: Cervical cancer screening is done at antenatal department and I think out-patients department, but they are the same people who are doing that screening. And, apart from out-patients department and antenatal clinic, now they have another room at Phakama clinic where it is done. I'm not really sure how often because they are the very same people who are doing it at Gwanda provincial hospital and are also supposed to go to Phakama clinic to do those screenings. So, maybe they have scheduled days to say on such and such a day is VIAC or something like that.
Interviewer: You have already expressed that women know about this programme…..
Participant: Interrupts, yes.
Interviewer: So, when they come to you here, what education strategies do you apply?
Participant: At this department, the FSH department, mostly the women we attend are people who are having babies at a small age 25 years and below. So, when we ask those women have you been screened, they will be saying no. Maybe it's just because of age. But I think those women who visit the VIAC clinic are 35 years and above, the ones who know the impact of being screened and not being screened. Because here at FSH we don't normally see those 40 years and above, it's rare, but those are the ones who are going for screening mostly.
Interviewer: What guidelines do you have on the VIAC screening programme for you to know the age group which should be screened. You have just said those 35 years and above are the ones who go for screening?
Participant: What I know is that every woman should go for VIAC screening and the schedule is that those who are HIV positive have a time frame to come for repeat, and those who are HIV negative have a time frame.
Interviewer: You mentioned that in your department you see young mothers who may not know the impact of not screening compared to the older mothers who go for screening. So, are there strategies in place to reach all women who are eligible for screening with information so that they are motivated to go for screening?
Participant: I think the strategies are just health education in every department. Like in female ward there are some women who come maybe without uterine problems. They can come with diabetes and they can come with high blood pressure. So, I think on admission heath education should be imparted to every woman including VIAC, HIV testing, all those things. At the out-patients department same thing should be done. Health education should be imparted to every woman, even to partners, so that they give the information to others at home. Every department that is maternity, female ward, out-patients, where ever in entry points. Health education should be given to everyone so that information can spread. We can't say only where the screening is done because you will be

giving information to someone already with information. What about the one who is visiting the out-patients for re-supply of treatment. They will not access VIAC if health education on screening is not given.
Interviewer: You have said a lot about what should be done in all departments in terms of health education on VIAC. What is the situation on the ground though? Is what you have said what is really happening?
Participant: That should be the ideal because for example in female ward the women who were coming already had results which means they got the education from somewhere, maybe UBH because that's where most screening was done. I am not sure though if the education is given.
Interviewer: So, in your own view, opinion or assessment, why do you think most women in the district are not utilising these screening services?
Participant: I think it's all about may be less nurses are trained in VIAC. May be its only in the last 2 or 3 weeks that some cadres were trained. Otherwise, all along if it was not 2 nurses from Gwanda provincial hospital and one from an NGO only. Those were the ones who were doing the VIAC screening at this hospital. So, you see these things, one can go on leave, one can be off sick, all those things. So' it's not to full capacity on daily basis that screening will be done. And it is done in the ante natal clinic where that same nurse is supposed to do the antenatal clinic so, it won't work well. Maybe I can say training of more cadres.
Interviewer: Please elaborate how staff shortages affects women accessing the service.
Participant: Maybe it can reduce the number of patients screened on that particular day. Because you can't say on daily basis those 3 nurses will be there every day.
Interviewer: Could there be other contributory factors apart from the staff shortages?
Participant: I can say now all that I am now talking is admin related; infrastructure. We should be having a specific area to say VIAC. We can't leave it in ANC because some old women will say I am not of child bearing age and I cannot be seen going to ANC. People will see me that side and think that maybe I am pregnant. Seeing an 80-year-old woman at ANC? It doesn't make sense. But when there is a VIAC department you know every woman should go that side.
Interviewer: We have discussed on the health system challenges which could be affecting the uptake of screening. Could there be any other factors outside the health system which could pose as barriers to mothers accessing screening?
Participant: As much as I know in Gwanda, many churches will say do not go to hospitals. Especially these white garment churches. So maybe these can be other issues that make women not to come.
Interviewer: You discussed about the shortage of VIAC trained nurses and recommended that more nurses should be trained.
Participant: Yes, trained. Or if I can say every department, maybe the female ward, maternity and FSH there are cadres who are doing VIAC so that it can be done anytime if a patient says I want to be screened, there and then that patient can be screened. Like someone who has come at FSH for 6 weeks, then you say go for VIAC, she will go there and line again. Here she was facing another line, then in ANC another, maybe she goes on a wrong line for ANC mothers. You don't even know where exactly you are supposed to queue. These are other factors. The person will end up saying I will come another day and those are missed opportunities for someone to be screened.
Interviewer: If you were in charge and had the powers to make some changes which would make the programme more accessible to women and service delivery much improved, what would you do?
Participant: The first thing would be to train more cadres for VIAC screening. As many as possible. Just like in HIV testing. In any department there is a nurse who can test HIV. Anyone who is in any department can request a test and be tested there without being told go to outpatients that's where it's being done.
Interviewer: Anything else?
Participant: Infrastructure to have a proper VIAC unit. And it should be nearer to OI because those are the client's we need to keep an eye on.

Interviewer: Ok.
Participant: Yes.
Interviewer: Is there anything else you would like to comment on which we have not discussed?
Participant: I think another way of catching other women is to check with them in all the departments if they have been screened or re-screened. Then they are asked to visit VIAC for screening. Just like what they are doing for viral load. If you are due, you are sent for that before the re-supply. I think it's another way of making sure that most women will be screened. Although it will also be a challenge if many women come to VIAC because there will be no adequate nurses to screen, no packs, no benches, so infrastructure as well.
Interviewer: How could those in rural area be assisted?
Participant: I just overhear that those nurses doing VIAC at the ANC do some outreaches. I am not sure how often, but I once heard that they are doing some outreach. I don't know how effective is it but that is a step in the right direction. They should continue with more of those
Interviewer: Thank you so much for your thoughts which will add value to this study. I will share the findings with the hospital authorities at end of the study.
Participant: Thank you.

IDI 9

Interviewer: Good afternoon and thank you for taking your time for this interview. I would like to hear your views about the screening services in the district with the goal of improving the service delivery so that more women can have access to screening. May I kindly hear from you how you are involved in the cervical cancer programme.
Participant: Keeps quiet for a long time.
Interviewer: Some service providers play a clinical, administrative or educative role in cervical cancer screening and I would like to find out the extent to which you are involved.
Participant: None of the above. Usually here we don't deal with VIAC patients. We refer them to the ANC clinic. If a patient comes, we just screen and refer them to the VIAC clinic, that's where everything is done.
Interviewer: I understand that, but there are many components to cervical cancer screening. When you first attend to the client, for you to refer her to the VIAC clinic, you would have done something for you to decide to refer them there, and that's what I wish to hear from you. What role do you play as far as the programme is concerned.?
Participant: It's just referring to the antenatal clinic. Even the health education is done there.
Interviewer: The way I get you is that in the out-patients department you don't give any health education on VIAC at all.
Participant: Yes.
Interviewer: Ok, so when you refer them, what would have prompted you to refer them there?
Participant: I just take a history, then refer them to ANC.
Interviewer: So, what kind of history would they have presented with for you to refer them to the VIAC clinic?
Participant: Vaginal bleeding and sometimes vaginal discharge.
Interviewer: What discussions do you have with them before referring them to the VIAC clinic.
Participant: We do not discuss anything; everything is done at the VIAC clinic as I have already said.
Interviewer: Ok. How common is cervical cancer from the cases you see in the department, or even its prevalence in the district.
Participant: Laughs, I don't have an idea.
Interviewer: In your own opinion, how do you rate the knowledge of women on cervical cancer and cervical cancer screening?
Participant: Some come and request to be screened, so I suppose they know.
Interviewer: So, in your view, they have knowledge because they already come with the intention to be screened, how would you assess the uptake of screening in the community?

Participant: Sometimes we initiate them to be screened but I have no idea of the uptake of screening.
Interviewer: To me, if you initiate them you would have played a role, and that role you play is what I would love to know.
Participant: We initiate them.
Interviewer: Are there any guidelines or standard operating procedures on cervical cancer screening that you are aware of?
Participant: No
Interviewer: Apart from those who come asking to be screened, are there any other strategies which the department uses to try and create awareness on screening among women.
Participant: No
Interviewer: In your opinion, why do you think women are not fully utilising the screening programme which is available for free? What could be their reasons?
Participant: I don't work in the VIAC department and I don't have the statistics which would make me say women are not coming forward to be screened.
Interviewer: The statistics are already available. The national screening target was 50% screening coverage by end of 2020 and Gwanda district is still below that. May you kindly suggest reasons for this low uptake?
Participant: People are not aware of the programme.
Interviewer: Ok. Looking at the influencing factors from a holistic approach, what could be the other reasons?
Participant: Sorry
Interviewer: We want to look at the factors from the perspective of the individual, the community and even from the side of the health system.
Participant: There should be education of patients at every entry point so that they have knowledge about screening.
Interviewer: Could there be other factors apart from knowledge?
Participant: I don't know.
Interviewer: Ok. You mentioned that women need to be educated to create awareness on cervical cancer screening, what else could be done, what would you recommend which would encourage these women to come for screening?
Participant: Offer pamphlets and conduct outreach services.
Interviewer: Currently, would there be outreach screening services being conducted?
Participant: Not sure if they are still doing them, they used to.
Interviewer: As out-patients department do you have the national cervical cancer prevention and control strategic document to guide you in the delivery of cervical cancer prevention services.
Participant: No.
Interviewer: Is there anything else which you would like to comment on which we did not discuss?
Participant: I think you should go to the VIAC clinic that's where you will get the information you want.
Interviewer: Thank you for that suggestion, I have already talked to them. My focus is on these other women who do not go to the VIAC but who you see in other departments like yours. I am hoping to get answers on why these women are not going to the VIAC clinic for screening.
Participant: I have nothing to add. I don't know much about VIAC.
Interviewer: Thank you for your time and your contributions.
****Participant was not very forth coming and seemed not to have much information on the screening programme*****
Interviewer's assessment: Nurse lacks adequate knowledge on CC and screening and lacks motivation

IDI 10

Interviewer: Thank you for allowing me time to talk to you during your lunch hour. I wish to get your opinion on the cervical cancer screening programme in the district. Your answers could contribute to improved delivery of the programme. I would like you to tell me your experiences and role on the delivery of cervical cancer screening services at Gwanda Provincial Hospital.
Participant: Eh Gwanda Provincial Hospital they are doing their best in terms of cervical cancer screening, specifically, our department OIC (Opportunistic Infections Clinic). We make sure that every woman has to be screened. As you see, these are our books you can check them. We indicate that this woman is not screened or is screened. So, we make sure that every woman when they come for collection of their medication, we advise them to be screened for cervical cancer. That's how I am involved specifically those who are on ART.
Interviewer: Ok. Why do you specifically want those on ART to be screened?
Participant: Eehh, because of their … because they have a higher risk. Plus, those are the people we are dealing with as a department. When it happens that the client is not on ART, like those who send their relatives to come and collect the medication for them, we also advise them to be screened for cervical cancer.
Interviewer: I see. How long have you been working in this department and have you personally received any training on VIAC?
Participant: I have been working here for about 3 years but no, I have not been trained.
Interviewer: In your own view, how prevalent is cervical cancer in the district?
Participant: The cervical cancer cases are there, especially if all people are to be able to be screened. Cases are there. But the thing is that people have to be screened for us to identify those cases. The cases are there but few people have been screened.
Interviewer: How do you rate the community's knowledge on cervical cancer?
Participant: An awareness should be done. People are not aware. You will be shocked when they come here, and we tell them about cervical cancer and sensitise them for screening. It's a shock to them. They don't know it. But some are aware because of the media, the televisions, the radios, the newspapers. It's a thing which is being preached everywhere. But some are not aware. An awareness should be done especially in the rural communities. There is need to send people to give them knowledge about it.
Interviewer: Ok. Do you have the guidelines on cervical cancer prevention and control? Like when the patients come to you, how do you decide whom should be screened, when they should start screening, how often, what age should they be screened etc?
Participant: We do not have national guidelines. They are at the ANC clinic where the screening is being done. But here, in terms of age groups, eh, those who are indulging in sex, they have to be screened even if they are adolescents, as long as they are now involved in sex. And every woman who is an ART must be screened.
Interviewer: As a department ok, not talking of the VIAC clinic, do you have strategies which you use to create awareness and motivate women for screening? You have already mentioned that those women who come to you have to be screened including those relatives who are sent to collect the supplies on their behalf. Are there other strategies you use to reach other eligible women for screening who might not necessarily visit your department?
Participant: We focus on those who come here. We do not miss an opportunity to encourage them to be screened, and we tell them to even go and tell their relatives the advantages of cervical cancer screening.
Interviewer: Although the rate of screening is improving, we are still far below the target. In your opinion, what do you think could be the reasons why some women are not coming to be screened yet it's a free service?
Participant: Eh, there is that fear of the unknown, it takes time to convince someone to go for cervical cancer screening. They have that fear; if I go to that thing, what next? Because where can I

get the money for medication. Where can I get the money for other treatments. That's the issue, people have that fear.
Interviewer: I hear you.
Participant: And the other thing people are not aware that it is good for them. People are not aware that it is for their own advantage.
Interviewer: Could there be other reasons?
Participant: The other thing, it might be our set up, yes, our set up. We are saying these patients if they come here, they are going to be screened there at ANC, when the client comes here, they have to travel from here to ANC some, they are not comfortable. They just want it to be done here. At least if they can extend it to close by so that soon after they collect their medication, they can be screened, close by. I think it could be best for them the way I see things.
Interviewer: Ok. So, the way you see things, this department should also provide the screening service?
Participant: That would be the best, that would be the best if resources are there that would be the best. The problem is lack of resources to make this programme effective.
Interviewer: Looking at you community, do you think there could be some community factors which hinder women from screening?
Participant: Other people are influencing each other not to be screened due to fear. That fear thing is everywhere. These are crowd pullers who when they talk others just follow.
Interviewer: Ok. So, you mentioned fear, you mentioned lack of knowledge that some of them do not know. Is there anything else?
Participant: Yes, and the other thing is the financial problems. Some people they don't have money to come here. Usually they even send people to collect their medicines like if the drivers are coming here, they ask them to bring their medication for them. Without that, they don't have money to come. They always say money is a problem. Especially during the lockdown. Most people were not working.
Interviewer: Ok. Thank you. Given all those challenges that you have mentioned what do think can be done for those barriers to be overcome?
Participant: In my own view; one, we are saying our structure, we have to have a room somewhere close by so that those people they cannot walk up there. Some people they come here sick, and that time when they come, we can just grab the opportunity to screen them than to push a wheelchair up there to the ANC clinic, it's a little bit tricky for them. So, if we have our structure here, it will assist. Two, those people who are doing the VIAC thing, I think they must arrange some time to go into the communities and just do it there in the communities for the sake of those people who are not able to come because of financial constraints, it is difficult. So, I think it's best for them to go to the communities every month and do it there in the communities.
Participant: The other thing is awareness. This should be strengthened through the media, the radio, the television so that we reach more people with the advantages to say why are we doing it. We are not doing it for ourselves, but we are doing it for the better of us all at the end of the day. So, some people they are not aware, they are not aware.
Interviewer: Ok
Participant: Sometimes the culture barriers. Some men don't like it to be done on their women. They don't like it. You know the thing which they use for cervical cancer screening. Some men they don't like it. The woman will tell you that my husband doesn't want. They are even jealous of the tools that are being used there.
Interviewer: Very interesting. Is there anything else you would like to add?
Participant: The barriers are the money thing, our structure and awareness. If people are aware, I don't think there would be many barriers because if people know the advantages, they will strive for it. They will even save for it, to come and be assisted.
Interviewer: Thank you very much and sorry for taking your lunch time.
Participant: Always welcome always welcome.

IDI 11

Interviewer: Thank you for participating in this study the findings of which aim at improving the delivery of cervical cancer screening services in the district. We are starting the interview now. Could you kindly tell me how you are involved in the cervical cancer screening programme?

Participant: I'm not actually hands on. I work here in female ward. So sometimes we see patients who are admitted from casualty being referred for VIAC screening and then we take them to ANC for VIAC to be done. Some they come VIAC positive, then they are booked for total abdominal hysterectomy. So, we are involved in preparing them for theatre to have the hysterectomy. That's how I am involved.

Interviewer: Ok, have you received any training on VIAC?

Participant: No

Interviewer: How would you rate the prevalence of cervical cancer in the district based on your work experience?

Participant: Mmhh, I started working here in January 2020, then in March we were interrupted by COVID-19 and I went to maternity. Then in September I come back. So, it's about 8 months in this ward. Having been here, I think I have seen about 4 women who have been diagnosed as CA cervix. Some are for palliative care. I think I have seen about 10 women coming for total abdominal hysterectomy because of cervical cancer. I have seen many who are VIAC positive.

Interviewer: Ok. So, how would you assess the women's knowledge on cervical cancer? How knowledgeable are they about the disease and screening?

Participant: Really, I think a lot of women have no information about the disease and cervical cancer screening. Why I'm saying so is because most of the women who come here, they are in the late cervical cancer stage where they are now booked for palliative care. Not sure really about the rural areas but women I have seen here, when you talk to them about cervical cancer and even the screening, you realise their knowledge is very little.

Interviewer: What cervical cancer screening services are offered in the district?

Participant: I'm not sure but I heard that the VIAC nurses now go out to do outreach programmes to screen. Whether they do it monthly or weekly, I'm not sure. But I have seen them carrying their kits going out to the rural areas. And now they also go to Phakama clinic where they also do the VIAC there. I'm sure they did it because some cannot afford to come to this side so they can go to Phakama. I'm not sure how it was arranged. But when they are going out, I think they go with NAC (National Aids Council). They have a programme with NAC I think when they go out to the rural areas.

Interviewer: That's good progress. You are saying it's the staff from the hospital which goes to Phakama clinic?

Participant: Yes, there are few trained nurses. The same nurses who provide screening here go there to provide screening services at Phakama, and the same staff again are doing outreach services with NAC.

Interviewer: How keen or unkeen are the women on screening going by your observations?

Participant: Mmhh, here in the mornings, we usually do talks at ward level where we tell them about HIV screening, we also tell them about VIAC, and we also talk about family planning services available from FHS department. Even on one on one, we tell them about VIAC screening.

Interviewer: Ok.

Participant: Yes, some are interested and have been screened, but some still believe in the traditional ways of treating. They will tell you I have an aunt who was diagnosed with CA cervix and she was helped by the traditional healers. Most have never been screened for cervical cancer, and for some it's a new thing they will say, oh, I didn't know, and it's done for free. Then they go. So, it differs with individuals.

Interviewer: I see. Are there any guidelines from the national or institutional level for VIAC screening?

Participant: I am not aware of any guidelines. May be at the VIAC clinic they have them. I am not aware of any.
Interviewer: Ok.
Participant: What I know is that when I screened, I was told that if you are HIV negative, you are screened after 2 years and if you are HIV positive, you are screened yearly. That's what I heard ya.
Interviewer: You mentioned that you give education talks including cervical cancer screening at ward level which is very commendable. Are there any other strategies you use to reach more women with cervical cancer screening knowledge in addition to that?
Participant: Ah no
Interviewer: Ok. In your opinion, why are women are not adequately utilising the free cervical cancer screening services?
Participant: I think mostly its knowledge deficit, I think knowledge. Really sensitisation should be stronger at rural level. I think many of them would really love to be screened if they knew. But they only get to know about it when their neighbour has been diagnosed with cancer and that's when they are aware that oh, something like this is there. So, I think if they could get the sensitisation in the rural areas, deep down there they would really come forward.
Interviewer: Mmmmh
Participant: Ladies are not like men who shun hospitals, ladies are willing to be assisted whenever they are aware of any health programme.
Interviewer: Ok. Could there be any other barriers to screening apart from what you have already mentioned?
Participant: I think even if some women want to be screened, the fact that VIAC is only done at Gwanda provincial hospital is a challenge. Funds for travelling. Even if someone has heard about the VIAC, money then to come to Gwanda poses a barrier to them. Some are diagnosed here, and they are referred to the central hospitals for treatment, but they don't have the funds to travel to Bulawayo. Funds are a problem.
Interviewer: Could there be any other issues contributing to women failing to be screened, maybe related to culture?
Participant: Definitely, socio-cultural influences are there. Most women are socialised into the traditional ways of dealing with their reproductive health illnesses. They will tell you my grandmother knows how to insert herbs and whatever to cure the problem. And they will tell you they are talking from experience. My grandmother is able to treat this. So, they have it in their heads that the traditional treatment is best. That's how some of them are.
Interviewer: Ok. So, in view of these factors which may be hindrance to screening, what recommendations would you give to address these problems so that more women would be able to access screening services?
Participant: I think on the issue of funds, the programme lacks funding. If VIAC could be done at every clinic, many women would be screened. At every clinic women should be able to have the VIAC test done. Then, regards socio-cultural beliefs, it's difficult to change someone's beliefs. I don't know really how we can intervene on this one. But, maybe by including the elders there to be the ones who address them and tell them of the cervical cancer screening programme. To include elders of that community because mostly in rural areas people listen to their elders much better than any other person. Maybe they should be involved as part of the stakeholders in cervical cancer screening, then they take the word back and sell it to their communities. *And also, that every nurse or department be well versed with VIAC screening because it must not be taken for granted that nurses know because that is not always the case.* Especially in FHS, you find that I'm trained in IUCD insertion, but I have no knowledge in VIAC. Maybe when I'm doing a procedure on IUCD insertion, I can see something that this cervix is not ok. If I had that knowledge, I would refer her earlier to the VIAC nurses. But now it would be guess work. So, if every nurse could be trained in VIAC. Funding should be availed to have every nurse trained in VIAC screening. I think it would also help.

**Interviewer:** You also mentioned the knowledge deficit, and suggested that awareness should be created?<br>**Participant:** Yes, and sensitisation.<br>**Interviewer:** Yes, now, which would be the best way to do it to reach out to more of these women?<br>**Participant:** Unfortunately, when it comes to reading, people do not read. Even if you make pamphlets and scatter them everywhere, people are reluctant to read. Maybe if we could have those what do you call them, there road shows, yaa maybe we could have road shows to attract the attention of people where they will talk about it. I think it will be better because reading aah most people don't read. Even if you print those in Ndebele, Shona and English aah, very few will read, yes one will read but many won't read.<br>**Participant:** Maybe also to make use of radios and the media will make people pay attention. This will help them see that there is something special going on.<br>**Interviewer**: Ok. Could there be anything else you would want to comment on?<br>**Participant:** I think I have said all I can think of.<br>**Interviewer:** Thank you so much for your participation, it has been very fruitful discussion.<br>**Participant**: Thank you to you too.
**IDI 12**
**Interviewer:** Good afternoon and thank you for agreeing to participate in this interview.<br>**Participant**: You are welcome.<br>**Interviewer**: Could you please explain to me how you are involved in the cervical cancer programme?<br>**Participant**: Laughs and whispers "I am not directly involved".<br>**Interviewer**: You are free to tell me anything about the cervical cancer screening programme in this hospital or what you do in your ward to link women with the VIAC clinic.<br>**Participant**: Ok. Seeing as I am working in the Paediatric ward, we deal with mothers. So mostly we encourage them to go for cervical screening, but we do not deal with that in detail. We only tell them to go to the VIAC clinic. That is where everything is done, that is where everything happens. *We don't explain much in the Paeds ward because we do not have much information.*<br>**Interviewer**: How long have you been qualified, and have you received any training on VIAC?<br>**Participant**: I qualified 2 years ago and no, I have not been trained in VIAC that is why I do not have much information related to VIAC screening.<br>**Interviewer**: According to your own assessment, how prevalent is cervical cancer in this district? How often do you come across patients with confirmed cervical cancer?<br>**Participant**: I think it's very common. We see a lot of cases in the female ward. Women have to undergo hysterectomy. I think there's a programme once a month where a lot of women come to have hysterectomy done due to cervical cancer. I am not sure now with the COVID situation.<br>**Interviewer**: How would you rate the knowledge that the community has on cervical cancer?<br>**Participant**: I think people don't have much knowledge.<br>**Interviewer**: Would like to elaborate on that?<br>**Participant**: Ok, what happens is; especially the patients that I have seen when they come here, the cancer would have moved to another stage, and they only come here at a later stage. If they had knowledge on cervical cancer, they would be coming earlier when they start experiencing symptoms that could be in line with cervical cancer.<br>**Interviewer**: What do you think their perceptions are on cervical cancer, what are their thoughts on screening, their beliefs, their opinions?<br>**Participant**: I think maybe due to not being educated enough, they don't think screening is important that's why many don't bother about screening, they only take it as a priority when it's now at a later stage and they experience symptoms. When they are now being told its cancer that's when they start taking things seriously. But then they tend to relax and think that cervical cancer happens to some other women and not all women, and they only take action when they have been diagnosed or when they now have symptoms, or they are now in pain.

Interviewer: Ok. What guidelines do you have on the VIAC screening processes?
Participant: No, not here. I am not aware of any guidelines. Maybe at the VIAC clinic
Interviewer: You said you refer mothers from this ward for screening. So, what strategies do you use, whom do you refer, do you have scheduled activities which you use to create that awareness on screening, or you educate women when you get the chance?
Participant: I could say its opportunistic. When we are just talking to them and getting to know them and asking maybe about their child that's when we start maybe introducing that part. We advise them to go for screening. But I cannot say there is a certain way we do it, we just talk to them regularly.
Interviewer: Ok. What factors do you think hinder women from accessing screening?
Participant: It could be distance, it could be distance because this is a referral hospital, in as much as we have surrounding areas like Phakama but most of the people come from the rural areas and I think it's hard for them to come. Already there is no transport and there are also financial constraints. And also, the fact of not being educated enough, they don't see the need to come for screening, only unless and until it has progressed.
Interviewer: You have mentioned distance and you have mentioned women's lack of knowledge, could there be any other hindrances? May be even related to health services and community factors which pose challenges for women related to screening?
Participant: People have different beliefs; it could be religion. Some don't believe in going to clinics but believe God can treat all illnesses and if you go to hospital, it's a lack of trust in God. That's what I can think of.
Interviewer: In your opinion, how supportive are spouses and families as far as cervical cancer screening is concerned?
Participant: Most of the patients that I have seen, they come to the hospital alone, so I would not say they do not have enough support. They actually don't have enough support, because even when they come, I have never seen anyone come with their partner. They have to go through with this all alone.
Interviewer: So, how do you think these barriers could be overcome?
Participant: Eehh, I think eehh, the issue of education comes in whereby they need to be educated, the partners need to be educated too. And since we all know that it's difficult for men to come to a clinic setup, maybe eehh…. the people responsible for the screening programme can go out there to communities and invite people including their partners then they can be educated. I think that way we can actually sensitise a lot of people and that way we can also talk to a lot of partners rather than asking them to come to the hospital so that we educate them. I think we have to go to the community.
Interviewer: Ok. So, when you say the people responsible for screening, who exactly are you thinking of?
Participant: The team responsible for outreach. The OPHID guys.
Interviewer: If you were in charge of the VIAC programme what changes would you make to ensure that the programme is more accessible to women?
Participant: I think mobile clinics, regular mobile clinics could be put in place whereby the team would go to the community to do the screening there, then if there is need for the patient to come for any further investigations, I think transport should also be provided for them. That way we can cater for a lot of women. Because I think it's pointless for you to go out to the community and sensitise them and then ask them to come to the hospitals. Because of financial constraints and distance, they might not be able to make it here.
Interviewer: Could there be anything else?
Participant: I think I have given all I can at my level.
Interviewer: Thank you so much for the insightful discussion.
Participant: You are welcome

**1D1 13**
**Interviewer:** Good afternoon again and may we now start?<br>**Participant**: Yes, good afternoon to you too.<br>**Interviewer**: Thank you for agreeing to participate in this study. I wish to hear your views on the performance of the VIAC programme in the district with the goal of improving women's access to screening. Hence, I would like to hear how you are involved in the cervical cancer screening programme.<br>**Interviewer:** Aahh, we are not highly involved as such, but we come in by giving health education, the significance, the need for them to undergo that procedure. So, we are almost like sensitisers.<br>**Interviewer:** How long have you been working in the department?<br>**Participant:** I have been in this unit for 3 years.<br>**Interviewer**: Have you received any training in VIAC?<br>**Participant:** No<br>**Interviewer:** So, in your own views and observations and interactions with mothers, how common is cervical cancer in the district?<br>**Participant**: Ummmh, on that I cannot say much, from my department it's hard to say. But I happen to pay visits to other departments like the female ward. That's where you hear staff talking about many patients who have been admitted with cervical cancer. In this department, no.<br>**Interviewer**: In your opinion, how knowledgeable are the women in this community about the disease and the screening programme.<br>**Participant**: From my own observation, I can say probably the women have the knowledge. This is because in the community where I live, the women ask and probe about the programme, where it is done and if there is a doctor who examines people and are we still offering the services. Surely that shows the women now **have partial knowledge** that's why they ask about these services.<br>**Interviewer**: Ok. As you have said that women ask about the services, how keen are they on screening?<br>**Participant:** I cannot say its many who are asking, but it's an average number. On Mondays we used to go to ANC to witness the statistics from VIAC on the number of clients that they would have screened. It's an average number who are screened, **not much.**<br>**Interviewer**: If I'm hearing you right, you have meetings every week, what is discussed in those meetings?<br>**Participant:** Every week we used to have meetings prior to the COVID-19 thing. We used to have meetings every Monday. That's quality assurance.<br>**Interviewer:** Ok. So, what screening services do you provide as a district?<br>**Participant:** I am not sure of the tools they use for the procedure, but they have a static VIAC clinic here, then there are times when they go over the weekends to outside communities. They have a mobile clinic.<br>**Interviewer:** What VIAC screening guidelines are you aware of that direct the prevention and control of cervical cancer?<br>**Participant:** Like?<br>**Interviewer**: Which provides guidelines on when a woman should start screening, which age group is targeted, how often women should be screened, such things. So that the nurse is guided, and the woman is assisted with information.<br>**Participant**: Aah, we don't have. We might benefit if we are going to get it so that we can sensitise our clients so that they know when to get the services. But we tell them where to get the service. That is at the ANC clinic.<br>**Interviewer**: OK. You mentioned that you provide women with information so that they can get the services from the ANC Clinic. Are there other strategies which you use as a department to disseminate screening even beyond those admitted at the maternity unit?<br>**Participant:** Aah, I cannot say we really have a scheduled programme in relation to that. As a department, when we discharge our clients, we give them health information. At times we touch on

that but in passing since these clients are clients who have just delivered. But at times we sensitise them in relation to that.
Interviewer: So, what do you think are the reasons why screening services are not adequately utilised by the women, because we expect more women to be forthcoming than is happening now?
Participant: Do you want to know the challenges to us or to the clients?
Interviewer: Challenges to both you and the clients.
Participant: Right, ok. Unto me as how I see it, it seems to me that the programme is a new baby that is coming up. It might have been there, and I was not aware of it because from my own understanding when this thing started, I was hearing this thing is being done at Mpilo Central Hospital and stuff like that up to when it was disseminated to us. Now our hospital is also conducting it after people went for training. So being a new baby that has come, there might be lack of knowledge unto us the service providers as well as unto our clients who should be benefitting from it. So, there is need for us to increase information dissemination to our clients so that they become knowledgeable and make informed decisions. And also, culture, issues of culture.
Interviewer: Mmhh
Participant: The set up for the young generation. I cannot discuss the issue of sexuality and private parts. There we are talking about the cervical thing which others will say its taboo to discuss, stuff like that. Those cultural barriers coming in. That really needs to be tackled.
Interviewer: Are there any other barriers you can think of?
Participant: Silence
Interviewer: Ok, So, how can we overcome these barriers?
Participant: We need to increase education first so that people understand about screening. Then it has to be a multi-sectoral approach where we are saying mmm... we have a lot of programmes that are being offered to our communities and in our institutions. We need to collaborate these services so that where you talk about TB, cervical cancer information is also disseminated. If you are discussing about malaria, you also need to discuss about cervical cancer. That's collaboration of services. I think that will help. And we also need to continuously educate our clients so that they also tackle these barriers that are at stake to say despite the cultural barriers that are there, be sensitised and be knowledgeable on these things so that our clients will make informed decisions. Because its them or it's us. If we do nothing about our barriers, it might mean that the clients will suffer.
Interviewer: Yes, Yes. So, if I'm hearing you right, you are saying there are barriers pertaining to the health service and there are also those related to the clients.
Participant: Yes, and to the community related to socio-cultural norms.
Interviewer: Ok. So, if you were to be in charge of the VIAC programme at policy level, what would you do to make the programme more accessible to women? What improvements would you make?
Participant: Eehh, I would improve funding for the programme so as to improve the support system. Because for every programme for it to be functional, there should be resources to use and there is not enough funding provided for this programme. So, I would advocate for adequate resources, which can be manpower, which can be financial so that we have equipment and transport. Because for information to be conveyed from one individual to another, we also need IEC materials in local languages. There is need to have transport to ferry people from one end to another where information has to be disseminated. Yes. Adequate funding of the programme.
Interviewer: Thank you so much for this information. Could there be anything else you want to share?
Participant: I tried to give you as much as I know but maybe it's not enough. But you probed as much as you could.
Interviewer: Thank you so much for participating in this study. I will disseminate the findings at the conclusion of the study though the hospital authorities. Thank you again.

IDI 14
**Interviewer:** Thank you for participating in this study. It's still a continuation of the quantitative study where I was asking mothers questions and now, I would like to hear the views of the service providers on the cervical cancer screening programme in the district.<br>**Participant**: You are welcome ma'am.<br>**Interviewer**: Thank you. Would you please explain to me how you are involved in the cervical cancer screening programme, that is, the role you play in the programme.<br>**Participant**: As the sister in charge of the facility, the role I play is very important in that I have to make sure that the programme goes on well in this facility as programmed. I also ensure that the community is sensitised so that we have community participation, and they know what is needed of them and they know all the matters concerning their health to do with cervical cancer, because cervical cancer is a deadly disease. So, it is very important that our community is well informed as we are promoting the reproductive health concept.<br>**Interviewer:** Ok. So, how long have you been working at this facility?<br>**Participant**: I have been here for approximately 24 years.<br>**Interviewer**: That's quite a long time I'm sure with all the experience you have, you have a lot you can tell me. Have you attended some training on VIAC?<br>**Participant**: No, I haven't attended any training on VIAC.<br>**Interviewer**: You are in contact with women on a day to day basis in the course of your duties, in your own view, how knowledgeable are they about this disease and how aware are they about the screening programme?<br>**Participant**: Ah, my community is fully aware of this disease and the screening programme that is taking place because we do campaigns regularly using the hailer and also on our day to day contact with our clients, as they come to our clinic we give them health education talks on cervical cancer, and also we group our mothers through the health promoters in the different wards where they tell them about cervical cancer and also about the service at the clinic and at the hospital.<br>**Interviewer**: Would you be in a position to describe how prevalent this disease is in the district, or even in your catchment area?<br>**Participant**: Aaa, I am not sure about numbers per se, but cervical cancer is there in the community. This is identified through the mothers who go for hysterectomy and also who verbalise that they have cancer when we do our day to day consultation. But about figures I'm not quite sure.<br>**Interviewer:** Ok. What screening services do you offer for women in the district?<br>**Participant**: We provide static services and GPH does, but I am not sure about mobile services, but I know there are mobile services going on in the communities whereby there is a one stop approach where they do their outreaches. They also screen for cervical cancer.<br>**Interviewer**: You said you are not sure of the operations of the mobile clinic, but would you know who does this programme and how often they do these one stop programmes.<br>**Participant:** They are supposed to do it quarterly but because of funds and also transport challenges, as of now the programme hasn't taken of quite well.<br>**Interviewer**: Would you explain on who is responsible or who coordinating this programme? Is it the municipality of Gwanda or the Ministry of Health or other implementing partners?<br>**Participant:** It is the Gwanda Provincial Hospital, the district community health officers.<br>**Interviewer**: What guidelines do you use in the implementation of the VIAC screening programme and how feasible are they to implement?<br>**Participant**: We have the Zimbabwe cervical cancer prevention and control strategy document which has all the information on the screening procedure. It is very easy to follow.<br>**Interviewer**: What measures do you have in place to ensure that staff are aware of the screening guidelines?<br>**Participant**: We do our meetings every Monday and talk about all these issues including what we will do in the community. We also have monthly meetings where we emphasize on the programmes because we do integrated services at Phakama clinic.

Interviewer: You mentioned that the community is aware of cervical cancer and screening because you always do health education campaigns using the hailer system. Would you please shed more light about the hailer system?
Participant: Its whereby someone goes into the community using a loudspeaker to sensitise the whole community. They use a vehicle, and it reaches different wards in Gwanda including the outskirts. Yes, they stop even at bus stops, stores and other places where people are gathered, and they inform the community of Gwanda.
Interviewer: That's very innovative, and how often does this occur?
Participant: We do it quarterly and whenever there is a need. But as of now I remember it was thrice, that is every month. Three months following each other.
Interviewer: Could there be a specific reason for the increased frequency in the awareness creation?
Participant: We want to increase demand for the services, and also to take action where need be so that there is no one who is left unaware. Especially the mothers. It's very important that's why even with the HPV, human papillomavirus, we were targeting the girls and we disseminated the information likewise.
Interviewer: So, how is the uptake of screening and has there been an improvement in the attendance on screening seen through increase in the numbers in the past three months where the awareness campaigns were now more frequent?
Participant: Yes, there is a change. Mothers are coming. They are coming in their numbers. Yes, in such overwhelming numbers when we look at the staff available for screening. But the services are offered despite all that. Uptake of screening is now average.
Interviewer: How long have you been offering screening services at Phakama?
Participant: At Phakama, I think second quarter, somewhere there.
Interviewer: Ok
Participant: Yes, but it was on a lower magnitude. We were sensitising by that time.
Interviewer: We had a national target of 50% screening coverage by end of 2020, but we are still far below that. In your view, why do you think some women are not adequately utilising this free cervical cancer screening service? What could be the reasons?
Participant: I think its issues to do with this COVID-19, we cannot have many people coming to the clinic and also the myths and misconceptions that we cannot answer. And also, their socio-economic background. Some, even if the information has been relayed to them, they choose not to come. But this issue of Covid-19 is a challenge, yes.
Interviewer: You are bringing a very important issue of myths and misconceptions. What perceptions do women have about screening?
Participant: Some say that if you get screened and there is maybe something in cervix, it might blossom to cancer. So, it is better to leave screening, because if you temper with it, it might become full blown cancer.
Interviewer: Any other factors which may be contributing to some women's failure to be screened?
Participant: Here in Gwanda we are dealing with a mostly literate group, but others still choose not to come even if they hear the message. Some will say we are Christians, and we pray to God; we cannot go and have a check-up. It depends on their faith. The apostolic sect will say we cannot go to the clinic, we have our own hospital where we are fellowshipping, but I don't think these services are there. So, they end up not accessing the services at the clinics because of religious beliefs. Yaa, they are there. Others are just reluctant to come.
Interviewer: From the challenges which you have highlighted, what strategies would you recommend for dispelling these myths and misconceptions so that more women will understand the programme better and utilise the screening services.
Participant: I think we have to make sure that we approach the leaders of those religious sects and give the health education and emphasize the risk of the disease. Maybe one way or the other, they might turn and agree that mothers be screened. I think we have to be strategic and also health educate them holistically.

Interviewer: Thank you for the information. You have said all that you view as barriers to cervical screening in the district. In a nutshell, would you kindly summarise for me the barriers to screening in this district and your brief recommendations to rectify the situation on the barriers?
Participant: Ok. The barriers are these myths and misconceptions that are there, and also the Covid-19 that is here. Of course, it is deterrent now to the community, and also the illiterate group. There are some people who do not to screen because they are ignorant. Even though you health educate them, they will say these services are for those people with this and that. And also, our community is mobile, and people are out on gold panning and at times maybe this information might not reach them because everyone is up and about because of this Covid-19 thing which has paralysed the income. So, people are out there and might not see screening as very important. They might choose that; no, I'm okay, let me do my things and only a few might come. But I have also seen people coming in numbers which means they are now unleashing those myths and misconceptions since more are coming to access the service. In a nutshell I think this is what I can say.
Interviewer: Thank you so much for sparing your time. The interview has given me some insightful facts. Is there anything we did not discuss which you would like to comment on?
Participant: I just want to thank the programme managers for the screening programme because a lot of our women have been helped and they now know their status about cervical cancer. Reproductive health has been lifted because long back we would see lots of women with cervical cancer who would have hysterectomy done and they would die without their uteruses. So, it's not good. I for one I had hysterectomy done although it was for fibroids. But maybe I would have ended up having cervical cancer. If this programme was here long back, I think some hysterectomies were going to be unwarranted. So, I thank the programme managers for such a good service to women in general.
Interviewer: Thank you. We appreciate that. Another thing, how do you collaborate with other implementing partners? I was made to understand that the nurse who does the screening at this clinic is an employee of OPHID. How did you get them to bring someone to come here and work with you?
Participant: It was through the Ministry of Health that the cadre was brought along. The point was that after OPHID pulls out, the programme will remain a Ministry of Health programme and we would continue this service at the clinic like any other programme such as ART and VMMC. So, we will be doing all the services integrated and it will be a continuation because some of our staff members have been earmarked for training. Anytime they might go for training for VIAC. So, the service is going to, I think maybe clear the myths and misconceptions and everyone will have the service done, especially those who need it. This programme here is an extension of the Ministry of Health and OPHID is funding the human resources. But when they leave the programme, our nurses will have been trained. When OPHID pulls off, the programme will remain functional under the Ministry of Health like other programmes e.g. TB, VMMC
Interviewer: Thank you so much and we appreciate your support for research work.
Participant: You are welcome ma'am.

IDI 15

Interviewer: Thank you for agreeing to participate in the study which aims at improving access to cervical cancer screening by women in this district. I am interviewing health workers with different roles in the screening programme. May I find out how you are involved in the cervical cancer screening programme?
Participant: Eh- as a nurse here, I usually mobilise women for cervical cancer screening and we do give health information on the importance of cervical cancer and on possible causes of cervical cancer.
Interviewer: So how long have you been working at this station?
Participant: At this station I worked from 2009 up to now. It's been 11 years.
Interviewer: Have you received any training on VIAC?

Participant: No, they only train those who do the screening.
Interviewer: In your own opinion, how common is cervical cancer in your community?
Participant: Ya its common. It might be common because one other organisation comes to do cervical screening and they always get 2 or 3. They come once per quarter. But if it can be done regularly, I think we can have more cases of cervical cancer.
Interviewer: You mentioned that there is an organisation that comes to screen, which organisation is that?
Participant: It is being done by OPHID, but they usually do only HIV positive mothers. They do outreach once a quarter or so.
Interviewer: In your opinion, how knowledgeable are the women on cervical cancer? How much do they understand about cervical cancer and are they aware of the available cervical cancer screening services?
Participant: Ya, most of them are aware, because they come to a point where they come to the clinic requesting to be maybe referred to the next level that is Gwanda Provincial Hospital for cervical cancer screening.
Interviewer: You mentioned that OPHID comes to screen women on outreach, which other institutions or organisations provide screening in the district where you can refer clients to?
Participant: We usually refer them to Gwanda because it's the screening hospital.
Interviewer: How would you rate the screening status among women in your catchment area?
Participant: It's not many who have been screened, because some of them, because of distance, they are not able to go for screening. They cite money problems.
Interviewer: In your interaction with women on a day to day basis, what do you think their perceptions are on screening, and how would you rate their understanding of screening?
Participant: Ya most of them understand that it's very vital and it's very important for them to be screened for cervical cancer. I have heard women encouraging others to be screened because it is good to detect it earlier than later. One was saying "I myself have gone to Gwanda and have been screened and there is nothing to fear". Others were saying they have fear because they push in some instruments and so forth.
Interviewer: What guidelines do you have on VIAC screening protocols and procedures which also assist you as you educate women on screening?
Participant: We have a chart which we refer to looking at the stages of cancer, telling women what can be done at each stage and so forth.
Interviewer: You mentioned that you always educate women on screening and encourage them to be screened. What other strategies do you use to try and reach more women who are eligible for screening with information, apart from those who will come to the clinic?
Participant: To reach them, I think we need to integrate the services. Usually we do the EPI outreach, but if you can integrate that so that when they go out there you mobilise women so that they may be screened for cervical cancer. But at the moment we haven't done that, we just see them at the clinic.
Interviewer: So, what do you think are the reasons why women do not go for screening in the numbers which we expect because right now we are still far from the national target that had been set for 2020? We are trying to understand what makes women not accept screening.
Participant: I think the major issue is distance to Gwanda. People have no money to go there. The Community Health Workers also do not have enough information to teach the women in their places of residence on the importance of cervical cancer screening and the causes and so forth. I think as a Ministry, we need to strengthen or to give more information to Community Health Workers so that they can easily teach the mothers in their communities, such that they might come to the clinic and be referred to the level where they can get cervical cancer screening.
Interviewer: Could there be any issues related to culture which could hinder women from screening?

Participant: Yes, there are certain areas in the district with apostolic people who have myths and misconceptions but, in our area, we don't have that challenge.
Interviewer: From what I understand from you, the major barriers to screening are distance because screening is done in Gwanda, and that the knowledge might be lacking because the Community Health Workers might not have enough information.
Participant: Yes
Interviewer: So how do you think these barriers can be overcome?
Participant: I think for the first one of distance, I mentioned the issue of integrating services, where we can also give information about cervical cancer and screening at this level as we provide other services. We can also give information to Community Health Workers so that they can teach or give health information to the community about the importance of cervical cancer screening. That's how we can determine the myths or misconceptions and other cultural beliefs because we will be reaching every corner of the catchment area.
Interviewer: If you were in charge of the VIAC programme at policy level, what changes would you introduce to make the programme more accessible to women?
Participant: To decentralise the service and have primary health facilities doing the screening and nurses at this level being trained. I think we can improve the services because sometimes the partners can say I'm coming on such and such a date, somebody comes at 4 o'clock and they are already gone. But if it's at the clinic they know they are going there even if it's every Tuesday or every Wednesday or everyday they can be done cervical cancer screening.
Interviewer: Could there be anything else you would like to add which we didn't touch on?
Participant: Decentralisation could improve the uptake of cervical cancer screening services. And maybe if we could have flyers and pamphlets to give to the community, this could give them more information on cervical cancer screening, it would improve things.
Interviewer: How much access do you have to the pamphlets and fliers as you mentioned which you need to distribute to the community?
Participant: We do not have any of those currently.
Interviewer: Ok. Thank you so much for your participation in this study.
Participant: Thank you.

IDI 16

Interviewer: Thank you for allowing me to interview you for this study where I wish to get information on the barriers to cervical cancer screening in the district with the goal of improving the screening services. Could you please explain what role you play in the cervical cancer screening programme?
Participant: My role in this programme is to sensitise the women and telling them the importance of screening. Also, to reach the furthest distances in our ward talking about this programme. Even to mobilise them. The problem is on transport from these furthest distances. That is some people are willing to be screened but most have failed due to transport problems. Some are staying at a distance of about 30 km from here so it's very difficult to obtain the services, unless if there could be a team visiting them.
Interviewer: You talked about sensitising the community about the programme and you also mentioned problems with transport.
Participant: Yes.
Interviewer: So, how do you manage to reach them given the transport problems?
Participant: Aah, they are coming, some they are coming to the clinic. Those who can manage to come they are coming. If they are given the dates in time they do come.
Interviewer: So how long have you been involved in this programme, or rather how long have been at this clinic?
Participant: I have been in this clinic since it was opened in 2000.
Interviewer: Oh, so you are the founder, nice. Have you received any training on VIAC?

Participant: Ah, no, no, I haven't received any training on that. But there is one member of staff who is trained in that.
Interviewer: Oh, so you have a nurse trained in VIAC in this clinic? Where were they trained?
Participant: I can't remember but I think it was in Bulawayo
Interviewer: Was the nurse trained when they were already working here?
Participant: Yes.
Interviewer: So, what are the community's views on cervical cancer as a disease and what are the community's perceptions on cervical cancer and the screening programme?
Participant: We have seen many cases of CC. Some people believe that cervical cancer is a disease that was here long back which does not require modern treatment. They believe that it can be treated traditionally. But some believe that if you visit health centres you can get help.
Interviewer: You have mentioned that you are doing a lot of sensitisation about the programme, so in your opinion how aware are the women in your catchment area about screening and how would you rate their knowledge levels?
Participant: More than less women know about the programme because of sensitisation. We always tell them. Most women believe that to be a woman you have to apply some herbs into the genitalia when they are having sex. So, by sensitising them and disseminating the information they now know that it's not good to apply such herbs because those herbs are the ones which can cause the problem. At the same time, we tell them that that if they apply some medicine in the vagina, when you are performing sex, nature takes part, it has got that lubrication. The herbs absorb that lubricant, then it would be hard for the penis to penetrate resulting in bruises which causes cervical cancer.
Interviewer: Ok. So, where in the district do women get screening services.
Participant: Within the Ministry of Health there is a team which is mobile, a mobile team, they used to come this side. They tell us to mobilise then they visit us. Then they meet and screen the women.
Interviewer: How often do these services come to you?
Participant: They used to come even after 3 months but not always.
Interviewer: When was their last visit?
Participant: I think last month they were here.
Interviewer: Ok, how many on average do they screen at each of their visits?
Participant: I cannot say the actual number, but sometimes it can be above 50 women.
Interviewer: At your facility, do you have screening guidelines which gives direction on VIAC screening protocols that also assist you in the dissemination of information to the community?
Participant: Ah, can't remember but I think it should be there. *Calls out to another nurse and asks if they have such guidelines and the nurse replies that they do not but highlights that they have a VIAC register which is used by the outreach team when they come.*
Interviewer: In your opinion, why do you think there is low utilisation of screening services by women than we expect?
Participant: Right, there are some religious groups like the Madida sector who do not attend health services and allege that they can religiously treat cervical cancer. They will not come for screening. Then some fear that some other infections will be detected or those who are not yet prepared to be tested for HIV are afraid that maybe they will be found to be HIV positive. Then some are ignorant they do not have full information of what is happening. They just hear that there is this screening programme, but they do not understand the importance of it. That of distance I have already mentioned. Some fear the partners I am trying to say the spouse, the husband. If it can be detected that she has cancer, if she had not told them about the programme, how does she discuss it should she have a VIAC positive result. She won't even be given money to go for treatment. So those are the other barriers.

Then without looking far, even we the health workers, we always have patients coming to us from catchment areas of other clinics for treatment. When we ask them why they do not go there, they tell us that nurses there treat them badly. Maybe our own patients say the same about us in other

clinics. So, bad attitudes of the nurses could be another barrier because we also do not know how the outreach team treats them.<br>**Interviewer**: Ok. Does the outreach team have the capacity to screen everyone who wants to be screened?<br>**Participant**: I don't know but normally they target the PLWAs and turn the others away. Then if those are few, I noticed they said for their next visit even those who were not PLWAs could come, but only a few came. What I also noticed is that the outreach team comes late. By the time they come some people who stay far would have left. So, next time only a few people will come. Failure to keep time is also a barrier.<br>**Interviewer**: Given all these barriers which you have highlighted, how best do you think these barriers can be overcome? What would be your recommendations to facilitate better access to screening by women?<br>**Participant**: Right, one, punctuality of the screeners. Two; attitudes of the staff towards the clients should change. Still on staff, male nurses should discuss with men wherever they meet about this issue of cervical cancer. For example, I play draft, so I should discuss the programme when I am there so that some of these other men might take it into consideration. Then it will be easier for them to support their spouses. That way we can improve acceptance.<br>**Interviewer**: The issue of distance where you said women may not have the money to go to Gwanda for screening how can we address that?<br>**Participant:** The outreach coming from Gwanda should maintain their set time. The problem is punctuality. They may set time for the morning, then they come in the afternoon. This is a problem for people staying far from the clinic who end up leaving before they get the service but if they come on time the challenge of distance is reduced because people will travel back to their homes when it is still light. And they should know that communication is important. If they come with stories of vehicle breakdown when they did not communicate, next time people will not come back.<br>**Interviewer**: Ok. Is there anything else that you would like to comment on that we did not talk about?<br>**Participant:** No, but if you have something to say to us, we will be happy.<br>**Interviewer**: Today I just wanted to hear what you as health service providers view as barriers to cervical cancer screening. The information you gave me will help answer the question and hopefully contribute towards improving the cervical cancer screening programme in the district. Thank you very much for being part of this study.<br>**Participant**: Thank you, you are welcome.
**IDI 17**
**Interviewer:** Thank you for agreeing to participate in this study which seeks to determine the barriers to cervical cancer screening in the district. Your opinion on the cervical cancer screening programme is very important as it could contribute towards improving delivery of screening service.<br>**Participant**: Yes.<br>**Interviewer**: Could you please tell me your experiences on the cervical cancer screening programme, how you are involved or what your role is?<br>**Participant:** Ok. My role here is to eehh, when the mothers come here, we give health education about cervical cancer. We have got a chart where we show them that every year they are supposed to be screened. When they are not screened, the cancer will advance. We usually refer them to Gwanda for screening, but few go. Sometimes the nurses from Gwanda Hospital will come here to screen. But they usually target those who are HIV positive mostly. So those who are HIV negative, for them to travel to Gwanda to be screened, the challenge is on money because these days they charge in Rands and US Dollars, so the challenge is about money.<br>**Interviewer**: Ok. So, how long have you been stationed here?<br>**Participant**: I have been working here for 12 years now.

Interviewer: Oh, I ‘m sure you now know your community very well. In your opinion, how knowledgeable or how much knowledge does the community have on cervical cancer and what are their beliefs and views about the disease?
Participant: The knowledge is very little. Some don’t want their wives to come according to their cultural beliefs. For some it’s the church. We have got the Madida sector. Their religion does not allow them to use health services, so they are afraid to visit the clinic during working hours. So, those who sneak out, they do that late and we have to attend to them during late hours. So, it is a very big challenge to refer those cadres to Gwanda hospital for screening. For example, if we get a mother who is HIV positive, we give them ART when most people won’t be around to see her. So, for cervical cancer screening now, it’s difficult for them to go to the hospital because they will be seen. So, for the outreach team to include those who are HIV negative would help. But usually, they target the HIV positive mothers only. That’s the challenge we have.
Interviewer: Ok. How common is cervical cancer in your catchment area?
Participant: I don’t think it’s all that common. There are just a few especially gauging by those who are referred to Gwanda after screening by the outreach team.
Interviewer: I heard you mention some socio-cultural and religious beliefs. How prevalent are those beliefs in the community?
Participant: There are many who will say their husbands don’t allow them to be screened, and those forbidden by their church are also many.
Interviewer: Would you like to comment on women’s understanding and beliefs of cervical cancer screening?
Participant: They do understand, I believe *those who are interested understand* because we give them health education that they are not supposed to put herbs in their vagina. We tell them that there is no need to put any herbs to tighten their vagina in order impress their husbands. This is very dangerous. We tell them everything is ok with the vagina. It’s supposed to be moist. So, they will tighten using the herbs so that it becomes dry. So, during intercourse once somebody has bruises it’s dangerous. And also, to avoid having so many partners. That can predispose them to get cancer.
Interviewer: I hear that you give a lot of health education. Are you confident about their understanding and knowledge they have on cervical cancer?
Participant: I think they have the necessary information but not sure about the understanding part.
Interviewer: How do you reach those who do not come to the health facility like the religious groups you mentioned?
Participant: We liaise with the Community Health Workers. Even when the Environmental Health Technician is going out for the domiciliary visits and sometimes with one of the nurses, they also talk about it, the cervical cancer.
Interviewer: Ok. You mentioned a chart which you use to teach women about cervical cancer. Would you have other guidelines in addition to the chart which give your direction on how to mobilise and educate women on VIAC screening?
Participant: No, we do not have any guidelines. I only asked for that chart from Gwanda hospital so that when we do health education, we also show them the stages of cervical cancer. So, we don’t have the guidelines. It was my initiative to have that chart.
Interviewer: Has anyone in this facility been trained on VIAC?
Participant: No, none of us have been trained.
Interviewer: In your opinion, why do you think women are not utilising the free screening services to the level we would want them to apart from what you have already mentioned?
Participant: In my own view, the biggest challenge is that the outreach team only screens HIV positive mothers which excludes those who are HIV negative. So, those who are HIV negative are referred to Gwanda Provincial Hospital, and the challenge is the money for transport since the transporters now want US dollars or Rands. That’s the biggest challenge.
Interviewer: Apart from the transport and socio-cultural and religious factors, could there be any other challenges which could contribute to women failing to access screening services.

**Participant**: Some say the distance, even when outreach services come to the clinic, distance from their homes. Some say they are too busy and don't have time to go for screening.<br>**Interviewer:** Since you said you have not received any training on VIAC, are you satisfied with the knowledge that you have to effectively deliver information to women covering all aspects of the disease.<br>**Participant:** I think if we could have guidelines, we can read to gain more knowledge before we are able to go for training. Some things we are not sure of.<br>**Interviewer**: Given the challenges that you mentioned, how do you think these barriers can be addressed so that more women can access the service?<br>**Participant**: The outreach team, should open screening to everybody, not just the HIV positive women. When the outreach team comes, they only do screening at the clinic. Maybe they could allow us to increase coverage by mobilising women in villages far away from the clinic so that when they come, we take them, and they screen at that village. This would address the problem of distance. Maybe it would be difficult in terms of space I don't know. But we can liaise with the schools for venues and use the classrooms.<br>**Interviewer**: How often does the outreach team come?<br>**Participant**: Here, its maybe once a year.<br>**Interviewer:** In your opinion, if they were to come more often do you think more women would ……<br>**Participant**: Be screened? Yes, more women would be screened. They should make it quarterly if possible. Because when they come the turnout is good. They work from morning up to 6.00 pm. They even run out of sterile equipment and we even have to autoclave for them here. Yes. Because we really mobilise. If they tell us we are coming, we really mobilise so that women come.<br>**Interviewer**: And those are just the HIV positive ones?<br>**Participant**: Yes. I don't know why they do HIV positive only. They should screen everyone. But sometimes if the HIV negative ones come, they also screen them. Last time they came here I was also screened (laughs). But I was afraid.<br>**Interviewer**: You are raising a very interesting point, why were you afraid? Could fear be one of the reasons why women do not come for screening?<br>**Participant**: Definitely. If I was also afraid of pain during the screening and the outcome, it could be the case with them. I was happy to be screened. It helped me.<br>**Interviewer**: If the outreach team comes and they screen and run out of packs, in your own opinion do you think they have the capacity to screen everyone who wants to be screened?<br>**Participant**: They have few instruments. Maybe they need to get more. Us also, maybe we need to have some so that we sterilise them and keep them ready for when they come. But where do we get funding for that. They have to get more equipment.<br>**Interviewer**: What happens to those who test VIAC positive? How do you ensure that they receive treatment?<br>**Participant**: The women are told to come to the Hospital to see the gynae there. Some who tested positive last time failed to go to Gwanda because of lack of money. I think it is better for the gynae to come down and treat women in the clinics where they were screened. It would be much better for the women.<br>**Interviewer**: Is there anything else we did not talk about which you feel it's important for us to discuss?<br>**Participant:** I think we have exhausted all I had to say.<br>**Interviewer**: Thank you very much for your participation.<br>**Participant:** Thank you so much, hope the findings will assist our community.
**IDI 18**
**Interviewer:** Good afternoon and thank you for participating in this study whose goal is to determine the barriers to cervical cancer screening in the district so as to help develop strategies for improving the delivery of the screening service.<br>**Participant**: Good afternoon and thank you.

Interviewer: Working in this facility, could you kindly explain to me the role you play in the cervical cancer screening programme.
Participant: At Stanmore clinic actually, we don't do the screening, but we only teach the women, looking especially at the HIV positive women. We normally refer those with lower abdominal pain and vaginal discharge to Gwanda Provincial Hospital for screening. We usually mobilise women for screening by the outreach programme. But as of 2020 we did not have the outreach team coming to our facility. But usually when they come, they would be expecting to find 35 women who are aged between 24-45 years who are HIV positive. That's when we mobilise these women, and the turnout has always been good. So, our role is health education and mobilisation for screening. So, Stanmore clinic relies on the outreach team for screening, otherwise for most of our clients we refer.
Interviewer: So, who is the coordinator of the outreach team? Where are the staff coming from?
Participant: The staff will be coming from Gwanda Provincial Hospitals together with the District Nursing Office's team usually.
Interviewer: What is the justification for limiting screening to just the HIV positive women?
Participant: When we mobilise for screening, the HIV positive women are a priority of course as we consider them as the high risk group, but we also consider the HIV negative. When they are having their outreach programmes, their first priority is to screen those that are targeted, then after that they also screen the HIV negative women as long as they are there and willing to be screened.
Interviewer: So when you mobilise for the outreach team is it open for all women? I heard as if you said they screen a certain number. So, if the HIV positive reach that number, how do you deal with the HIV negative women?
Participant: All the women who come to the facility are screened. They can be more than the required number, but all are done as long as the packs can accommodate them.
Interviewer: Have you received any training on VIAC, or has any nurse from this facility?
Participant: No.
Interviewer: How long have you been working at this facility?
Participant: Eleven years.
Interviewer: I'm sure you now know your community very well. Could you just comment on the prevalence of cervical cancer in your catchment area, and how much knowledge the community has on cervical cancer and screening?
Participant: The community yes, we usually have challenges on the uptake of these new programme. But I can safely say as of 2018, that's when women got to understand about cervical cancer screening and coming to 2019, there are women who come inquiring about screening. Then because we don't provide that service, we refer them to Gwanda Provincial Hospital. As I am made to understand, there is going to be a mass screening campaign at Gwanda Provincial Hospital soon. We also capitalise on those days. So, we are trying to sensitise them as much as possible to go and access that service. But looking at our community, yes, the prevalence of cervical cancer may be high because we realise by the majority who have to be done the chemotherapy part of it and *women still need more education on this disease and the importance of screening to have better knowledge.*
Interviewer: So, you mentioned that screening services are available at Gwanda Provincial Hospital, are there any other places or organisations in Gwanda district where women can access screening.
Participant: Yes, there is also Phakama Polyclinic. Those are the two.
Interviewer: What guidelines do you use to assist you in the sensitisation and education of women on screening?
Participant: We don't have the guidelines as such, but we have posters. We actually have one poster that has pictures of the cervix according to the stages of cancer. That's the chart we use to educate our women.
Interviewer: Ok. You mentioned that you do a lot of education to women on cervical cancer and sensitise them for screening. What other strategies do you use to reach women who are eligible for screening with those messages, particularly those who do not come to the facility.

Participant: We utilise our Community Health workers. We also utilise our Health centre committee members since they are the ones who are the linkage between the community and the clinic. At times we do line listing so that in case we have a mobile team coming to our facility, we already have the existing line listing. Those who are in need of screening as a matter of urgency, those are the cadres that we refer to Gwanda Provincial Hospital.
Interviewer: So, in your opinion, why do you think women in the district in general or specifically in the community you serve do not adequately utilise the available free cervical cancer screening services?
Participant: I believe it's lack of knowledge and even acceptance of the service, and the money they have to spend to go to Gwanda for screening.
Interviewer: Talking of acceptance, how do women perceive cervical cancer screening, what are their beliefs on it which could be a hindrance for them to accept screening.
Participant: Women usually tell us that they are afraid of the procedure, especially being packed. That's what we normally hear them talking about. And even the procedure itself, they don't like to be fidgeted around their genitalia.
Interviewer: Could there be other socio-cultural and religious factors which could be influencing women's decision not to screen?
Participant: The challenge that we often see is those who stay with their mothers in law. They are the women who come later in need of such services telling you that they couldn't manage to come earlier in fear of explaining the reason for visiting the facility to their mothers in law. But otherwise we don't see much of socio-cultural factors that could hinder women from screening.
Interviewer: You mentioned fear of the procedure as one of barriers and you also mentioned lack of knowledge and that screening is only done in Gwanda town. So, how do you think these, and other barriers can be overcome so that more women are forthcoming?
Participant: Maybe those mobile teams could come more frequently, not only once after 6 months or so. If they frequently come maybe every month or every 2 months, *maybe the women would end up getting used and share the idea of screening with others.* Maybe one or two especially those who would have got treatment can actually confess to say no, the service is actually good, and this can actually help others to come.
Interviewer: So, when women are screened and get a positive result, how easy is it for them to go to Gwanda Provincial Hospital when you refer them for other treatment which cannot be offered during screening such as LEEP? Is a follow up mechanism which you apply to ensure that the woman has attended and is not lost to follow up?
Participant: Mmhh, when they receive a positive result, at times we realise that after having been told of their positive result by the outreach team I don't know whether it's the fear of those nurses or maybe they will be too busy, they usually come to us as we are used to them. Then they start asking again, what does this mean? This shows they didn't understand or maybe they still need further understanding to say if I'm told that I am positive, what are the implications, what is going to be done to me there at Gwanda Provincial Hospital. Yaah, even to go back home there and explain to their partners about this positivity issue that they have been informed about becomes a problem. So, when we explain to them, they understand it, *it actually takes time for them to go to Gwanda Hospital.* At times it can be financial issues yes, but at times it will be that they don't understand. So, it could be an issue that during their education sessions before they are screened, they didn't get much knowledge or time was too short for explanations. But finally, they do go though we don't have the list.
Interviewer: How long do women have to wait from the time they arrive up to when they are screened, that is when the outreach team comes to your clinic?
Participant: Usually the team doesn't delay. By 08.30 or 09.00 they are here, and the women start pouring in later. So, they give health education to those who are there and screen. After that they take another batch and so on.

Interviewer: What recommendations would you make for the VIAC screening programme to be more accessible to women?
Participant: Yes, knowing that there are financial challenges in the Health Ministry here and there especially for this programme, and taking into consideration that Gwanda is a large district, catering for Gwanda North and Gwanda South, maybe if they could deduce a system to have 2 mobile teams, one going to Gwanda North and another covering Gwanda South. Like I said before that its Gwanda Provincial Hospital and Phakama clinic who are offering these services, they should do a schedule which ensure that every month at least every clinic is reached with screening services with one outreach team looking on the northern side and the other on the south. I think then maybe we could cover more women.
Interviewer: Could there be anything else you would want to add?
Participant: Like you asked before if we are able to trace our women who test VIAC positive, we noted that when the mobile team comes, as a facility we don't have those registers. So, the mobile team brings their own registers, they screen our clients, then they go back with that register. But maybe they should deduce a system whereby the facility also has its own register; they attend to those clients then we as a facility are the ones who are supposed to report those women. They have to make it a bit easier for us to know who tested positive so that follow ups are done on those women and we be the ones who compile those records.
Interviewer: Thank you so much for your participation. Your contributions should help improve the cervical cancer screening programme. Findings of the study will be disseminated through the district health authorities.
Participant: Thank you.

IDI 19

Interviewer: Good afternoon and thank you for sparing your lunch hour to talk to me. I would like to find out your views in relation to the cervical cancer screening programme in this district. Kindly explain to me how you are involved in the programme.
Participant: As women, on my side I was very happy to hear about this programme of cervical screening because there are so many of our relatives in the past who died of cervical cancer. So, this programme is very helpful. Especially these young women, we encourage them to come for screening.
Interviewer: As a Health Promoter, what is it exactly that you do in your community to support the screening programme?
Participant: We have a borehole in the community since we do not have piped water yet in our township since it is a new one. This is where women get their water from. So, when women come to collect water, I wait for them there and that is the first thing I preach about. I tell them that ladies, let us go for screening. They are screening at the clinic, and you don't pay anything. And for sure many have gone and been screened. But it is not all of them who agree, many have not been screened. Others still believe in their old traditions. They say I will go to the prophets; I will go to the traditional healers and they will help me. Some will say 'the metal thing they insert is painful etc etc". There are so many things they mention.
Interviewer: How long have you being in this role of Health Promoter?
Participant: It has been 3 years now, yes 3 years.
Interviewer: Ok. In your own assessment, how prevalent is cervical cancer in your community?
Participant: Mmmmh, it is there in the community. *There is plenty of it because I have seen people who have it.* And also, women sometimes will tell you that I don't feel well then, I encourage them to go to the clinic. When they come back, they don't hide from me. They will tell me they were told there is something wrong in their cervix and they have to go to Gwanda Provincial Hospital to see the doctor.
Interviewer: How much knowledgeable do you think women have on cervical cancer and the screening programme?

Participant: It seems as if the women *have got a bit of knowledge, but they still need more.* A lot of it. There should be more awareness campaigns focused on women where they gather in different places. Even at churches because women are always asking a lot of questions such as what causes the cancer, how can one tell they have it etc. So, this shows that people don't know.
Interviewer: Mhh
Participant: So, there is still need for awareness campaigns especially in churches because almost all women go to church. And at the boreholes around 3, 4 when women come in their numbers, they should be taught there. Sometimes when women are taught by us who stay with them in the same communities, they think there is something I am campaigning for at the clinic and don't take me seriously, not knowing that I am just helping their lives.
Interviewer: How do you suggest these campaigns should be done in a way that will be acceptable to all women?
Participant: I think workshops should be conducted for women to be taught about this disease.
Interviewer: Apart from workshops could there be any other ways which could be used to reach more women?
Participant: The other methods are those which I have already talked about. They should be taught in churches because they are the majority compared to men. They should start by talking to the Pastors and explain the programme. Clever women should then be identified from each church who will be trained to deliver the messages every Sunday and encourage women to go for screening.
Interviewer: Ok. So which places here in Gwanda district provide screening services?
Participant: Here at Phakama clinic it used not to be there but now it's there, then Gwanda Provincial Hospital which has been screening for a long time.
Interviewer: What written down guidelines do you use when sensitising women for screening. With information such as when a woman should start screening and how frequent they should be screened?
Participant: The nurses tell us that *women should be screened every year.* We have also been taught that women should not insert their fingers in the vagina when bathing because their nails can scratch the cervix which puts her in danger of having cancer.
Interviewer: You have just mentioned what you were taught. Was that during the training you received on cervical cancer and screening?
Participant: No, we have never gone for training related to cervical cancer. We are involved in many programmes such as nutrition and TB. So, when I go to them with a nutrition or TB programme, I also put in something on cervical cancer and encourage women to go for screening. But no, we have never received any training on cervical cancer. We learn about the programme on the job.
Interviewer: Many are not taking up the screening programme at the rate that is expected even though screening is now offered right here at Phakama clinic nearer to them. What do you think could be the reason for this low uptake of screening?
Participant: I think churches also contribute. And the other thing; women are afraid that if I go for screening then I am told there is a growth in the cervix then my uterus will be removed. Then I won't be able to have a child and this will affect my marriage. Especially the young women. They will ask you "what will I do if I go there and they remove my uterus". It becomes very difficult for me to give an answer because if the doctors come to the decision of removing it, it means they will have seen the damage can't be treated any other way.
Participant: Could there be any other reasons?
Participant: The other thing is the queues. As you can see that our clinic is small, and the nurses are few. Women don't want to come here and spend the day in a queue. Others also don't want to be seen in the queue because she is afraid people will think she has cancer. I really don't know why they are bothered about all that. And these young women, they are afraid of their husbands. If they are screened and test VIAC positive they are afraid what the husband will say. They will be afraid that they will lose their marriage because the husband will ask where she got the disease from.

Interviewer: You mentioned churches at some point as the reason why some women don't want to be screened. Could you please elaborate on that.
Participant: There are some churches which do not allow their members to go to hospital. Even for delivery they deliver in their homes. So obviously they are also not coming to be screened because it's against their faith.
Interviewer: How common are these churches in the district.
Participant: Ah they are many. These are the Apostolic churches. They don't come to hospital.
Interviewer: In your own view, what could be done to ensure that the screening programme is accessible to all women?
Participant: My recommendation is that this education on cervical cancer and screening should start with the community leaders. Be it church leaders or councillors and headmen. When these leaders have received information, they will go out to their people and spread the word in the areas they are leading. People listen better to their community leaders because they respect them.
Interviewer: Any other recommendation?
Participant: I think queues at the clinic could be reduced by having mobile screening clinics which go out to busy places in the ward such as in shopping centers to screen women there. If women get to know about mobile clinics, they will come because they won't wait a long time. Besides, I think women like it when people come to them. They don't want to be the ones going to seek the service. Let us not only rely on Phakama and the Hospital alone, they are too few to attend to all women. Let us increase the service by introducing mobile clinics. Women give many excuses for not screening.
Interviewer: Could there be anything else you would want to comment on?
Participant: The other problem; men. Especially the young husbands. I wish there could be lessons which combine both men and women so that after men have also been educated, when they are now home sitting down with his wife, he will ask her if she has gone for screening. They will remind each other.
Interviewer: Anything else you would want to add?
Participant: No, I think I have said all I can remember. Thank you for including me in the study I hope this has been of help.
Interviewer: Yes, it has been nice to hear your views which will help me better understand the barriers to cervical cancer screening in the district. Thank you for your participation

1D1 20: HEALTH PROMOTER: SENONDO WARD (URBAN)

Interviewer: Thank you for participating in this interview. We are still continuing with the same study from last year where we targeted women and asked them questions related to the cervical cancer screening programme in the district. This time we are targeting health workers with different roles in the VIAC screening programme. Could you please explain to me your role in the cervical cancer screening programme and how long you have been a Health Promoter.
Participant: I have been a Health Promoter for 4 years. My role is to sensitise women in the community to go for cervical cancer screening as early as they can. I also give them information related to cervical cancer. I tell them that if they delay, it may be dangerous to their health because should they be having cervical cancer, it will be progressing.
Interviewer: Seeing that you educate women on cervical cancer and encourage them to screen, have you received any training pertaining to cervical cancer and the screening programme to empower you with information to disseminate to the women?

Participant: As a Health Promoter, I spend a lot of time at the clinic. Women in the maternity unit and those who come for HIV testing and collection of monthly supply of their ARVs are always given health education by the nurses. I also listen when women are being taught and that is how I have gained knowledge about the programme. When I am in the community and at community meetings, whenever I meet with women even at church, I encourage them to go for cervical screening. Especially those who are HIV positive, they should be screened every year and those who are HIV negative every 3 years. I have not received any formal training on cervical cancer and screening. The knowledge I have is that which I get as nurses are teaching patients. So no, I have not had full training. I wish to get some training on VIAC
Interviewer: In your opinion, how prevalent is cervical cancer in this community?
Participant: Ah, I think the disease is very common here because mothers insert herbs inside their vaginas which they are not supposed to. I think this cancer is very common.
Interviewer: Would you please comment on the level of knowledge women have on cervical cancer and the VIAC screening programme according to your observations in your interaction with the community.
Participant: I can say half-half. Some know, they have the knowledge, others still don't have full understanding on these matters concerning cervical cancer. You can tell from their comments when you talk about it that they are ignorant.
Interviewer: Where can women access cervical cancer screening services if they want to be screened?
Participant: Services are offered at our local clinic here at Phakama and also at Gwanda Provincial Hospital and they are free.
Interviewer: Are women aware of these screening centres?
Participant: Most are aware and for those who are not, we tell them when we are sensitising them for screening. From 8 am – 3 pm, they will be screening at Phakama clinic.
Interviewer: Would you know when Phakama started offering screening services? When I came for the first phase of the study in 2019 screening services were not available at this facility.
Participant: I think it's about 2 months or 3 months back I am not quite sure. But this has helped a lot because more women are coming because it's now nearer to them than going to the big hospital.
Interviewer: How keen are the women in your community on screening and what are their perceptions about screening?
Participant: Ah, in my community, even if you tell them about screening, they still have that ignorance and are reluctant to come. Why, most of them will tell you I am afraid if I am told that I have cervical cancer a lot of things will change in my life and I will live in fear of death, so I won't have a good life anymore. So, it's better not to be screened and avoid stress. But knowing they know because even if you go to the clinic there are posters everywhere with information on cervical cancer. Most women know about the screening programme because I always tell them that they should go for screening.
Interviewer: Do you have written guidelines on when women should start screening, how often they should be screened and when they should stop screening to help you as you give information and sensitise women for screening?
Participant: This programme mostly targets HIV positive women because they are more at risk to catch cervical cancer and they are screened every year. Then for the rest of the women they are screened after every 3 years. But no, we do not have any written down guidelines to follow. It's the information we get from the clinic when nurses are teaching and from the posters at the clinic that we see.
Interviewer: You mentioned that you always hold discussions with women and motivate them for screening. How and where do you reach these women with screening messages and how often do you meet them?
Participant: Especially when women are here at the clinic, I spend the whole day at the clinic 3 times a week. When I am at the clinic, I talk to them as they are in the OI queue and sensitise them for

screening. Usually they agree and I take them to the screening room. It does not take time there; about 15 minutes they are done then they go back to take their place in the queue. I also visit women in their homes to encourage them to be screened, especially those who are working and spend the day at work.

Interviewer: Ok. So, the screening programme is available and now even offered at the local clinic, in your own opinion, what reasons could there be which make women not come for screening in the numbers that we expect?

Participant: Women are just reluctant to go to the clinic and some are ignorant, they do not fully understand why it is important to be screened. But we try to tell them that the earlier the better because if screened early, if you are found to have a problem you can be assisted early before complications occur because it progresses to cancer which can no longer be treated.

Interviewer: Could there be any other challenges which hinder women from screening?

Participant: Women are just lazy to stand up and go to the clinic and some will tell you they are *too busy making a living* because you see, most women here are vendors, so they say by coming to the clinic they lose money.

Interviewer: If you were in charge of the screening programme, what would you do to ensure that all women have access to the screening programme?

Participant: I would *increase awareness creation campaigns to maybe 3 times a week using a hailer to keep them reminded about the screening.* Currently it's done every month. May be repeated reminders can motivate more women to be screened because they will tell themselves why is this thing being repeated so much, it means it is important.

Interviewer: Anything else?

Participant: There are some people who never set their foot in a hospital. They believe cancer things are caused by witchcraft, so they choose to go to prophets and traditional healers for advice. Yes, that's what others say. They say you don't go to hospital for cervical cancer, so those people won't come for screening. They will visit traditional healers for treatment when they are sick. The traditional healers are there here. They say if you go to the hospital and you are screened, you are starting trouble for yourself because once touched, the cervix will start to be always painful and start having a disease which will grow out of control. That is another barrier.

Interviewer: Could there be any other barriers or recommendations you have which could enable more women to access screening?

Participant: No, the most important thing is for us to continue encouraging women and also to add onto the knowledge they have so that they can have more understanding because knowing about the screening services, they know.

Interviewer: Thank you very much for participating in this study and for helping me recruit the participants for the Focus group discussions.

Participant: Thank you to you too.

1DI 21: VILLAGE COMMUNITY WORKER: WEST NICHOLSON

Interviewer: I would like to thank you for your willingness to participate in this interview. As a cadre who is always in contact with your community, please tell me what role you play in the cervical cancer programme.

Participant: My role in this programme is to teach the women and encourage them to be screened for cervical cancer.

Interviewer: How long have you worked in this capacity?

Participant: I think this is the sixth year.

Interviewer: Have you received any form of training related to this programme?

Participant: I have not received that training as yet. But when we are at the clinic the nurses tell us that we should mobilise women and encourage them to go for screening. But I have not attended any training specific to the screening programme.

Interviewer: In your own view, how common is cervical cancer in your community?

Participant: I have not encountered any woman with cervical cancer in my area, so I think the disease is not much of a problem here.

Interviewer: How would you rate the level of understanding of cervical cancer and the screening programme among women in your community?

Participant: What I realise is that women have not yet received enough information on cervical cancer which would make them fully understand the importance of screening. I think that is why many have not been screened. What I have noticed is that they have little understanding of the disease. It would help if we could be visited by other people to educate them about this disease.

Interviewer: Pertaining to screening, how would you rate women's knowledge and level of understanding about the programme?

Participant: They know because I always discuss the programme when I am with them. They may not fully understand, but they are aware of the screening programme.

Interviewer: When discussing these issues with women, what tools do you use which guide you on the information you give pertaining to when a woman should start screening, how frequent they should be screened etc

Participant: That's where the problem is. I can teach them the little I know but we do not have any straight guidelines to refer to. I am not so sure on the full information I should be giving them because I do not have anything written down to follow. But we have the information which we learn from the nurses.

Interviewer: You mentioned that you educate women on cervical cancer and encourage them to be screened. What methods do you use to reach these women with information?

Participant: What I normally use is that when I meet the women when they bring their babies for weighing, I always remind them to go for screening. I tell them that if they are not screened, they may develop cancer and it will not be detected and by the time you get sick, it would be too late for you to get any help then you may die. That's the opportunity I use when I meet them to tell them about screening.

Interviewer: Where do you meet the women for weighing their babies and how often do you meet?

Participant: I weigh the babies once every month. We have a community meeting place under a tree. That's where I meet them. I weigh them at community level, not at the clinic. Only those whose babies are due for immunisations are the ones who go to the clinic.

Interviewer: In your opinion, what do you think are the reasons that make women not come for screening?

Participant: What makes women fail to be screened is that screening is only done in Gwanda. People have problems of money for transport to Gwanda. This is the major reason. Because of distance to Gwanda where screening is done.
Interviewer: Apart from distance, could there be any other reasons for women not to access screening services?
Participant: If it was possible, it's better to have screening also done at local clinics so that mothers don't travel all the way to Gwanda. This would help a lot because women would then access services at their convenience without having to pump out money from their pockets to travel to Gwanda. More education of mothers is also required so that mothers get more information on this disease. Lessons should be organised and preferably given by people coming especially for that. If the women get full information, it makes it easy for them to accept screening.
Interviewer: I hear you say people should come and give mothers education. Who should give this education and how should it be delivered to make it acceptable to the women?
Participant: A team should come from Gwanda Provincial Hospital and give education on VIAC where the local Community Health Worker will also be present. I say that because those nurses have full information. I personally don't understand some of the things so if those with full knowledge come it would be very helpful to both me and the women because I will also learn some things.
Interviewer: Ok. What are the community's perceptions on cervical cancer? Here I am trying to dig for more possible reasons which make women reluctant to screen.
Participant: The problem that is there is that women do not yet have full understanding of this disease and the reasons why they should be screened. I don't see why there should be a problem if the community is well taught. Education is what is really needed. Women will always attend if they are invited to come and be taught nicely on this disease and screening. If they understand it well and have all their questions answered, they will want to be screened.
Interviewer: Could there be cultural influences which hinder women to screen?
Participant: In this community no, we don't have such issues. And we don't have those churches which deny people to go for medical treatment, no.
Interviewer: You talked about the problem of distance and suggested that screening should be done at the local health center and you also mentioned that education of women should be done by people coming from Gwanda, could there be something else which could help more women to access screening services in addition to what you have discussed?
Participant: It's what I have said that people need to be educated and the programme should be brought closer to the people. Screening should be done at our clinic because for now people have to travel to Gwanda for screening and most cannot afford the costs.
Interviewer: What other recommendations would you give to make it easier for more women to access screening?
Participant: It is also important to involve the community in these issues so that the programme will be more acceptable. Find out their concerns and ask them how best the service can be delivered considering their beliefs. They may even have some solutions which would permanently solve the problem. Do not decide for them, at times they can have better solutions for themselves.
Interviewer: We have discussed a lot of things. Is there something else I did not ask which you think is useful for effective delivery of the screening service?
Participant: The problem is that *the information comes as highlights and women remain with many questions which are not answered.* We need well informed people to come down and educate the women so that they have full knowledge of the subject covering all aspects of cervical cancer. *What is needed is full information to the people. Also, us Community Health Workers need to be armed with full information, not highlights.* And if possible, screening should be done at local clinics, people will come in their numbers. That is all I can say.
Interviewer: Thank you very much for your time and for all the information you provided. It will be put to good use in the drive to improve delivery of the cervical cancer screening programme in the district.

Interviewer: I also thank you.

1DI 22: VILLAGE COMMUNITY WORKER: WEST NICHOLSON (MINING COMMUNITY)

Interviewer: Welcome to this interview and thank you for your willingness to participate in this study which seeks to find out what could be hindering women from accessing cervical cancer screening services. Could you please explain to me how you are involved in the cervical cancer screening programme.

Participant: My role is to inform women of the dates when the screening outreach team will be visiting our facility, and to sensitise them on screening. I also encourage them to be screened so that they know the status of their cervix.

Interviewer: How long have you been working as a Community Health Worker?

Participant: This is my third year.

Interviewer: Have you received any training pertaining to cervical cancer and screening?

Participant: I have not received specific training pertaining to the screening programme but when we are at the clinic, nurses educate women here and there about cervical cancer and I also acquire the information because I will be listening.

Interviewer: From the knowledge of your community, how common do you think cervical cancer is in your area?

Participant: I think the cases of cervical cancer are there among women. I have heard from those I stay among saying they were screened and told they have cancer. I have heard about 3 people saying that.

Interviewer: In your opinion, how knowledgeable is the community on cervical cancer, and what are their perceptions about the disease and the screening programme?

Participant: Others understand well, taking into consideration the number which has been screened after hearing about screening. Others are those who only go when they are not feeling well or when they are now very sick, and they already have the cancer. But the majority of women especially the young ones, they go although most women have not been screened. They seem to understand about this thing.

Interviewer: Which places are you aware of that offer screening services in the district?

Participant: Women can be screened at Gwanda Provincial Hospital and also here when the outreach team comes.

Interviewer: How often does the outreach team come here?
Participant: If I am not mistaken, maybe twice or three times a year.
Interviewer: Do you have written guidelines which direct you when giving women information on when they should start screening and how frequently they should be screened among other things.
Participant: Mmhh, no we do not have them but there is a book I have. But at one time we were taught when we had attended another workshop although it was not specifically on cervical cancer. They said every year, a woman should be screened.
Interviewer: You mentioned that your job entails educating women on cervical cancer and screening, and also sensitising women for screening when the outreach team will be coming to your area,
Participant: Yes
Interviewer: Please explain on the methods you use to reach out to the women
Participant: There are many ways. When we are weighing babies, we give information. Mothers know that on weighing days we also teach other health related things. So, if that day I decide to talk about cervical cancer, I take along my book which talks about cancer and I talk about it.
Interviewer: From what I understand, you have a book on cervical cancer, what kind of book is that?
Participant: Yes, I have, in fact I can say when I went to that training where I said they touched a bit about cervical cancer, I asked for that book from the District Nursing Offices and I was given. It's because if you don't have full information on something you don't have much to tell people so that's why I asked for that book. So now it is helping me because if I am going to teach the women about cervical cancer, I read first then take my book along to refer to. It has the signs and symptoms of cervical cancer.
Interviewer: How frequently do you meet with the women for these information giving sessions?
Participant: I meet with the women every week. Every week I weigh babies so that's when I meet with them.
Interviewer: In your own opinion, why do you think women are reluctant to go for screening? What could be their reasons?
Participant: Women want to be screened but the challenge we have is that when the outreach team is coming, the clinic delays giving us the date to tell the mothers when there is still time. We usually are told when the team is coming the next day. So, when we go round telling women that there will be screening tomorrow, most would already have made other arrangements and then miss that opportunity. We should be told in time, maybe 5 days before so that we are able to mobilise the women on time. Others say they are afraid. If they are found to have that cancer it will give them stress. Others are not comfortable undressing for other people and exposing their private parts. They say its uncomfortable enough when they give birth and then now again.
Interviewer: Anything else?
Participant: What I have also noticed is that women do not like to be examined by males. For some treatments women are referred to male doctors and they are not comfortable with that. Plus, other nurses are not friendly so women will spread the word and next time the team comes other women won't go. They tell others that they were not treated well. There are also others who when we tell them about screening tell you point blank that I am not coming. Especially those of the Apostolic church. They say the rules of their church do not allow them to go to hospital. There are still some who are like that. Others also fail to properly explain the programme to their husbands then the husband refuses for her to be screened because they will not be understanding what is exactly happening and why his wife should be screened.
Interviewer: You have touched on a lot of things which prevent women from screening. What then do you think should be done to overcome these barriers?
Participant: I think education of women about the programme should be strengthened. For some it's because they have little knowledge and its worse when they have never seen someone suffering from cervical cancer, they won't understand the need to be screened because they don't think it can also happen to her.

Interviewer: Anything else?
Participant: What could also help is to have the outreach team coming more frequently because even if women are willing to be screened, telling them about Gwanda is another story because they say they have no money for bus fare and when I get there, they will want more monies. They don't understand even if you tell them screening is free. It's also because if you have to be treated, you need to pay for that. So, I think it would be better if the screening team could come each and every time, more women would be screened. Unfortunately, when they come, they want to screen only those women who are HIV positive so they should let every women who wants to be screened get screened.
Interviewer: Could there be anything else you want to add which we haven't talked about?
Participant: No. Just a request that we get T-shirts or hats, or something written cervical cancer so that when we talk to mothers, they will take us seriously.
Interviewer: I hear you. Please take your request to the clinic so that they can channel it to the relevant office and thank for your participation. Please continue the good work you are doing.
Participant: Thank you.

IDI 23: VILLAGE COMMUNITY WORKER: MAPHULA WARD

Interviewer: Thank you for allowing me to interview you for this study where I am trying to get information from different categories of health workers on the cervical cancer screening programme in the district.
Participant: Oh
Interviewer: In what capacity are you involved in the cervical cancer programme?

Participant: I am a Community Health Worker and I mobilise women and advise them to be screened. I also mobilise 10 – 14-year-old girls for HPV vaccination to prevent cancer of the cervix. So, when the time for HPV vaccination comes, I go round reminding them and also explaining to the parents what it does and that they should allow their children to be vaccinated. I also keep a file of who has been screened and who tested positive. Those who test positive I encourage them to go to Gwanda for treatment. The challenge is the money to travel to Gwanda.
Interviewer: For how long have you been doing this work?
Participant: I have been a Community Health Worker for 19 years now.
Interviewer: Have you received any training on cervical cancer and screening?
Participant: No, I haven't received any such training.
Interviewer: In your opinion, how prevalent is cervical cancer in your community?
Participant: I would say for now it's still very low. I only know of 5 women who were screened and told to Gwanda for treatment. But then I don't know because for now only those who are HIV positive are the ones who are screened. Only a few who are HIV negative are screened. We also have many women who have reported that they have some lumps in their breasts. If it were possible, it is better to screen everyone including those who are HIV negative and even screen for breast cancer.
Interviewer: Would you know the reason why only HIV positive women are screened?
Participant: They are saying those who are HIV positive have a higher risk for cervical cancer.
Interviewer: From your assessment, what is the level of knowledge and perceptions of the community on cervical cancer?
Participant: They say cervical cancer kills, so they wish to be screened so that they can get treatment if it is found early. This is because we have had a woman who died from that disease the other year. At that time, they were taking the disease lightly but now they know that cancer kills. Everyone is now afraid of it and they want to be screened. So, I would say the women have the knowledge.
Interviewer: Ok, and how aware is the community about cervical cancer screening?
Participant: They know about it and they wish to be screened. I say so because when the clinic gives us a date of when the mobile clinic is coming, I go round mobilising the HIV positive women. The HIV negative also complain because they want to be screened. That is the challenge I have. And also, they want those aged 25 -50 years if I am not mistaken. The rest are not taken.
Interviewer: Where will the mobile team be coming from?
Participant: There I am not sure, but it should be the Ministry of Health I think because it's the clinic which tells us to mobilise. They come at times. May be after 6 months or so.
Interviewer: Which places in the district do you know that offer screening services?
Participant: I hear they screen at Gwanda Provincial Hospital and they also come to the clinic at times where I said here, they only screen those who are HIV positive.
Interviewer: Do you have written guidelines on screening protocols, that is when women should start screening, how often they should be screened, when they can stop screening and such other information?
Participant: We have been told that HIV positive women should be screened every year and those who are negative every 2 years. We are just told at the clinic, but we don't have any written thing to refer to such as pamphlets.
Interviewer: You mentioned that you do a lot of mobilising of women for screening and you also give them information on cervical cancer. So, where do you meet the women?
Participant: I talk to them when they bring their babies for weighing and also at community meetings and Health Clubs. I can say we include health matters in everything where people are gathered. But especially I meet the mothers at the weighing points.
Interviewer: Please give more information about the health clubs.
Participant: These are clubs where we teach mothers about cleanliness in their homes, about a model home which should have a toilet, pits, and pot racks. We teach them how to use mosquito

nets in a proper way and we also give lessons on malaria, TB, skin and other diseases. But as for cancer we haven't talked about it in the health clubs.
Interviewer: So how often do you meet mothers at the weighing points?
Participant: We meet every month.
Interviewer: In your own view, what could be the reasons which hinder some women from accessing screening services?
Participant: The major reason why few women have been screened than they should be is distance because screening is done in Gwanda. Even when they come down here, they should not choose, they should screen everyone who wants screening. The problem is that even if they come to screen here, those found with problems are told to come to Gwanda for treatment then they don't go because they have no money. So otherwise women don't see the need of screening since they will still remain with their problems. The other problem lies with the women, they are afraid of what others will say if she is screened and found with cancer. Then when it comes to churches, we have the Masowe church which does not allow their members to be treated in hospitals. They say they are able to treat cancer. Those are individuals who say that, but I asked their leaders and they said no, it's not the church which says that.
Interviewer: How common is it for people to consult prophets and traditional healers for treatment?
Participant: Mmhh, except for members of those churches, most people are now are religious and don't go to traditional healers. Most now go to the clinics because they have realised that many people die, so it's better to go to the hospital.
Interviewer: Are there any other barriers to screening you can think of?
Participant: Mmhh, no
Interviewer: Ok, so in your opinion, how do you think these barriers can be overcome, apart from what you have already discussed?
Participant: First, the doctors should also come with the Mobile team to treat those women who are found with a problem during screening. As it is, I have seen 5 women who were screened about 6 months ago and were told to come to Gwanda for treatment. They still have not gone because they have no money and the disease is progressing. So, when they tell other women who have not been screened, those ones will be discouraged because it means it's useless to be screened. I would also recommend that they scrape the age group and HIV status as their criteria for screening and screen everybody. Also, they should come more frequently not after such a long time. They can allow us to mobilise women who want to be screened, then when we have a good number, we tell them so that they plan a date to come instead of staying such a long time without coming.
Interviewer: Would there be anything else you would want to add?
Participant: There should be a system where if a woman tests positive on screening, the clinic lets us know so that we make a follow up for them to go to Gwanda. Now we have to ask the women and others are not free to disclose to us if they test positive.
Interviewer: Are you satisfied with the knowledge you have on cervical cancer? Do you view the knowledge you have as adequate enough to effectively educate the women in your community?
Participant: The issue is that I have never received any training or attended any workshop specific to VIAC, so I still need more information. But I read pamphlets on VIAC so that I get the necessary information to tell the mothers. The nurses also inform us about the disease, but I have not had any training on cervical cancer and its prevention. Posters and pamphlets are also not readily available to share with the mothers so I would suggest that we are given these materials to give the mothers to read. It would make it easier even for us to educate them because they will ask what they failed to understand from what they would have already read.
Interviewer: Is there anything else you would want to comment on?
Participant: Mmhh, no.
Interviewer: I would like to thank you for you participation and the information you have given me will be put to good use.

**Participant**: You are welcome.
**IDI 24: VILLAGE COMMUNITY WORKER: GARANYEMBA WARD**
**Interviewer:** Thank you for agreeing to participate in this study which aims at determining the factors that contribute to low uptake of cervical cancer screening programme by women in Gwanda district. May you please share with me how you are involved in this programme.<br>**Participant**: This is a very important programme and very helpful in the district. I talk to people about the programme, and they appreciate it. Then they have the problem and say to me "even if we wish to be screened, our problem is that it's too far, it requires money". So, the problem is bus fare to Gwanda where screening is done. But when I talk to them, they really understand about the programme. Because this programme even in the radios they talk about it, it's something which they hear about all the time. They say yes screening is free, but the problem is transport. We have no money. So many wish but have not been screened.<br>**Interviewer:** I understand. Could you kindly elaborate more on what role you play in the programme?<br>**Participant**: I tell people about the programme and encourage women to go for screening.<br>**Interviewer:** How long have you been serving this community as a Community Health Worker?<br>**Participant:** I was trained in 2010, so I have been in this job for 10 years now.<br>**Interviewer:** Concerning cervical cancer screening, have you received training specific to the programme?<br>**Participant**: The training pertaining to screening for cervical cancer I have not received. But when we attend other workshops and meetings at the clinic, we are told about it. No, I have not received VIAC training. I would be very happy to receive such training so that I get the full information to teach people. I need more information on that.<br>**Interviewer**: From your assessment, how prevalent do you think cervical cancer is in your catchment area?<br>**Participant:** Cervical cancer is there because we have had some women who had this cancer, and some of them died, and some went to hospital and are still receiving treatment. Others have told me that they were told they had cervical cancer when they were screened and were treated. And looking at them you can tell they are well. Not knowing whether it will resurface later.<br>**Interviewer:** According to your own assessment, how knowledgeable is the community on cervical cancer and what are their perceptions about the disease?<br>**Participant:** The community has got the knowledge. But the fact is that traditionally people still associate cancer with witchcraft and *some are still holding back.* But if you teach them, they end up understanding that cervical cancer is a disease just like any other which can be treated in hospital. Especially one can get it through having sex with many men. They are the ones who bring it to us because if he sleeps with one who has it and then comes to you, you can get it. Yes, it can be witchcraft but it's a disease which can be treated by doctors. What is important is for people to get screened all the time so that it can be picked early, and the person treated.<br>**Interviewer:** Regards the VIAC screening programme, how aware are the people about it in your community?<br>**Participant**: People know about this programme because it's talked about everywhere and now it has even spilled to young girls. People know because we go round to households sensitising parents

about the school-based programme for girls and finding those who are not going to school to go to the clinic. These are girls, age 10-14 years who have to be given the HPV vaccine. So, we discuss with the families about both cancer of the cervix and screening and the importance of HPV vaccination. This is how I can tell that people now understand about the screening programme. Even when there are gatherings, we always ask for time from the owners of the meeting to talk about screening and HPV vaccination of 10-14-year aged girls to prevent cervical cancer.

Interviewer: What kind of gatherings do you take advantage of?

Participant: We use community meetings where we ask for slots to talk about the screening programme. Always they will cry about transport money and ask why they cannot be screened here more often where we won't have to incur transport costs. So, if those screening could come to the clinic it would be easier for women from all parts of the ward although some are still far from the clinic, but it's better than going to Gwanda.

Interviewer: As the Community Health Worker who mobilises and educates women on screening, do you have the Ministry of Health guidelines on the class of women who should be screened and how often they should be screened?

Participant: I do not have any written thing to guide me in my education of women, but I use the information I get from the nurses.

Interviewer: In your view, apart from what you have already mentioned such as distance, what could be the other reasons which make women reluctant to go for screening?

Participant: Yes, some women are scared that if they go for screening, they will be found to be having cervical cancer. This will give them stress when they think how they will have the disease treated since they don't have money for treatment. That's one of the main reasons which make women not screen. What they need is more education to say even if they are found to have cancer, it may still be treated. Women believe that once you have cancer nothing can be done, and you will die.

Interviewer: Is there a system in place for follow up of the women who test positive on VIAC screening so that you keep track that they have gone to Gwanda for treatment?

Participant: Sometimes the women will tell me their results, but usually I have no way of knowing unless if the nurses tell us. But most of the time they do.

Interviewer: Ok, you have mentioned some barriers which prevent women from screening. So, in your view, how can these challenges be addressed? I mean on those which you have not already discussed because you have given your proposed solutions on some?

Participant: My major recommendation is to have cervical cancer screening done at the rural health centre as part of the services. Even if it's not every day, it's easier than having to go to Gwanda. Once the mothers get to know the screening days, many women will come. Because many women now understand the importance of screening although there are some who are afraid. Like I said many are willing to be screened. Then for those who believe in traditional methods of attending to cervical cancer, education should be continued. They can even be told to start at the clinics for screening and treatment if they need to be treated then resort to their traditional methods if it does not respond. That way they will feel their beliefs are also recognised and this might kill the resistance. Some diseases are treated traditionally but the problem is that there is no cleanliness which may make the illness worse. So, we need to have ways of removing them from the beliefs in traditional treatments to medical treatment. If done well, they may be convinced. There are many who have left that through our engagements with them. These people are known from our interaction with them, but they are very few. This is because many people now understand that cancer of the cervix can kill. We have had some deaths from that disease in this community, and those were the ones who were getting treatment from traditional healers. From those deaths, people now understand these health issues.

Interviewer: Ok. So, from all that we have discussed today on the barriers to cervical cancer screening and how these can be overcome, is there anything else we did not touch on which you would like to add?
Participant: My recommendation is that we have cervical cancer screening done at this clinic. As I have said, many women wish to be screened but the problem is that they have to go to Gwanda to get that service and then they tell you they have no money. So that is our request. And another request is that as Community Health Workers, can we get full training on cervical cancer and the screening programme so that we are able to give people full information.
Interviewer: Thank you very much. I heard your views and hope the study findings will contribute towards addressing some of these challenges.
Participant: I also thank you.

IDI 25: VILLAGE COMMUNITY WORKER: STANMORE WARD

Interviewer: I would like to thank you for your willingness to be part of this study which seeks to determine the barriers to cervical cancer screening in the district**.**
Participant: It's my pleasure.
Interviewer: In your work as a Community Health Worker, could you kindly tell me how you are involved in the cervical cancer screening programme?
Participant: Ok, What I do is have a lot of discussions with women informing them of the programme and the importance of screening for cervical cancer. I do that many times. Telling them that it is important to screen so that they know the state of their cervix. I always tell them that it is not right to delay screening until they are in pain.
Interviewer: How long have you been a Community Health Worker?
Participant: It's now 3 years
Interviewer: Since you give a lot of education on cervical cancer and screening, have you received some training to empower you with information for effective delivery of this information to women?
Participant: We always go for different training as VCWs, but we have not had one particularly for cervical cancer although it is mentioned in some trainings we have had.
Interviewer: With the knowledge of your community, how common do you think cervical cancer is in this area?
Participant: It's not very common, I only know of 2 women one of whom ended up going to South Africa for treatment.
Interviewer: How much knowledge do you think the community has on cervical cancer and how do they view the disease?
Participant: They have the knowledge that there is such a disease. Only that women are reluctant to stand up and go for screening until there is a problem because there is no screening at the clinic. But when the screening team comes to the clinic, and they are informed in time, they really go. But unfortunately, those who come to screen only want those on ART and of a certain age group, can't remember exactly. This closes other women out. Women say that it would be easier for them to be

screened at the clinic where they won't need to find money for transport, but they are closed out. Our request is that if they could screen all women of all ages and include even those not on ART because all women are at risk of the disease and want to be screened.
Interviewer: Where are cervical cancer screening services provided in Gwanda district?
Participant: There is an outreach team which comes to the clinic to screen women once in a while.
Interviewer: Are there other places in the district you could refer women for screening. Where else are screening services providers?
Participant: Except for the outreach team, screening is done at Gwanda Provincial Hospital. We encourage women to go there.
Interviewer: You said an outreach team comes to screen women……
Participant: Yes, but they only screen those on ART and a certain age group.
Interviewer: How often does the outreach team come to the clinic?
Participant: It's once a year, they don't come all the time. This year they have not come yet.
Interviewer: In your community, how would you rate the uptake of screening?
Participant: The acceptance of the screening programme is very good, and women are willing to be screened but few women have been screened. The problem is the discrimination on screening where there is a specific group of people who are wanted. Others who are HIV negative still go and some have been turned away without being screened. So, many of those on ART have been screened. Some HIV negative women have also been screened because they still go and after the team is done with the HIV positive ones they may be screened. But others would already have left after being told the team is only screening HIV positive women only.
Interviewer: Where do you get the information which guides you on when women should start screening and how frequently they should be screened to assist you in educating the women about screening? I am asking this because you said you have not been trained on VIAC?
Participant: The nurses inform us that a woman should be screened every year, so if they are screened this year, they should wait till next year to be screened again when they have completed a year.
Interviewer: You talked about sensitising women, giving them information on screening and encouraging them to be screened. So, which methods do you use to reach women with information?
Participant: We have women support groups which we use to disseminate information to women on health issues. We also teach them when they bring their babies for weighing. We also have village WhatsApp groups. I also put information there to reach those with no babies who cannot access information from the weighing point. I also talk about the programme when I do home visits.
Interviewer: How often do you access mothers when they bring their children for weighing?
Participant: This weighing is not done at the clinic. They come to me to have their babies weighed once a week. They only go to the clinic if they are due for immunisations.
Interviewer: Ok.
Participant: Yes, we also give Vitamin A, they don't have to go to the clinic for that. I have a day every week where mothers bring the children for weighing. We also meet the support groups every week. For, the support groups, these are clubs where women meet to discuss income generation projects and money clubs. So, I also meet them there and give information on screening.
Interviewer: The women who have been screened are far below the number we expect. What could be the problems which women face in accessing screening services?
Participant: For most, the challenge is on transport to go to Gwanda. They don't have the money. The other problem is that when the Gwanda people come to screen here at the clinic, they don't screen every woman. They choose who they want to screen. We ask that when they come next time to screen all women who are interested and not only those on ART.
Interviewer: Could there be any other challenges?

Participant: The other thing is that women don't want to expose their private parts (laughs). Honestly, they are not comfortable with that. That's why they are even reluctant to use the loop for contraception. They don't want to open their private parts. If screening was being done using a different method which does not involve private parts, maybe the screening uptake could increase.
Interviewer: How supportive are the men on women to get screened?
Participant: Most men are not bothered one way or another, but I don't think they refuse for wives to be screened. There are a few though who when you put a message on the village WhatsApp group, they will even comment, meaning that if men were to be fully involved in this programme, we could see more women coming up for screening.
Interviewer: You said women had some knowledge on screening. Can you elaborate further on their level of understanding and differentiate it from awareness about the programme?
Participant: It could be that they don't have full understanding because even us need full training on the programme and refresher courses so that we are able to give full information to the women. The VIAC team should also at times come, just to teach the women. It would help for them to get the information from people with better knowledge different from us. It's nice to have different people coming to talk about the same thing who are not part of the community. The community will realise that the programme is more important than they think. Some community members look down on us.
Interviewer: You have mentioned different challenges to screening and even highlighted the solutions to some of the issues. How can the other barriers be overcome?
Participant: The outreach programme should be more frequent in coming because women don't have money to go to Gwanda. And, when they come, they should screen all women who are interested without putting barriers of HIV status and age.
Interviewer: What other recommendations would you suggest for improving access to screening?
Participant: I am thinking that the best thing is to train our local nurses so that screening can be done at the clinic at any time not this where the service is selective and comes once after a long time.
Interviewer: Have you at a personal level encountered any challenges when educating women about cervical cancer?
Participant: Yes, at times women ask questions which I cannot answer. For example, they will ask you; have you seen someone with cancer of the cervix for you to tell us what the signs look like? I think it would be more effective to have people who talk from experience coming to talk to them at times, that way they will understand better. As long as they have not seen or known someone who has cervical cancer, they will doubt and think it's not common and they are not likely to get it.
Interviewer: Do you have anything else you would like to add which we did not talk about?
Participant: Other people believe that once you get screened, you are inviting problems because your cervix will have been touched therefore you will always have cervix problems for life. So, it's better not to be screened. An outside person coming to talk to women once in a while would help to keep them motivated.
Interviewer: Thank you so much for your participation. Your contributions will be combined with those of others who participated in the study to make recommendations to the district local authorities which could help to improve the screening services.
Participant: Thank you.

KEY INFORMANTS (ADMINISTRATORS)				
**1**	**2**	**3**	**4**	**5**
**Role in VIAC programme**<br>✓ Programme overseer<br>✓ Mentor	**Role in VIAC programme**<br>✓ Perform LEEP and ablation<br>✓ Coordinate quality control meetings	**Role in VIAC programme**<br>✓ Ensure smooth running of the programme<br>✓ Ensure staff adherence to screening guidelines<br>✓ Ensuring availability of equipment<br>✓ M & E of the programme	**Role in VIAC programme**<br>✓ Information dissemination on CC and screening<br>✓ Sensitise men on VIAC programme<br>✓ Distribute IEC materials	**Role in VIAC programme**<br>✓ Not hands on<br>✓ Health education to women on screening
**Period in current role**	**Period in current role**	**Period in current role**	**Period in current role**	**Period in current role**
		✓ 5 years	✓ 4 months	✓ 8 months
**Trained in VIAC?**	**Trained in VIAC?**	**Trained in VIAC?**	**Trained in VIAC?**	**Trained in VIAC?**
✓ By default	✓ Skilled in VIAC treatment	✓ Not VIAC trained	✓ Not VIAC trained	✓ Not VIAC trained
**CC prevalence in catchment area**	**CC prevalence in catchment area**	**CC prevalence in catchment area**	**CC prevalence in catchment area**	**CC prevalence in catchment area**
✓ High burden of CC & precancerous conditions	✓ No information on the prevalence of CC	✓ High burden of precancerous lesions	✓ High burden of CC	✓ High burden of CC
**Women's knowledge on CC**	**Women's knowledge on CC**	**Women's knowledge on CC**	**Women's knowledge on CC**	**Women's knowledge on CC**
✓ Lack adequate knowledge on CC	✓ Lack adequate knowledge on CC		✓ Lack adequate knowledge on CC	✓ Lack adequate knowledge on CC
**Women's knowledge on screening**	**Women's knowledge on screening**	**Women's knowledge on screening**	**Women's knowledge on screening**	**Women's knowledge on screening**
✓ Lack awareness about screening<br>✓ Lack adequate knowledge on VIAC	✓ Lack awareness about screening<br>✓ Lack adequate knowledge on VIAC	✓ Women aware of the VIAC programme	✓ Lack adequate knowledge on VIAC	✓ Lack adequate knowledge on VIAC
**Available CC service providers**	**Available CC service providers**	**Available CC service providers**	**Available CC service providers**	**Available CC service providers**
✓ GPH<br>✓ Phakama Urban Clinic	✓ GPH<br>✓ Phakama Urban Clinic	✓ GPH<br>✓ Phakama Clinic<br>✓ Outreach services	✓ GPH<br>✓ Phakama Clinic<br>✓ Outreach services	✓ GPH<br>✓ Phakama Clinic<br>✓ Outreach services

**Prevalence of screening**	**Prevalence of screening**	**Prevalence of screening**	**Prevalence of screening**	**Prevalence of screening**
✓ Uptake of screening low		✓ Uptake of screening low	✓ Uptake of screening low (30%)	✓ Uptake of screening is low
**Awareness of CC prevention & control guidelines**	**Awareness of CC prevention & control guidelines**	**Awareness of CC prevention & control guidelines**	**Awareness of CC prevention & control guidelines**	**Awareness of CC prevention & control guidelines**
✓ Aware of ZCCPCS guidelines	✓ Aware of ZCCPCS guidelines	✓ Aware of ZCCPCS guidelines<br>✓ Easy to implement	✓ Aware of ZCCPCS guidelines	✓ Not aware of ZCCPCS guidelines
**Demand creation strategies**	**Demand creation strategies**	**Demand creation strategies**	**Demand creation strategies**	**Demand creation strategies**
	✓ Coordinated by nurses	✓ Second VIAC trained nurse to district integrated outreach team<br>✓ Municipality VIAC awareness programme<br>✓ VIAC information given at the OIC<br>✓ VIAC outreach services where information on VIAC is also disseminated	✓ Primary health facilities give education on screening	✓ VIAC awareness talks in ward

Barriers to CC screening	Barriers to CC screening	Barriers to CC screening	Barriers to CC screening	Barriers to CC screening
✓ Lack of adequate equipment<br>✓ Inadequate screening sites<br>✓ Inaccessibility of screening services to rural communities<br>✓ Poor health seeking behaviours<br>✓ Women lack awareness about VIAC<br>✓ Women lack adequate knowledge on VIAC<br>✓ Women lack of adequate knowledge about CC<br>✓ Shortage of VIAC trained nurses<br>✓ Gap in outreach service provision because of partner's requisites<br>✓ Inadequate demand creation strategies<br>✓ LEEP specimens tested at higher level laboratories<br>✓ VIAC programme underfinanced<br>✓ COVID-19 restrictions	✓ Lack of adequate equipment<br>✓ Inadequate screening sites<br>✓ Inaccessibility of screening services to rural communities<br>✓ Poor health seeking behaviours<br>✓ Women lack awareness about VIAC<br>✓ Women lack adequate knowledge on VIAC<br>✓ Women lack adequate knowledge about CC<br>✓ Shortage of VIAC trained nurses<br>✓ VIAC nurses multitasking<br>✓ VIAC nurses lack motivation<br>✓ Fear of a cancer diagnosis<br>✓ LEEP specimens tested at higher level laboratories<br>✓ VIAC programme underfinanced	✓ Lack of adequate equipment<br>✓ Power outages<br>✓ Lack of consistency in outreach services<br>✓ Financial constraints: Lack of money for transport<br>✓ Loss to follow up<br>✓ Low socio-economic status<br>✓ Shortage of VIAC trained nurses<br>✓ VIAC nurses multitasking<br>✓ VIAC clinic closes weekends & public holidays<br>✓ Socio-cultural beliefs<br>✓ Inaccessibility of screening services to rural communities<br>✓ VIAC programme underfinanced<br>✓ LEEP specimens tested at higher level laboratories<br>✓ Religious beliefs	✓ Lack of adequate equipment<br>✓ Financial constraints: Lack of money for transport and treatment<br>✓ Shortage of VIAC trained nurses<br>✓ Women lack of adequate knowledge about CC<br>✓ Women lack adequate knowledge on VIAC<br>✓ Negative attitudes towards screening<br>✓ Lack of adequate transport for outreach services<br>✓ Socio-cultural beliefs<br>✓ Mobility of target population<br>✓ Lack of consistency in outreach services<br>✓ Loss to follow up<br>✓VIAC programme underfinanced<br>✓COVID-19 restrictions	✓ Shortage of VIAC trained nurses<br>✓ Socio-cultural beliefs<br>✓ Nurses lack adequate information on CC and VIAC<br>✓ Women lack of adequate knowledge on CC<br>✓ Women lack adequate information on VIAC<br>✓ Financial constraints: Lack of money for transport<br>✓ Inadequate screening sites<br>✓ VIAC programme under financed

Strategies to address barriers	Strategies to address barriers	Strategies to address barriers	Strategies to address barriers	Strategies to address barriers
✓ All health facilities to provide screening services<br>✓ Train more nurses on VIAC<br>✓ Health provider initiated screening<br>✓ Involvement of Community Health Nurses in awareness campaigns<br>✓ Involvement of Community Health Workers in awareness campaigns<br>✓ Implement low cost high impact initiatives combined with HPV vaccination<br>✓ Screen every woman<br>✓ Allocate adequate funding for the VIAC programme	✓ All health facilities to provide screening services<br>✓ Regular training of nurses doing VIAC<br>✓ Have staff dedicated to VIAC<br>✓ Continued education of women<br>✓ Provide adequate equipment<br>✓ Provide incentives for VIAC staff<br>✓ Address poverty issues<br>✓ Allocate adequate funding for the VIAC programme	✓ Increase outreach services<br>✓ Allocate adequate funding for the VIAC programme<br>✓ All health facilities to provide screening services<br>✓ Upgrade GPH to do histology tests<br>✓ Train more nurses on VIAC<br>✓ Open more screening sites<br>✓ Find strategies to break religious barriers	✓ Male involvement<br>✓ Increase awareness creation strategies on CC and screening<br>✓ Increase outreach services<br>✓ Find innovative ways of educating women about CC<br>✓ Educate communities first to lay ground for screening<br>✓ Provide adequate equipment<br>✓ Provide full free package for VIAC<br>✓ Provide treatment at outreach sites<br>✓ Provide IEC materials in the local language	✓ Increase awareness creation strategies on CC and screening<br>✓ Allocate adequate funding for the VIAC programme<br>✓ All health facilities to provide screening services<br>✓ Train all nurses on VIAC screening<br>✓ Use innovative strategies for awareness creation<br>✓ Involve influential community leaders as important stakeholders in the VIAC programme

KEY INFORMANTS (VIAC TRAINED NURSES)				
**6**	**7**	**8**		
**Role in VIAC programme**<br>✓ Provide screening services<br>✓ Provide ANC services	**Role in VIAC programme**<br>✓ Provide screening services	**Role in VIAC programme**<br>✓ Sensitise women for screening<br>✓ Educate women on CC and screening<br>✓ Provide screening services	**Role in VIAC programme**	**Role in VIAC programme**
**Period in current role**	**Period in current role**	**Period in current role**	**Period in current role**	**Period in current role**
4 years	2 years	4 years		
**Trained in VIAC?**	**Trained in VIAC?**	**Trained in VIAC?**	**Trained in VIAC?**	**Trained in VIAC?**
VIAC trained	VIAC trained	VIAC trained		
**CC prevalence in catchment area**	**CC prevalence in catchment area**	**CC prevalence in catchment area**	**CC prevalence in catchment area**	**CC prevalence in catchment area**
✓ High burden of precancerous conditions	✓ High burden of CC	High burden of CC		
**Women's knowledge on CC**	**Women's knowledge on CC**	**Women's knowledge on CC**	**Women's knowledge on CC**	**Women's knowledge on CC**
✓ Lack adequate knowledge about CC	✓ Lack adequate knowledge about CC	✓ Lack adequate knowledge about CC		
**Women's knowledge on screening**	**Women's knowledge on screening**	**Women's knowledge on screening**	**Women's knowledge on screening**	**Women's knowledge on screening**
✓ Lack adequate knowledge on VIAC	✓ Lack awareness on VIAC<br>✓ Lack adequate knowledge on VIAC	✓ Lack adequate knowledge on VIAC		
**Available CC service providers**	**Available CC service providers**	**Available CC service providers**	**Available CC service providers**	**Available CC service providers**
✓ GPH<br>✓ Phakama clinic<br>✓ Outreach services	✓ GPH<br>✓ Outreach services	✓ GPH<br>✓ Phakama clinic<br>✓ Outreach services<br>✓		
**Prevalence of screening**	**Prevalence of screening**	**Prevalence of screening**	**Prevalence of screening**	**Prevalence of screening**

✓ Uptake of screening is low	✓ Uptake of screening is low	✓ Uptake of screening low		
**Awareness of CC prevention & control guidelines**	**Awareness of CC prevention & control guidelines**	**Awareness of CC prevention & control guidelines**	**Awareness of CC prevention & control guidelines**	**Awareness of CC prevention & control guidelines**
✓ Aware of ZCCPCS guidelines	✓ Aware of ZCCPCS guidelines	✓ Aware of ZCCPCS guidelines<br>✓ Aware of OPHID guidelines		
**Demand creation strategies**	**Demand creation strategies**	**Demand creation strategies**	**Demand creation strategies**	**Demand creation strategies**
✓Health education given in other departments<br>✓Mobilisation by primary health care nurses	✓ Outreach clinics	✓ Target women at entry points<br>✓ Give information on VIAC to admitted patients		
**Barriers to CC screening**	**Barriers to CC screening**	**Barriers to CC screening**	**Barriers to CC screening**	**Barriers to CC screening**
✓ Inaccessibility of screening services to rural communities<br>✓ Inadequate screening sites<br>✓ Gap in service provision because of partner's requisites<br>✓ Lack of adequate transport for outreach services<br>✓ Women lack adequate knowledge on VIAC<br>✓ Women lack adequate knowledge about CC<br>✓ Religious beliefs<br>✓ Shortage of VIAC trained nurses<br>✓ Lack of partner support	✓ Women lack adequate knowledge about CC<br>✓ Women lack awareness on VIAC<br>✓ Women lack adequate knowledge on VIAC<br>✓ Inaccessibility of treatment services for rural communities<br>✓ Financial constraints: lack of money for transport and treatment<br>✓ Lack of consistency in outreach services<br>✓ Gap in service provision because of partner's requisites<br>✓ Fear of stigma and discrimination	✓ Women lack adequate knowledge about CC<br>✓ Women lack awareness on VIAC<br>✓ Women lack adequate knowledge on VIAC<br>✓ Negative attitudes towards screening<br>✓ Low risk perception<br>✓ Inadequate demand creation strategies<br>✓ Fear of a cancer diagnosis<br>✓ Fear of procedure<br>✓ Nurses lack confidentiality<br>✓ Gap in service provision because of implementing partner's requisites		

✓ VIAC clinic close weekends and public holidays<br>✓ Long waiting periods<br>✓ Lack of adequate equipment<br>✓ Poor infrastructure<br>✓ COVID-10 restrictions	✓ Inadequate demand creation strategies<br>✓ Fear of procedure<br>✓ Shortage of VIAC trained nurses<br>✓ Religious beliefs<br>✓ Apathy	✓ Negative staff attitudes<br>✓ VIAC nurses multitasking: give inadequate information to women<br>✓ Inaccessibility of screening services to rural communities<br>✓ Financial constraints: Lack of money for transport<br>✓ Lack of partner support<br>✓ Myths and misconceptions<br>✓ Lack of trust in health services<br>✓ Lack of consistency in outreach services<br>✓ VIAC units close during weekends & public holidays<br>✓ Fear of stigma and discrimination<br>✓ COVID-19 restrictions		
**Strategies to address barriers**	**Strategies to address barriers**	**Strategies to address barriers**	**Strategies to address barriers**	**Strategies to address barriers**
✓ All health facilities to provide screening services<br>✓ Provide a full free package for VIAC<br>✓ Have a dedicated VIAC unit	✓ Intensify education on screening<br>✓ Train more nurses in VIAC<br>✓ All health facilities to provide screening services<br>✓ Increase outreach services<br>✓ Screen all women	✓ Conduct mass education campaigns: Educate communities first to lay ground for screening<br>✓ Increase outreach services<br>✓ Have 2 outreach teams		

✓ Provide adequate equipment	✓ Health provider initiated screening	✓ Roll out VIAC services to high risk groups		

**GWANDA PROVINCIAL HOSPITAL NURSES**				
**9**	**10**	**11**	**12**	**13**
**Role in VIAC programme**<br>✓ Sensitise women for screening	**Role in VIAC programme**<br>✓ Not involved in VIAC programme<br>✓ Refer women to VIAC clinic<br>✓ Sometimes initiate women for screening	**Role in VIAC programme**<br>✓ Initiate women for screening	**Role in VIAC programme**<br>✓ Not directly involved<br>✓ Sensitise women for screening	**Role in VIAC programme**<br>✓ Not highly involved in VIAC programme<br>✓ Sensitise women for screening
**Period in current role**	**Period in current role**	**Period in current role**	**Period in current role**	**Period in current role**
✓ 1 year		✓ 3 years	✓ 2 years	✓ 3 years
**Trained in VIAC?**	**Trained in VIAC?**	**Trained in VIAC?**	**Trained in VIAC?**	**Trained in VIAC?**
✓ Not VIAC trained	✓ Not VIAC trained	✓ Not VIAC trained	✓ Not VIAC trained	✓ Not VIAC trained
**CC prevalence in catchment area**	**CC prevalence in catchment area**	**CC prevalence in catchment area**	**CC prevalence in catchment area**	**CC prevalence in catchment area**
✓ High burden of CC	✓ No idea	✓ High burden of CC	✓ High burden of CC	✓ High burden of CC
**Women's knowledge on CC**	**Women's knowledge on CC**	**Women's knowledge on CC**	**Women's knowledge on CC**	**Women's knowledge on CC**
✓ Have knowledge on CC	✓ Suppose they know	✓ Lack adequate knowledge about CC	✓ Lack adequate knowledge about CC	✓ Lack adequate knowledge about CC
**Women's knowledge on screening**	**Women's knowledge on screening**	**Women's knowledge on screening**	**Women's knowledge on screening**	**Women's knowledge on screening**
✓ Aware of VIAC services	✓ Suppose they know	✓ Lack awareness on VIAC<br>✓ Lack adequate knowledge on VIAC	✓ Lack adequate knowledge on VIAC	✓ Lack adequate knowledge on VIAC
**Available CC service providers**	**Available CC service providers**	**Available CC service providers**	**Available CC service providers**	**Available CC service providers**
✓ GPH: 2 Units<br>✓ Phakama clinic	✓ GPH	✓ GPH	✓ GPH<br>✓ Outreach services	✓ GPH<br>✓ Outreach services

✓ Outreach services				
**Prevalence of screening**	**Prevalence of screening**	**Prevalence of screening**	**Prevalence of screening**	**Prevalence of screening**
✓ Not sure of screening prevalence	✓ No idea	✓ Uptake of screening is low	✓ Uptake of screening is low	✓ Uptake of screening is low
**Awareness of CC prevention & control guidelines**	**Awareness of CC prevention & control guidelines**	**Awareness of CC prevention & control guidelines**	**Awareness of CC prevention & control guidelines**	**Awareness of CC prevention & control guidelines**
✓ Not aware of ZCCPCS guidelines	✓ Not aware of ZCCPCS guidelines	✓ Aware of ZCCPCS guidelines	✓ Not aware of ZCCPCS guidelines	✓ Not aware of ZCCPCS guidelines
**Demand creation strategies**	**Demand creation strategies**	**Demand creation strategies**	**Demand creation strategies**	**Demand creation strategies**
✓ Health education given in the department	✓ Nil	✓ Encourage all ART clients to be screened and disseminate information to relatives	✓ Opportunistic education of women in Ward	✓ Opportunistic education of women in Ward
**Barriers to CC screening**	**Barriers to CC screening**	**Barriers to CC screening**	**Barriers to CC screening**	**Barriers to CC screening**
✓ Low risk perception<br>✓ Shortage of VIAC trained nurses<br>✓ VIAC nurses multitasking<br>✓ Poor infrastructure<br>✓ Fear of stigma and discrimination<br>✓ Religious beliefs<br>✓ Lack of adequate equipment<br>✓ Nurses lack adequate knowledge on CC screening	✓ Women lack awareness about VIAC<br>✓ Nurses lack adequate knowledge about CC and screening<br>✓ Nurses lack motivation<br>✓ Inadequate demand creation strategies	✓ Women lack adequate knowledge about CC<br>✓ Women lack awareness on VIAC<br>✓ Women lack adequate knowledge on VIAC<br>✓ Fear of a cancer diagnosis<br>✓ Poor infrastructure<br>✓ VIAC programme underfinanced<br>✓ Negative peer influence<br>✓ Fear of procedure<br>✓ Financial constraints: lack of money for transport<br>✓ Socio-cultural beliefs<br>✓ Lack of partner support	✓ Nurses lack adequate knowledge about CC and screening<br>✓ Women lack adequate knowledge about CC<br>✓ Women lack adequate knowledge on VIAC<br>✓ Low risk perception<br>✓ Inaccessibility of screening services to rural communities<br>✓ Low socio-economic status<br>✓ Financial constraints: Lack of money for transport<br>✓ Religious beliefs	✓ Women lack adequate knowledge about CC<br>✓ Women lack adequate knowledge about VIAC<br>✓ Nurses lack adequate knowledge about CC and screening<br>✓ Socio-cultural beliefs<br>✓ VIAC programme under financed

			✓ Lack of partner support<br>✓ Inaccessibility of treatment services for rural communities	
**Strategies to address barriers**	**Strategies to address barriers**	**Strategies to address barriers**	**Strategies to address barriers**	**Strategies to address barriers**
✓ Increase awareness creation strategies on CC and screening<br>✓ Male involvement in education about CC<br>✓ Train more nurses in VIAC<br>✓ Have a dedicated VIAC unit<br>✓ Provide VIAC screening at all female departments<br>✓ Provider initiated screening<br>✓ Increase outreach services	✓ Increase awareness creation strategies on CC and screening<br>✓ Distribute IEC materials<br>✓ Increase outreach services	✓ Increase awareness creation strategies on CC and screening<br>✓ Use innovative strategies for awareness creation<br>✓ Provide VIAC screening at the OIC<br>✓ Increase outreach services<br>✓ Educate communities first to lay ground for screening	✓ Increase awareness creation strategies on CC and screening<br>✓ Conduct mass education campaigns<br>✓ Consistency of outreach services<br>✓ Provide transport to GPH for women who test VIAC positive to access treatment	✓ Increase awareness creation strategies on CC and screening<br>✓ Educate communities first to lay ground for screening<br>✓ Multisectoral approach<br>✓ Integrate CC into all health services<br>✓ Tackle cultural barriers<br>✓ Allocate adequate funding for the VIAC programme<br>✓ Provide adequate equipment<br>✓ Provide adequate transport<br>✓ Provide adequate IEC materials in the local language

PRIMARY HEALTH FACILITY NURSES				
**14**	**15**	**16**	**17**	**18**
**Role in VIAC programme**<br>✓ Programme overseer<br>✓ Mentor<br>✓ Sensitise women for screening	**Role in VIAC programme**<br>✓ Give health information on CC and screening<br>✓ Sensitise/ mobilise women for screening	**Role in VIAC programme**<br>✓ Sensitise/mobilise women for screening<br>✓ Educate women on VIAC	**Role in VIAC programme**<br>✓ Give health information on CC and screening<br>✓ Refer women to Gwanda for screening	**Role in VIAC programme**<br>✓ Give health information on CC and screening<br>✓ Sensitise/mobilise women for screening<br>✓ Refer women to Gwanda for screening
**Period in current role**	**Period in current role**	**Period in current role**	**Period in current role**	**Period in current role**
✓ 24 years	✓ 11 years	✓ 20 years	✓ 12 years	✓ 11 years
**Trained in VIAC?**	**Trained in VIAC?**	**Trained in VIAC?**	**Trained in VIAC?**	**Trained in VIAC?**
✓ Not VIAC trained	✓ Not VIAC trained	✓ Not VIAC trained<br>✓ One nurse in the facility trained	✓ Not VIAC trained	✓ Not VIAC trained
**CC prevalence in catchment area**	**CC prevalence in catchment area**	**CC prevalence in catchment area**	**CC prevalence in catchment area**	**CC prevalence in catchment area**
✓ High burden of CC	✓ High burden of CC	✓ High burden of CC	✓ Low burden of CC	✓ High burden of CC
**Women's knowledge on CC**	**Women's knowledge on CC**	**Women's knowledge on CC**	**Women's knowledge on CC**	**Women's knowledge on CC**
✓ Adequate knowledge on CC	✓ Aware of cervical cancer	✓ Lack adequate knowledge on CC	✓ Lack of adequate knowledge on CC	✓ Lack of adequate knowledge on CC
**Women's knowledge on screening**	**Women's knowledge on screening**	**Women's knowledge on screening**	**Women's knowledge on screening**	**Women's knowledge on screening**
✓ Adequate knowledge on VIAC	✓ Adequate knowledge on VIAC	✓ Aware of VIAC programme<br>✓ Lack adequate knowledge on VIAC	✓ Adequate knowledge on VIAC	✓ Lack adequate knowledge on VIAC
**Available CC service providers**	**Available CC service providers**	**Available CC service providers**	**Available CC service providers**	**Available CC service providers**
✓ Phakama Clinic<br>✓ GPH<br>✓ Outreach services	✓ GPH<br>✓ Outreach services	✓ Outreach services	✓ GPH<br>✓ Outreach services	✓ GPH<br>✓ Phakama clinic<br>✓ Outreach services

**Prevalence of screening**	**Prevalence of screening**	**Prevalence of screening**	**Prevalence of screening**	**Prevalence of screening**
✓ Uptake of screening is average	✓ Uptake of screening is low	✓ Uptake of screening is low	✓ Uptake of screening is low	✓ Uptake of screening is low
**Awareness of CC prevention & control guidelines**	**Awareness of CC prevention & control guidelines**	**Awareness of CC prevention & control guidelines**	**Awareness of CC prevention & control guidelines**	**Awareness of CC prevention & control guidelines**
✓ Aware of ZCCPCS guidelines<br>✓ Feasible to implement	✓ Not aware of ZCCPCS guidelines	✓ Not aware of ZCCPCS guidelines	✓ Not aware of ZCCPCS guidelines	✓ Not aware of ZCCPCS guidelines
**Demand creation strategies**	**Demand creation strategies**	**Demand creation strategies**	**Demand creation strategies**	**Demand creation strategies**
✓ Monthly community awareness campaigns<br>✓ Daily health education talks at clinic<br>✓ Health promoters teach women in their residential wards<br>✓ Weekly meetings to educate staff on VIAC	✓ Health education given to women who come to clinic	✓ Health education given to women who come to clinic	✓ Health education given to women who come to clinic<br>✓ CHWs give education<br>✓ Education given during Health staff domiciliary visits	✓ Health education given to women who come to clinic<br>✓ Dissemination of information to the community through the Health Center Committee
**Barriers to CC screening**	**Barriers to CC screening**	**Barriers to CC screening**	**Barriers to CC screening**	**Barriers to CC screening**
✓ Lack of consistency in outreach services<br>✓ Lack of adequate transport for outreach services<br>✓ VIAC programme under financed<br>✓ Shortage of VIAC trained nurses<br>✓ COVID-19 restrictions<br>✓ Myths and misconceptions<br>✓ Low socio-economic status<br>✓ Religious beliefs	✓ Fear of procedure<br>✓ Inadequate demand creation strategies<br>✓ Financial constraints: Lack of money for transport<br>✓ CHWs lack adequate knowledge on VIAC<br>✓ Myths and misconceptions<br>✓ Religious beliefs<br>✓ Non-availability of IEC materials at local health facilities	✓ Long distances from communities to primary health facility<br>✓ Socio-cultural beliefs<br>✓ Lack of consistency in outreach services<br>✓ Religious beliefs<br>✓ Fear of HIV test<br>✓ Women lack adequate knowledge about CC<br>✓ Women lack adequate knowledge on VIAC<br>✓ Lack of partner support	✓ Gap in outreach service because of partner's requisites<br>✓ Financial constraints: Lack of money for transport<br>✓ Women lack adequate knowledge about CC<br>✓ Socio-cultural beliefs<br>✓ Religious beliefs<br>✓ Lack of partner support	✓ Lack of consistency in outreach services<br>✓ Gap in service provision because of partner's requisites<br>✓ Lack of adequate equipment<br>✓ Women lack adequate knowledge about CC<br>✓ Women lack adequate knowledge on VIAC<br>✓ Negative attitudes towards screening

✓ Apathy<br>✓ Low risk perception<br>✓ Mobility of target population	✓ Gap in service provision because of partner's requisites	✓ Negative staff attitudes<br>✓ Gap in service provision because of partner's requisites<br>✓ Lack of adequate transport for outreach services	✓ Long distances from communities to primary health facility<br>✓ Women too busy<br>✓ Nurses lack adequate knowledge about CC and screening<br>✓ Lack of consistency in outreach services<br>✓ Fear of procedure<br>✓ Fear of a cancer diagnosis<br>✓ Lack of adequate equipment<br>✓ Inaccessibility of treatment services for rural communities	✓ Financial constraints: Lack of money for transport<br>✓ Fear of procedure<br>✓ Modesty issues<br>✓ Lack of support from family members<br>✓ Lack of partner support<br>✓ Negative staff attitudes<br>✓ Inaccessibility of treatment services for rural communities<br>✓ VIAC programme under financed
**Strategies to address barriers**	**Strategies to address barriers**	**Strategies to address barriers**	**Strategies to address barriers**	**Strategies to address barriers**
✓ Involve influential community leaders as important stakeholders in the VIAC programme	✓ Integrate CC into all health services<br>✓ Train CHWs on CC and VIAC<br>✓ All health facilities to provide VIAC services<br>✓ Train nurses at all health facilities on VIAC<br>✓ Provide IEC materials for distribution to the community	✓ Outreach time to keep scheduled times<br>✓ Staff to treat clients professionally<br>✓ Male involvement	✓ Screen every woman<br>✓ Outreach team to open other sites furthest to the clinics<br>✓ Increase outreach services<br>✓ Provide adequate equipment<br>✓ Provide treatment at screening sites	✓ Increase outreach services<br>✓ Have 2 outreach teams<br>✓ Outreach team to provide health facility with screening records for ease of follow up

Community level health workers (urban based)				
**19**	**20**			
**Role in VIAC programme**<br>✓ Sensitise / mobilise women for screening	**Role in VIAC programme**<br>✓ Give health information on CC<br>✓ Sensitise / mobilise women for screening			
**Period in current role**	**Period in current role**			
✓ 3 years	✓ 4 years			
**Trained in VIAC?**	**Trained in VIAC?**			
✓ Has not received training on CC and screening	✓ Has not received training on CC and screening<br>✓ On the job learning			
**CC prevalence in catchment area**	**CC prevalence in catchment area**			
✓ High burden of CC and precancerous conditions	✓ High burden of CC			
**Women's knowledge on CC**	**Women's knowledge on CC**			
✓ Inadequate knowledge on CC	✓ 50 % of women have adequate knowledge on CC<br>✓ 50 % of women have inadequate knowledge on CC			
**Women's knowledge on screening**	**Women's knowledge on screening**			
✓ Inadequate knowledge on VIAC	✓ 50 % of women have adequate knowledge on VIAC<br>✓ 50 % of women have inadequate knowledge on VIAC			

**Available CC service providers**	**Available CC service providers**			
✓ Phakama clinic<br>✓ GPH	✓ Phakama Clinic<br>✓ GPH			
**Prevalence of screening**	**Prevalence of screening**			
✓ Low uptake of screening	✓ Low uptake of screening			
**Awareness of CC prevention & control guidelines**	**Awareness of CC prevention & control guidelines**			
✓ No written guidelines to refer to	✓ No written guidelines to refer to			
**Demand creation strategies**	**Demand creation strategies**			
✓ Use women meeting places to disseminate information	✓ Talk to women at clinic 3 times a week<br>✓ Conduct home visits			
**Barriers to CC screening**	**Barriers to CC screening**			
✓ Socio-cultural beliefs<br>✓ Fear of procedure<br>✓ Women lack adequate knowledge about CC<br>✓ Women lack adequate knowledge on VIAC<br>✓ Women undermine CHWs<br>✓ CHWs lack adequate knowledge on VIAC<br>✓ Religious beliefs<br>✓ Myths and misconceptions<br>✓ Long waiting periods<br>✓ Shortage of VIAC trained nurses	✓ Women lack adequate knowledge about CC<br>✓ Women lack adequate knowledge on VIAC<br>✓ Apathy<br>✓ Women too busy<br>✓ Socio-cultural beliefs<br>✓ Myths and misconceptions			

✓ Fear of stigma and discrimination<br>✓ Lack of partner support<br>✓ Inadequate screening sites				
**Strategies to address barriers**	**Strategies to address barriers**			
✓Increase awareness creation strategies on CC screening<br>✓ Conduct education workshops for women<br>✓ Use innovative strategies for awareness creation<br>✓ Involve influential community leaders as important stakeholders in the VIAC programme<br>✓ Identify and train women as peer educators<br>✓ Education on VIAC should first target community leaders<br>✓ Establish urban outreach clinics<br>✓ Male involvement	✓Increase awareness creation strategies on CC screening<br>✓ Intensify education on CC and screening<br>✓ Train CHWs on CC and VIAC			

Community level health workers (rural based)				
21	22	23	24	25
**Role in VIAC programme**<br>✓ Give health information on CC<br>✓ Sensitise / mobilise women for screening	**Role in VIAC programme**<br>✓ Sensitise / mobilise women for screening	**Role in VIAC programme**<br>✓ Sensitise / mobilise women for screening<br>✓ Sensitise/mobilise girls for HPV vaccination<br>✓ Encourage women who test VIAC positive to go for treatment<br>✓ Keep records of women who have been screened	**Role in VIAC programme**<br>✓ Sensitise / mobilise women for screening<br>✓ Sensitise/mobilise girls for HPV vaccination	**Role in VIAC programme**<br>✓ Create awareness about VIAC<br>✓ Disseminate VIAC information during meetings
**Period in current role**	**Period in current role**	**Period in current role**	**Period in current role**	**Period in current role**
✓ 6 years	✓ 3 years	✓ 19 years	✓ 10 years	✓ 3 years
**Trained in VIAC?**	**Trained in VIAC?**	**Trained in VIAC?**	**Trained in VIAC?**	**Trained in VIAC?**
✓ Has not received training on CC and screening<br>✓On the job learning	✓ Has not received training on CC and screening<br>✓ On the job learning	✓ Has not received training on CC and screening<br>✓ On the job learning	✓ Has not received training on CC and screening	✓ Has not received training on CC and screening
**CC prevalence in catchment area**	**CC prevalence in catchment area**	**CC prevalence in catchment area**	**CC prevalence in catchment area**	**CC prevalence in catchment area**
✓ Low burden of CC	✓ High burden of CC	✓ Low burden of CC precancerous conditions	✓ High burden of CC	✓ Low burden of CC
**Women's knowledge on CC**	**Women's knowledge on CC**	**Women's knowledge on CC**	**Women's knowledge on CC**	**Women's knowledge on CC**
✓ Inadequate knowledge on CC	✓ Young women have adequate knowledge on CC	✓ Adequate knowledge on CC	✓ Knowledge on CC	✓ Inadequate knowledge on CC
**Women's knowledge on screening**	**Women's knowledge on screening**	**Women's knowledge on screening**	**Women's knowledge on screening**	**Women's knowledge on screening**
✓ Inadequate knowledge on VIAC	✓ Young women have adequate knowledge on CC	✓ Adequate knowledge on VIAC	✓ Aware of VIAC programme	✓ Inadequate knowledge on VIAC
**Available CC service providers**	**Available CC service providers**	**Available CC service providers**	**Available CC service providers**	**Available CC service providers**

✓ GPH	✓ GPH<br>✓ Outreach services	✓ GPH<br>✓ Outreach services	✓ GPH<br>✓ Outreach services	✓ GPH<br>✓ Outreach services
**Prevalence of screening**	**Prevalence of screening**	**Prevalence of screening**	**Prevalence of screening**	**Prevalence of screening**
✓Low uptake of screening	✓ Low uptake of screening	✓ Low uptake of screening	✓ Low uptake of screening	
**Awareness of CC prevention & control guidelines**	**Awareness of CC prevention & control guidelines**	**Awareness of CC prevention & control guidelines**	**Awareness of CC prevention & control guidelines**	**Awareness of CC prevention & control guidelines**
✓ No written guidelines to refer to	✓ Has book to refer to	✓ No written guidelines to refer to	✓ No written guidelines to refer to	✓ No written guidelines to refer to
**Demand creation strategies**	**Demand creation strategies**	**Demand creation strategies**	**Demand creation strategies**	**Demand creation strategies**
✓ Educate women on CC at baby weighing points	✓ Educate women on CC at baby weighing points	✓ Educate women on CC at baby weighing points<br>✓ Use community meetings to disseminate information on CC	✓ Conduct home visits<br>✓ Use community meetings to disseminate information on CC	✓ Conduct home visits<br>✓ Educate women on CC at baby weighing points<br>✓ Use Village WhatsApp group<br>✓ Educate women on CC during support group meetings
**Barriers to CC screening**	**Barriers to CC screening**	**Barriers to CC screening**	**Barriers to CC screening**	**Barriers to CC screening**
✓ Women lack adequate knowledge about CC<br>✓ Women lack adequate knowledge on VIAC<br>✓ CHWs lack adequate knowledge on VIAC<br>✓ Inaccessibility of screening services to rural communities<br>✓ Financial constraints: Lack of money for transport	✓ Women lack adequate knowledge about CC<br>✓ Women lack adequate knowledge on VIAC<br>✓ Lack of consistency in outreach services<br>✓ Fear of a cancer diagnosis<br>✓ Negative staff attitudes<br>✓ Negative attitudes towards screening<br>✓ Modesty issues<br>✓ Gender of service provider<br>✓ Religious beliefs	✓ Financial constraints: Lack of money for transport and treatment<br>✓ Gap in outreach service because of partner requisites<br>✓ Inaccessibility of screening services to rural communities<br>✓ Inaccessibility of treatment services for rural communities<br>✓ Lack of consistency in outreach services	✓ Inaccessibility of screening services to rural communities<br>✓ Financial constraints: Lack of money for transport and treatment<br>✓ CHWs lack adequate knowledge on VIAC<br>✓ Socio-cultural beliefs<br>✓ Apathy<br>✓ Long distances from communities to primary health facility	✓ Inaccessibility of screening services to rural communities<br>✓ Women lack adequate knowledge about CC<br>✓ Women lack adequate knowledge on VIAC<br>✓ Apathy<br>✓ Lack of consistency in outreach services<br>✓ Financial constraints: Lack of money for transport

	✓ Lack of partner support<br>✓ Low risk perception<br>✓ Financial constraints: Lack of money for transport and treatment<br>✓ Gap in outreach service because of partner's requisites	✓ Fear of stigma and discrimination<br>✓ Religious beliefs<br>✓ CHWs lack adequate knowledge on VIAC<br>✓ Non-availability of IEC materials	✓ Fear of a cancer diagnosis	✓ Gap in outreach service because of partner requisites<br>✓ Modesty issues<br>✓ CHWs lack adequate knowledge on VIAC<br>✓ Women undermine CHWs<br>✓ Low risk perception<br>✓ Myths and misconceptions
**Strategies to address barriers**	**Strategies to address barriers**	**Strategies to address barriers**	**Strategies to address barriers**	**Strategies to address barriers**
✓ Well informed people to deliver information on VIAC to mothers<br>✓ All health facilities to provide screening<br>✓ Intensify education on CC and screening<br>✓ Community Involvement for acceptability of the programme<br>✓ Train CHWs on CC and VIAC	✓ Outreach to give their visit dates in advance<br>✓ Intensify education on CC and screening<br>✓ Increase outreach services<br>✓ Provide CHWs with CC promotional materials	✓ Screen every woman<br>✓ Provide treatment at outreach screening sites<br>✓ Increase outreach services<br>✓ Distribute IEC materials to women<br>Health facilities to provide CHWs with screening records for ease of follow up	✓ Train CHWs on CC and screening<br>✓ Increase outreach services<br>✓ Intensify education on CC and screening<br>✓ All health facilities to provide screening services<br>✓ Integrate medical and indigenous knowledge	✓ Screen every woman<br>✓ Find different method of screening<br>✓ Male involvement<br>✓ Train CHWs on CC and VIAC<br>✓ Refresher courses for CHWs<br>✓ Well informed people to deliver information on VIAC to mothers<br>✓ Increase outreach services<br>✓ All health facilities to provide VIAC services

EMERGING THEMES BASED ON THE THEORETICAL FRAMEWORK

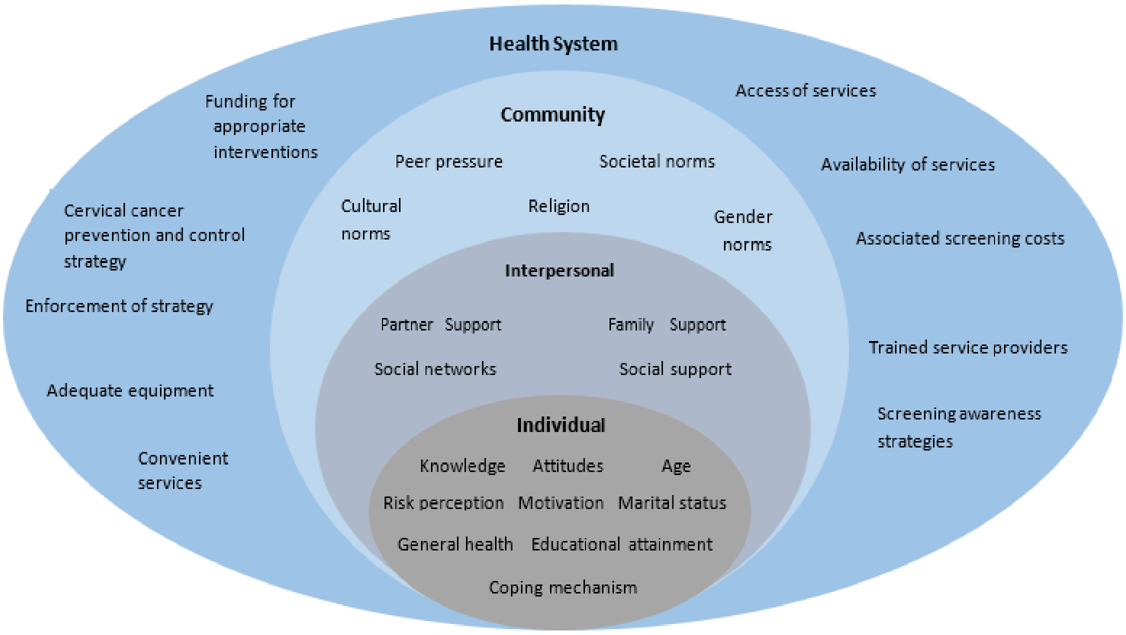

Main theme	Sub-themes
Health System factors	✓ Lack of consistency in outreach services (11)<br>✓ Gap in outreach service due to partner's requisites (11)<br>✓ Inaccessibility of screening services to rural communities (10)<br>✓ Shortage of VIAC trained nurses (9)<br>✓ VIAC programme underfunded – general lack of screening resources (9)<br>✓ Lack of adequate screening equipment (8)<br>✓ Nurses and CHWs lack adequate knowledge on cervical cancer and VIAC (11)<br>✓ Lack of adequate transport for outreach services (5)<br>✓ Inaccessibility of treatment services for rural communities (5)<br>✓ Inadequate screening sites (5)<br>✓ Inadequate demand creation strategies (5)<br>✓ VIAC nurses multitasking (4)<br>✓ Negative staff attitudes (4)<br>✓ LEEP specimens tested at higher level laboratories – delay in instituting treatment (3)<br>✓ VIAC clinic closed during weekend and public holidays (3)<br>✓ Poor infrastructure (3)<br>✓ Long distances from communities to primary health facilities (3)<br>✓ VIAC nurses lack motivation (2)<br>✓ Long waiting periods (2)<br>✓ Non- availability of IEC materials (2)<br>✓ Loss to follow up (2)<br>✓ Power outages (1)<br>✓ Nurses lack confidentiality (1)
Individual factors	✓ Women lack adequate knowledge about cervical cancer (18)<br>✓ Women lack adequate knowledge about VIAC (18)<br>✓ Financial constraints (15)

	✓ Fear of procedure (7)<br>✓ Fear of a cancer diagnosis (6)<br>✓ Low risk perception (6)<br>✓ Women lack awareness on VIAC (6)<br>✓ Apathy (5)<br>✓ Negative attitudes towards screening (4)<br>✓ Poor health seeking behaviours (2)<br>✓ Women too busy (2)<br>✓ Lack of trust in health services (2)<br>✓ Fear of HIV test (1)
Community factors	✓ Socio-cultural beliefs (10)<br>✓ Religious beliefs (10)<br>✓ Myths and misconceptions (6)<br>✓ Fear of stigma and discrimination (5)<br>✓ Modesty issues (3)<br>✓ Women undermine VCWs (2)<br>✓ Negative peer influence (1)<br>✓ Gender of service provider (1)
Interpersonal factors	✓ Lack of partner support (9)<br>✓ Lack of support of from family members (1)
	✓ COVID-19 restrictions (5)<br>✓ Low socio-economic status (3)<br>✓ Mobility of target population (2)

Objective 5:

To examine existing strengths and facilitators that could be incorporated into the cervical cancer screening programme in Gwanda district

1. Resident Consultant Obstetrician and Gynecologist: VIAC training now done at Gwanda Provincial Hospital
2. Integrated outreach team to rural communities
3. Collaboration with community leaders
4. Private practice offering VIAC services
5. Women's acceptance of the VIAC programme: Outreach attendance high at outreach clinics
6. Women from Religious groups which don't allow members to access health services sneak out to seek health services
7. Media awareness campaigns ongoing
8. Community Health Workers getting nice incentives (motivated)
9. Free screening and treatment
